# Publisher Correction: Genome-wide association study reveals mechanisms underlying dilated cardiomyopathy and myocardial resilience

**DOI:** 10.1038/s41588-024-02047-4

**Published:** 2024-12-04

**Authors:** Sean J. Jurgens, Joel T. Rämö, Daria R. Kramarenko, Leonoor F. J. M. Wijdeveld, Jan Haas, Mark D. Chaffin, Sophie Garnier, Liam Gaziano, Lu-Chen Weng, Alex Lipov, Sean L. Zheng, Albert Henry, Jennifer E. Huffman, Saketh Challa, Frank Rühle, Carmen Diaz Verdugo, Christian Krijger Juárez, Shinwan Kany, Constance A. van Orsouw, Kiran Biddinger, Edwin Poel, Amanda L. Elliott, Xin Wang, Catherine Francis, Richard Ruan, Satoshi Koyama, Leander Beekman, Dominic S. Zimmerman, Jean-François Deleuze, Eric Villard, David-Alexandre Trégouët, Richard Isnard, FinnGen, Joel T. Rämö, Amanda L. Elliott, Juha Sinisalo, Teemu Niiranen, Jari Laukkanen, Aarno Palotie, Mark Daly, VA Million Veteran Program, Jennifer E. Huffman, Kyong-Mi Chang, Philip S. Tsao, Krishna G. Aragam, HERMES Consortium, Sean L. Zheng, Albert Henry, Kiran Biddinger, James S. Ware, R. Thomas Lumbers, Patrick T. Ellinor, Krishna G. Aragam, Dorret I. Boomsma, Eco J. C. de Geus, Rafik Tadros, Yigal M. Pinto, Arthur A. M. Wilde, Jouke-Jan Hottenga, Juha Sinisalo, Teemu Niiranen, Roddy Walsh, Amand F. Schmidt, Seung Hoan Choi, Kyong-Mi Chang, Philip S. Tsao, Paul M. Matthews, James S. Ware, R. Thomas Lumbers, Saskia van der Crabben, Jari Laukkanen, Aarno Palotie, Ahmad S. Amin, Philippe Charron, Benjamin Meder, Patrick T. Ellinor, Mark Daly, Krishna G. Aragam, Connie R. Bezzina

**Affiliations:** 1 Department of Experimental Cardiology, Amsterdam Cardiovascular Sciences, Heart Failure & Arrhythmias, Amsterdam UMC location, University of Amsterdam, Amsterdam, the Netherlands; 2 Cardiovascular Disease Initiative, Broad Institute of MIT and Harvard, Cambridge, MA USA; 3 Cardiovascular Research Center, Massachusetts General Hospital, Boston, MA USA; 4 Institute for Molecular Medicine Finland (FIMM), Helsinki Institute of Life Science (HiLIFE), University of Helsinki, Helsinki, Finland; 5 European Reference Network for rare low prevalence and complex diseases of the heart: ERN GUARD-Heart, Amsterdam, the Netherlands; 6 Department of Physiology, Amsterdam UMC location, Vrije Universiteit, Amsterdam, the Netherlands; 7 Department of Medicine III, Institute for Cardiomyopathies Heidelberg (ICH), University Hospital Heidelberg, Heidelberg, Germany; 8 Site Heidelberg/Mannheim, DZHK, Heidelberg, Germany; 9 Research Unit on Cardiovascular Disorders, Metabolism and Nutrition, Team Genomics and Pathophysiology of Cardiovascular Disease, Sorbone Université, INSERM, Paris, France; 10 ICAN Institute for Cardiometabolism and Nutrition, Paris, France; 11 National Heart and Lung Institute, Imperial College London, London, UK; 12 MRC Laboratory of Medical Sciences, Imperial College London, London, UK; 13 Royal Brompton and Harefield Hospitals, Guy’s and St. Thomas’ NHS Foundation Trust, London, UK; 14 Institute of Cardiovascular Science, University College London, London, UK; 15 Institute of Health Informatics, University College London, London, UK; 16 Massachusetts Veterans Epidemiology Research and Information Center (MAVERIC), VA Boston Healthcare System, Boston, MA USA; 17 Palo Alto Veterans Institute for Research (PAVIR), Palo Alto Health Care System, Palo Alto, CA USA; 18 Harvard Medical School, Boston, MA USA; 19 Bioinformatics Core Facility, Institute of Molecular Biology gGmbH (IMB), Mainz, Germany; 20 Department of Genetic Epidemiology, Institute of Human Genetics, University of Münster, Münster, Germany; 21 Department of Cardiology, University Heart and Vascular Center, University Medical Center Hamburg-Eppendorf, Hamburg, Germany; 22 Department of Clinical Genetics, Amsterdam UMC location, University of Amsterdam, Amsterdam, the Netherlands; 23 Department of Psychiatry and Center for Genomic Medicine, Psychiatric and Neurodevelopmental Genetics Unit, Massachusetts General Hospital, Harvard Medical School, Boston, MA USA; 24 Program in Medical and Population Genetics, Broad Institute of Harvard and MIT, Cambridge, MA USA; 25 Stanley Center for Psychiatric Research, Broad Institute of Harvard and MIT, Cambridge, MA USA; 26 CEA, Centre National de Recherche en Génomique Humaine, Université Paris-Saclay, Evry, France; 27 Laboratory of Excellence in Medical Genomics, GENMED, Evry, France; 28 Fondation Jean Dausset, Centre d’Etude du Polymorphisme Humain, Paris, France; 29 Bordeaux Population Health Research Center, UMR 1219, University of Bordeaux, INSERM, Bordeaux, France; 30 APHP, Cardiology and Genetics Departments, Pitié-Salpêtrière Hospital, Paris, France; 31 Department of Biological Psychology, Vrije Universiteit Amsterdam, Amsterdam, the Netherlands; 32 Amsterdam Public Health Research Institute, Amsterdam UMC location, Vrije Universiteit, Amsterdam, the Netherlands; 33 Cardiovascular Genetics Centre, Montreal Heart Institute, Montreal, QC Canada; 34 Faculty of Medicine, Université de Montréal, Montreal, QC Canada; 35 Department of Clinical Cardiology, Amsterdam Cardiovascular Sciences, Heart Failure and Arrhythmias, Amsterdam UMC location, University of Amsterdam, Amsterdam, the Netherlands; 36 The Netherlands Twin Register, Vrije Universiteit Amsterdam, Amsterdam, the Netherlands; 37 Department of Cardiology, Helsinki University Hospital, Helsinki, Finland; 38 Heart and Lung Center, Helsinki University Hospital and Helsinki University, Helsinki, Finland; 39 Department of Internal Medicine, University of Turku, Helsinki, Finland; 40 Division of Medicine, Turku University Hospital, Helsinki, Finland; 41 Finnish Institute for Health and Welfare (THL), Helsinki, Finland; 42 Institute of Cardiovascular Science, Faculty of Population Health, University College London, London, UK; 43 University College London British Heart Foundation Research Accelerator, London, UK; 44 Department of Cardiology, Division Heart and Lungs, University Medical Center Utrecht, Utrecht University, Utrecht, the Netherlands; 45 Department of Biostatistics, Boston University, Boston, MA USA; 46 Corporal Michael J. Crescenz VA Medical Center, Philadelphia, PA USA; 47 Department of Medicine, University of Pennsylvania Perelman School of Medicine, Philadelphia, PA USA; 48 Palo Alto Health Care System, Palo Alto, CA USA; 49 Department of Medicine and Cardiovascular Institute, Stanford University School of Medicine, Stanford, CA USA; 50 The National Institute for Health Research, University College London Hospitals Biomedical Research Centre, University College London, London, UK; 51 Department of Medicine, Institute of Clinical Medicine, University of Eastern Finland, Kuopio, Finland; 52 Central Finland Biobank, Central Finland Health Care District, Jyväskylä, Finland; 53 Program in Medical and Population Genetics and Stanley Center for Psychiatric Research, Broad Institute of Harvard and MIT, Cambridge, MA USA; 54 Analytic and Translational Genetics Unit, Massachusetts General Hospital, Boston, MA USA

**Keywords:** Cardiomyopathies, Genome-wide association studies, Heart failure

Correction to: *Nature Genetics* 10.1038/s41588-024-01975-5, published online 21 November 2024.

In the version of the article initially published, in the Methods, reference numbers were displayed one number lower than they should have been. This has now been amended in the HTML and PDF versions of the article.

