# Supplementary material for: Genome-wide association study reveals mechanisms underlying dilated cardiomyopathy and myocardial resilience
Source: Nat Genet. 2024 Nov 21;56(12):2636–45. doi: 10.1038/s41588-024-01975-5 (PMC11631763; doi:10.1038/s41588-024-01975-5)
Supplement: Supplementary file 1 — Supplementary Notes and Figs. 1–13. [file 41588_2024_1975_MOESM1_ESM.pdf]

nature genetics

Supplementary information

https://doi.org/10.1038/s41588-024-01975-5

Genome-wide association study reveals mechanisms underlying dilated cardiomyopathy and myocardial resilience

In the format provided by the authors and unedited

Supplementary information for

Genome-wide association study reveals mechanisms underlying dilated cardiomyopathy and myocardial resilience

Sean J Jurgens[1,2,3,†], Joel T Rämö[2,3,4†], Daria R Kramarenko[1,5,†], Leonoor FJM Wijdeveld[2,6,†], Jan Haas[7,8,†], Mark D Chaffin[2,†], Sophie Garnier[9,10,†], Liam Gaziano[2,3,†], Lu-Chen Weng[2,3], Alex Lipov[1], Sean L Zheng[11,12,13], Albert Henry[14,15], Jennifer E Huffman[16,17,18], Saketh Challa[2], Frank Rühle[19,20], Carmen Diaz Verdugo[2], Christian Krijger Juárez[1], Shinwan Kany[2,3,21], Constance A van Orsouw[22], Kiran Biddinger[2], Edwin Poel[1], Amanda L Elliott[4,18,23,24,25], Xin Wang[2], Catherine Francis[11,13], Richard Ruan[2], Satoshi Koyama[2,3], Leander Beekman[1], Dominic S Zimmerman[1], Jean-François Deleuze[26,27,28], Eric Villard[9,10], David-Alexandre Trégouët[27,29], Richard Isnard[9,10,30], FinnGen, VA Million Veteran Program, HERMES Consortium, Dorret I Boomsma[31], Eco JC de Geus[31,32], Rafik Tadros[1,33,34], Yigal M Pinto[1,5,35], Arthur AM Wilde[1,5,35], Jouke-Jan Hottenga[31,36], Juha Sinisalo[37,38], Teemu Niiranen[39,40,41], Roddy Walsh[1], Amand F Schmidt[35,42,43,44], Seung Hoan Choi[2,45], Kyong-Mi Chang[46,47], Philip S Tsao[48,49], Paul M Matthews[11,12], James S Ware[11,12,13,24], R Thomas Lumbers[15,50], Saskia van der Crabben[22], Jari Laukkanen[51,52,*], Aarno Palotie[4,53,54,*], Ahmad S Amin[1,5,35,*], Philippe Charron[9,10,30,*], Benjamin Meder[7,8,*], Patrick T Ellinor[2,3,*,#], Mark Daly[4,53,54,*,#], Krishna G Aragam[2,3,*,#], Connie R Bezzina[1,5,*,#]

[1]Department of Experimental Cardiology, Amsterdam Cardiovascular Sciences, Heart Failure & Arrhythmias, Amsterdam UMC location University of Amsterdam, Amsterdam, Netherlands, [2]Cardiovascular Disease Initiative, Broad Institute of MIT and Harvard, Cambridge, MA, USA, [3]Cardiovascular Research Center, Massachusetts General Hospital, Boston, USA, [4]Institute for Molecular Medicine Finland (FIMM), Helsinki Institute of Life Science (HiLIFE), University of Helsinki, Helsinki, Finland, [5]European Reference Network for rare, low prevalence and complex diseases of the heart: ERN GUARD-Heart, [6]Department of Physiology, Amsterdam UMC location Vrije Universiteit, Amsterdam, Netherlands, [7]Department of Medicine III, Institute for Cardiomyopathies Heidelberg (ICH.), University Hospital Heidelberg, Heidelberg, Germany, [8]Site Heidelberg/Mannheim, DZHK, Heidelberg, Germany, [9]Research Unit on Cardiovascular Disorders, Metabolism and Nutrition, Team Genomics & Pathophysiology of Cardiovascular Disease, Sorbonne Universite, INSERM, UMR-S1166, Paris, France, [10]ICAN Institute for Cardiometabolism and Nutrition, Paris, France, [11]National Heart & Lung Institute, Imperial College London, London, UK, [12]MRC Laboratory of Medical Sciences, Imperial College London, London, UK, [13]Royal Brompton & Harefield Hospitals, Guy's and St. Thomas' NHS Foundation Trust, London, UK, [14]Institute of Cardiovascular Science, University College London, London, UK, [15]Institute of Health Informatics, University College London, London, UK, [16]Massachusetts Veterans Epidemiology Research and Information Center (MAVERIC), VA Boston Healthcare System, Boston, MA, USA, [17]Palo Alto Veterans Institute for Research (PAVIR), Palo Alto Health Care System, Palo Alto, CA, USA, [18]Harvard Medical School, Boston, MA, USA, [19]Bioinformatics Core Facility, Institute of Molecular Biology gGmbH (IMB), Mainz, Germany, [20]Department of Genetic Epidemiology, Institute of Human Genetics, University of Münster, Münster, Germany, [21]Department of Cardiology, University Heart and Vascular Center, University Medical Center Hamburg-

Eppendorf, Hamburg, Germany, [22]Department of Clinical Genetics, Amsterdam UMC location University of Amsterdam, Amsterdam, Netherlands, [23]Psychiatric and Neurodevelopmental Genetics Unit, Department of Psychiatry and Center for Genomic Medicine, Massachusetts General Hospital, Harvard Medical School, Boston, MA, USA, [24]Program in Medical and Population Genetics, Broad Institute of Harvard and MIT, Cambridge, MA, USA, [25]Stanley Center for Psychiatric Research, Broad Institute of Harvard and MIT, Cambridge, MA, USA, [26]CEA, Centre National de Recherche en Génomique Humaine, Université Paris-Saclay, Evry, France, [27]Laboratory of Excellence in Medical Genomics, GENMED, Evry, France, [28]Fondation Jean Dausset, Centre d'Etude du Polymorphisme Humain, Paris, France, [29]Bordeaux Population Health Research Center, UMR 1219, Univeristy of Bordeaux, INSERM, Bordeaux, France, [30]APHP, Cardiology & Genetics Departments, Pitié-Salpêtrière Hospital, Paris, France, [31]Department of Biological Psychology, Vrije Universiteit Amsterdam, Amsterdam, Netherlands, [32]Amsterdam Public Health Research Institute, Amsterdam UMC location Vrije Universiteit, Amsterdam, Netherlands, [33]Cardiovascular Genetics Centre, Montreal Heart Institute, Montreal, QC, Canada, [34]Faculty of Medicine, Université de Montréal, Montreal, QC, Canada, [35]Department of Clinical Cardiology, Amsterdam Cardiovascular Sciences, Heart Failure & Arrhythmias, Amsterdam UMC location University of Amsterdam, Amsterdam, Netherlands, [36]Netherlands Twin Register, Vrije Universiteit Amsterdam, Amsterdam, Netherlands, [37]Department of Cardiology, Helsinki University Hospital, Helsinki, Finland, [38]Heart and Lung Center, Helsinki University Hospital and Helsinki University, Helsinki, Finland, [39]Department of Internal Medicine, University of Turku, Turku, Helsinki, Finland, [40]Division of Medicine, Turku University Hospital, Turku, Helsinki, Finland, [41]Finnish Institute for Health and Welfare (THL), Helsinki, Finland, [42]Institute of Cardiovascular Science, Faculty of Population Health, University College London, London, UK, [43]University College London British Heart Foundation Research Accelerator, London, UK, [44]Department of Cardiology, Division Heart and Lungs, University Medical Center Utrecht, Utrecht University, Utrecht, Netherlands, [45]Department of Biostatistics, Boston University, Boston, MA, USA, [46]Corporal Michael J. Crescenz VA Medical Center, Philadelphia, PA, USA, [47]Department of Medicine, University of Pennsylvania Perelman School of Medicine, Philadelphia, PA, USA, [48]Palo Alto Health Care System, Palo Alto, CA, USA, [49]Department of Medicine & Cardiovascular Institute, Stanford University School of Medicine, Stanford, CA, USA, [50]The National Institute for Health Research University College London Hospitals Biomedical Research Centre, University College London, London, UK, [51]Central Finland Biobank, Central Finland Health Care District, Jyväskylä, Finland, [52]Department of Medicine, Institute of Clinical Medicine, University of Eastern Finland, Kuopio, Finland, [53]Program in Medical and Population Genetics and Stanley Center for Psychiatric Research, Broad Institute of Harvard and MIT, Cambridge, MA, USA, [54]Analytic and Translational Genetics Unit, Massachusetts General Hospital, Boston, MA, USA

† These authors contributed equally
* These authors jointly supervised the work
# Corresponding authors

Correspondence to:
ellinor@mgh.harvard.edu
mark.daly@helsinki.fi
karagam@broadinstitute.org
c.r.bezzina@amsterdamumc.nl

Table of Contents

Supplementary Note **4**
Garnier et al. cohort description and methods 4
Meder et al. cohort description and re-analysis methods 7
Amsterdam UMC Cohort study description and methods 8
Cases 8
Controls 9
Quality control and processing 10
GWAS 11
PRS analyses 12
FinnGen study description and methods 14
FinnGen Ethics Statement 16
FinnGen Acknowledgements 18
FinnGen banner authors 19
UK Biobank study description and methods 35
Massachusetts General Brigham Biobank study description and methods 36
All of Us Research Program description and methods 38
Sequencing and quality control 38
Principal component analysis 40
Ancestry assignments 40
Phenotype definitions 41
Acknowledgements 42
Processing of summary statistics for molecular trait MR and colocalization 43
Processing of summary statistics for MR analysis 44
Assessment of NI-DCM and NICM phenotypes from biobank data 45
Novelty of significant loci 46
Assessment of pleiotropy for significant loci 47
Replication analyses 48
HERMES 48
MVP 49
All of Us 52
Meta-analysis and quality-control 52
Power calculations 53
Replication rates and results 54
HERMES banner authors 56
MVP banner authors 65
Cell type enrichment analysis using the Chaffin et al. snRNAseq dataset 67
Cell type specific gene programs 67
s-LDSC analysis of cell type specific gene programs 68
s-LDSC analysis of disease-dependent gene programs 68
Cell type enrichment and differential expression analyses in the Reichart et al. snRNAseq dataset .. 69
Cell type specific gene programs 69
s-LDSC analysis of cell type specific gene programs 70

Differential expression analysis of DCM versus controls 70
s-LDSC analysis of disease-dependent gene programs 71
Harmonization of cell types across single cell datasets to construct LV expression patterns 72
Comparison with results from Zheng et al. 73
Genes prioritized in overlapping loci 73
Cell type enrichments 74
Polygenic score prediction 78
Causal consequences of DCM liability 80
Supplementary Tables 81
Supplementary Figures and Figure Legend 82
Supplementary Figure 1: Quantile-quantile plots of contributing studies to the meta-analyses of NI-DCM, NICM and clinical DCM. 82
Supplementary Figure 2: Manhattan plots for biobank datasets and newly-analyzed clinical datasets for NI-DCM / clinical DCM 84
Supplementary Figure 3: Manhattan plots for biobank datasets for NICM 86
Supplementary Figure 4: Matrix of genetic correlations between DCM, other cardiomyopathic diseases, and left ventricular traits from cardiac MRI, estimated using MTAG. 88
Supplementary Figure 5: Tissue enrichment of DCM heritability from bulk RNA sequencing data in GTEx v8. 90
Supplementary Figure 6: Genotype cluster plots for the TUBA8 variant 22:18609493:G:A in FinnGen 91
Supplementary Figure 7: Results from pathway enrichment analysis using g:Profiler. 92
Supplementary Figure 8: Plots for Mendelian randomization analyses for weight->DCM. 93
Supplementary Figure 9: Plots for Mendelian randomization analyses for BMI->DCM. 95
Supplementary Figure 10: Plots for Mendelian randomization analyses for systolic blood pressure->DCM. 97
Supplementary Figure 11: Plots for Mendelian randomization analyses for DCM->heart failure. 99
Supplementary Figure 12: Associations between DCM polygenic risk score and DCM across various subsets of the Amsterdam UMC dataset. 101
Supplementary Figure 13: Results from cell type enrichment analysis using disease-dependent gene programs. 102
Supplementary References 103

Supplementary Note

Garnier et al. cohort description and methods

The Garnier et al. 2021 (ref.[1]) cohort consisted of 2,719 sporadic DCM cases - from 5 European ancestry sub-populations (France, Germany, Italy, United Kingdom, and the USA) - and 4,440 controls from 3 European sub-populations (France, Germany and Italy). Sporadic DCM was diagnosed by reduced ejection fraction (echocardiography: <45% or MRI: <2 standard deviations (SDs) below the age- and sex-adjusted mean) and an enlarged left ventricle end-diastolic volume/diameter (LVEDD >117% of value predicted from age and body surface area on echocardiography, or >2 SDs from the age- and sex-adjusted mean by magnetic resonance imaging). Cases required that there was absence of significant coronary artery disease or intrinsic valvular disease, documented myocarditis, systemic disease (such as sarcoidosis), sustained arterial hypertension, or congenital malformation. Details on all participating case and control sets are described in detail elsewhere[1], although we give an overview of case criteria in the next paragraph. Of note, the German cases originated from Berlin while cases from the Meder cohort (described below) were ascertained from Heidelberg[2]. All patients signed informed consent, the study protocol was approved by local ethics committees and complies with the Declaration of Helsinki.

The dataset consisted of several sub-cohorts, that had the following inclusion | exclusion criteria. French Cardigene (N=408): LVEDD > 140 ml/m2 on ventriculography or > 34 mm/m2 on echocardiography LVEF ≤ 40% | Absence of causal factors such as coronary artery disease or sustained arterial hypertension, intrinsic valvular disease, documented myocarditis, congenital malformation, and insulin-dependent diabetes. French PHRC (N=204): LVEDD > 117% of predicted value according to age and body surface area on echocardiography LVEF <

45% | Absence of causal factors such as coronary artery disease or intrinsic valvular disease, documented myocarditis, or congenital malformation. French Eurogene (N=83): LVEDD > 117% of predicted value according to age and body surface area on echocardiography LVEF < 45% | Absence of causal factors such as coronary artery disease or intrinsic valvular disease, documented myocarditis, systemic disease, sustained rapid supraventricular arrhythmia, or congenital malformation. Italy Eurogene (N=82): LVEDD > 117% of predicted value according to age and body surface area on echocardiography LVEF < 45% | Absence of causal factors such as coronary artery disease or intrinsic valvular disease, documented myocarditis, systemic disease, sustained rapid supraventricular arrhythmia, or congenital malformation. German Eurogene (N=214): LVEDD > 117% of predicted value according to age and body surface area on echocardiography LVEF < 45% | Absence of causal factors such as coronary artery disease or intrinsic valvular disease, documented myocarditis, systemic disease, sustained rapid supraventricular arrhythmia, or congenital malformation. Germany Berlin (N=987): LVEF < 45% | Absence of major coronary artery disease, significant valvular heart disease, hypertensive heart disease, congenital heart disease, myocarditis or other secondary forms of heart failure. UK Royal Brompton (N=109): LVEF < 2sd below and LVEDV > 2sd above the mean normalized for age and sex | Absence of active myocarditis or evidence of infiltrative disease or significant coronary artery disease. US MAGNet: LVEF < 40% | Absence of hypertension, primary valvular disease, or coronary artery disease.

Genotyping, quality control, and analysis were performed as described previously. Genotyping was performed with high-density arrays, followed by imputation to the 1000 Genomes reference dataset[3]. Details on genotyping arrays used can be found in **Supplementary Table 2** and the

previous publication[1]. In short, quality control was performed in each sub-population separately as described in **Supplementary Table 2**, mainly removing variants with minor allele frequency (MAF) <1%, missingness >1%, hardy-weinberg equilibrium test P<0.00001. Subsequently, all autosomal SNP data were merged and the same filters were used to identify high-quality genotyped SNPs (n = 557,776) shared in all individuals of the discovery cohorts. This procedure identified 7 pairs of duplicated individuals, for which only one individual per pair was then kept, and 13 individuals with call rate <99% that were later discarded. A further round of QC was performed to identify additional genetic outliers. Based on the analysis of the identity-by-state distance matrix, 149 additional samples (44 cases and 105 controls) were discarded leaving 6,980 (2,651 cases and 4,329 controls) individuals for imputation and association analysis. To minimize the risk of ambiguous variants (A/T or G/C), these were removed prior to imputation analyses leaving 554,257 autosomal variants. In current work, we focus only on the autosomal variants. The QCed genotyped samples were then put forward for imputation to the 1000 Genomes phase 3 version 5 reference panel (**Supplementary Table 2**) leaving a total of 47,109,465 autosomal imputed variants of which 8,945,129 had imputation quality greater than 0.5, were bi-allelic, presented a minor allele frequency (MAF) higher than 0.005 and were then kept for association analysis.

Genome-wide association analyses for DCM were performed using a logistic regression model adjusted for sex and genome-wide genotype-derived principal components under the assumption of additive allele effects (**Supplementary Table 2**).

Meder et al. cohort description and re-analysis methods

The German cohort of 909 clinical DCM cases and 2120 controls has been described previously in Meder et al. 2014 (ref.[2]). DCM cases were ascertained from Heidelberg Germany, while the majority of controls came from PopGen (N=1644) and a minority from KORA (N=476). Dilated cardiomyopathy was diagnosed according to previous guidelines of the World Health Organization[4], where inclusion criteria for cases included at least moderately (left ventricular ejection fraction < 45%) reduced left ventricular systolic function (assessed by echocardiography or left ventricular angiography) in the absence of a relevant coronary artery disease (CAD). The study was conducted in accordance with the principles of the Declaration of Helsinki. Details on genotyping and quality control are presented in **Supplementary Table 2**. All participants of the Meder cohort provided written informed consent and the study was approved by the ethics committees of the participating study centers.

In the initial description, the GWAS was performed on genotyping array variants only and no correction for ancestral principal components (PCs) was applied. For the current analysis, the data were re-analyzed to i) include genome-wide imputation and ii) correct for PCs. The QC criteria for variants prior to imputation were: minor allele frequency >= 3% in cases and controls, call rate >= 95%, and thresholds for deviation from Hardy-Weinberg equilibrium test were $p < 0.001$ in cases and $p < 0.05$ in controls. The PCA-function of plink version 1.9 (ref.[5]) was used to calculate principal components. Phasing of genotypes was done using SHAPEIT2 (ref.[5,6]) after which imputation was performed using IMPUTE2 (ref.[7]) with the 1000 Genomes Pilot + HapMap 3 set as reference (NCBI build b36, Haplotype release date Jun 2010 / Feb 2009). Association testing was performed using SNPTEST v2.5-beta4 (frequentist 1, method score, ref.[8]) and was adjusted for age, gender and the first 2 principal components of ancestry. SNPs were filtered for minor allele frequency > 1%, call rate >= 95% and thresholds for deviation from HWE were $p < 0.001$ in cases and $p < 0.05$ in controls.

Amsterdam UMC Cohort study description and methods

Cases

Patients with DCM in the Amsterdam UMC cohort were defined as index patients referred by secondary or tertiary cardiologists to the Amsterdam UMC for genetic testing for DCM. The diagnosis of DCM was confirmed for all patients by manual examination of the medical chart, with a required left ventricular ejection fraction (LVEF) of less than 50% to indicate hypocontractility[9]. Other inclusion criteria included the presence of a DNA sample for genotyping and an age older than 18 years. A total of 1,035 cases met these criteria and were selected for further analysis. While the dataset on ventricular dimensions (extracted from EHR) was not entirely complete, being limited to 53% of the samples, within this group a notable 95% exhibited an enlargement of the ventricular chamber that was (stringently) consistent with dilated cardiomyopathy (DCM). This was based on the thresholds for the left ventricular end-diastolic volume index (LVEDVi ≥ 75 ml/m2 for males, LVEDVi ≥ 62 ml/m2 for females) and the left ventricular end-diastolic diameter (LVEDD > 58 mm for males, > 52 mm for females). Importantly, in instances where only the left ventricular end-diastolic volume (LVEDV) was available without corresponding body surface area (BSA) information, the missing data was substituted with a constant value of 2.5 to preclude underestimation. Genotyping was performed using the Illumina Global Screening Array. The study of DCM patients from Amsterdam UMC was performed under a waiver - approved by the Medical Ethical Committee of Amsterdam UMC - allowing genotyping and genome-wide association study of individuals affected by cardiovascular disease.

Controls

The control group was sourced from the Dutch Twin Register (NTR)[10]. Since 1987, NTR has been accumulating information on twins and triplets, either when the parents of newborn twins voluntarily register or when adult twins and their family participate. Ethical clearance for this study has been granted by the Central Ethics Committee on Research Involving Human Subjects at the VU University Medical Centre in Amsterdam, which is an Institutional Review Board certified by the U.S. Office of Human Research Protections. The approval carries the IRB number IRB-2991 under Federal-wide Assurance-3703 and includes specific institute codes (94/105, 96/205, 99/068, 2003/182, 2010/359). All participants, or their parents, have given their informed consent to be part of NTR. For this study we have received data from unrelated individuals from NTR. NTR samples were genotyped using the Illumina Global Screening Array.

Data collection in the NTR was supported by NWO: Twin-family database for behavior genetics and genomics studies (480-04-004); “Spinozapremie” (NWO/SPI 56-464-14192; “Genetic and Family influences on Adolescent psychopathology and Wellness” (NWO 463-06-001); “A twin-sib study of adolescent wellness” (NWO-VENI 451-04-034); ZonMW “Genetic influences on stability and change in psychopathology from childhood to young adulthood” (912-10-020); “Netherlands Twin Registry Repository” (480-15-001/674); “Biobanking and Biomolecular Resources Research Infrastructure” (BBMRI –NL (184.021.007 and 184.033.111). We acknowledge FP7-HEALTH-F4-2007, grant agreement no 201413 (ENGAGE), and the FP7/2007-2013 funded ACTION (grant agreement no 602768) and the European Research Council (ERC-230374). Part of the genotyping was funded by the Genetic Association Information Network (GAIN) of the Foundation for the National Institutes of Health, Rutgers University Cell and DNA Repository (NIMH U24 MH068457-06), the Avera Institute, Sioux Falls, South Dakota (USA) and the National Institutes of Health (NIH R01 HD042157-01A1, MH081802, Grand Opportunity grants 1RC2 MH089951 and 1RC2 MH089995).

Quality control and processing

All data analyses were conducted on the Dutch National Supercomputer Snellius. Quality control (QC) was executed using multiple tools, including Plink v1.9 (ref.[5]), Plink v2 (ref.[11]), and R v4.2.0, the GENESIS R-package[12], KING[11,13] and custom scripts in-house.

We first aligned our data using the HRC-1000G-check-bim.pl program v4.3.0 against the HRC reference panel (https://www.well.ox.ac.uk/~wrayner/tools/). In short, the software adjusts strand, position, and allele assignments as needed. SNPs are removed for inconsistencies such as mismatched alleles or significant frequency discrepancies with the HRC data. QC was conducted on two merged batches of cases and a single batch of controls before merging the datasets for subsequent analysis. A batch of cases with GRCh38 build was lifted to the positive strand of GRCh37 build prior to merging.

The initial quality control (QC) for genome-wide association study (GWAS) was executed in a multistep process, where first QC was performed in two separate case batches and in the control batch. This initial quality control was the same for all case/control batches: SNPs were excluded if duplicated, ambiguous (A/T or C/G), had missingness rates >2%, had Hardy-Weinberg equilibrium test P<10-6, or had minor allele frequency (MAF) <0.5%. Samples were excluded if missingness rates >5%. Will Rayner's tool was used to fix strand issues before merging, resulting in 427542 overlapping variants. 1022 cases and 7343 controls remained after initial QC and merging.

Then case and control batches were merged. After merging the case and control batches, we performed a second round of QC to prevent discrepancies in quality between batches. In particular, we restricted to variants found at high-quality in each of the batches, and filtered out variants with significant differential missingness between cases and controls (variants with p<0.01 were excluded). Furthermore, samples with missingness rates > 2% were removed.

For further sample QC procedures we also created a pruned set of genetic variants: The genotyped autosomal SNPs on each chromosome were pruned by the PLINK2 software in 2 iterations, where at first no SNP pairs with $r2 > 0.1$ were kept within any 250-SNP windows followed by a second iteration with $r2 > 0.2$ within any 50-SNP windows. KING was run on the pruned data to generate a genetic relationship matrix, which was used to remove duplicates and monozygotic twins. Samples with unresolved genotype-phenotype sex mismatches, or inbreeding coefficient $|F|>0.2$ were also excluded. This procedure left 978 high-quality DCM cases and 7207 controls.

Principal component analysis and ancestry inference were then conducted over the pruned variants, within the GENESIS R package[12] using PC-Relate and PC-Air. Ancestry labels (for continental super-populations) were assigned by overlaying samples (all cases and controls) over data from the 1000 Genomes Project[3]. We additionally defined a subset of homogeneous European ancestry individuals; in this subset cases and controls were kept if they were visual in-liers based on inspection of scatter plots of the first two principal components, leaving 783 DCM cases and 6978 controls of homogeneous European ancestry.

Following QC procedures, the genome-wide data (the non-pruned data) for the 978 high-quality cases and 7207 high-quality controls were imputed to the TOPMed imputation panel on the Michigan Imputation Server. Baseline characteristics for this cohort are presented in **Supplementary Table 3**.

GWAS

The genome-wide imputed data were put forward for GWAS, which was run using REGENIE v3.1.1 (ref.[14]) restricting to the subset of homogeneous European ancestry samples (783 cases, 6978 controls). Non-pruned high-quality variants from the genotyping array were used for null-model fitting in step 1. In both step 1 and 2, we adjusted for sex and PC1-10. An approximate

Firth's correction was used for variants reaching nominal $P<0.05$ in an initial test; standard errors were computed by back-correcting from the Firth's beta and Firth's P-value.

PRS analyses

As described in the main **Methods**, we performed PRS analyses in the Amsterdam cohort, by using summary statistics from GWAS-DCM and MTAG-DCM, after excluding the Amsterdam cohort from these GWAS and MTAG analyses. For all PRS analyses, we used the full set of 978 cases and 7207 controls (but subsetted in sensitivity analyses to European ancestry individuals). We first assessed the predictive capacity of GWAS-DCM and MTAG-DCM scores within i) all individuals, ii) individuals of European ancestry, iii) individuals not determined to be of European ancestry, iv) individuals with genetically-predicted male sex, v) individuals with genetically-determined female sex. In all logistic regression analyses, we adjusted for sex and PCs1-10. Given that MTAG-DCM showed the best predictive capacity in all settings (and with comparable results in the All of Us dataset) we focused on the MTAG-DCM PRS for all following analyses.

We then aimed to assess the cumulative contribution of common and rare genetic variation to clinical DCM. We therefore grouped DCM cases into 'rare genotype positive', 'rare genotype negative' and 'uncertain rare genotype', based on genetic testing findings. Of note, all probands underwent genetic testing at the department of clinical genetics at Amsterdam UMC; variant curations - including classifications into class 5 "pathogenic", class 4 "likely pathogenic", class 3 "variant of uncertain significance (VUS)", class 2 "likely benign" and class 1 "benign" - were performed as part of routine clinical care following current guidelines. We then grouped cases into 1) 'genotype positive' (N=193 cases) if they carried a class 4 or 5 variant in a high-confidence DCM gene (high-confidence DCM genes were those with a "strong" or "definitive" relationship with DCM based on ClinGen curation[15]; *TTN*, *LMNA*, *BAG3*, *RBM20*, *TNNC1*, *DSP*, *MYH7*, *FLNC*, *DSP*, *PLN*, *TNNT2*, *SCN5A*); 2) 'genotype negative' (N=294 cases) if they carried no class 3, 4

or 5 variants in any panel genes; and 3) 'uncertain genotype' in all other cases. We then assessed whether PRS was more strongly associated with 'genotype negative' or 'genotype positive' DCM, by performing logistic regression analyses where DCM cases were restricted only to either group (and comparing them to the same general control group). We also assessed whether PRS distributions differed between 'genotype positive' and 'genotype negative' DCM, by plotting PRS density plots for both groups. To identify statistical differences in PRS distribution between groups, we then performed linear regression analyses within those DCM cases, where we modeled PRS as the outcome, with a dummy variable for 'genotype positive' vs 'genotype negative' status as the predictor (adjusting for sex and PCs1-10).

FinnGen study description and methods

FinnGen, launched in 2017, is a public-private partnership research project that combines genotype data from newly collected and legacy samples administered by Finnish biobanks (https://www.finngen.fi/en) to provide novel insight into human diseases. This study includes genotype data from 453,733 individuals included in FinnGen Data Freeze 11. The data were linked by unique national personal identification numbers to the national hospital discharge registry (available from 1968), the cause of death registry (1969–) and the specialist outpatient registry (1998–).

Newly collected FinnGen samples were genotyped using a FinnGen ThermoFisher Axiom custom array (Thermo Fisher Scientific, San Diego, CA, USA), and legacy cohorts were genotyped using Illumina and Affymetrix arrays (Illumina Inc., San Diego, and Thermo Fisher Scientific, Santa Clara, CA, USA) as detailed previously[16]. Genotype quality control parameters are presented in **Supplementary Table 2** Principal component analysis was used to remove samples who were not of genotype-inferred Finnish ancestry similarly to the method described previously for Data Freeze 5 (ref.[16]). Genotype imputation was performed using a population-specific SISu v4 imputation reference panel comprised of 8,557 whole genomes based on the protocol available at: https://dx.doi.org/10.17504/protocols.io.xbgfijw.

We ran GWAS for NI-DCM and NICM, defined by ICD coding (I42.0 for NI-DCM, I42.0 and I50.1 for NICM). Prevalent and incident cases were combined. Cases were excluded from the analysis if they had antecedent codes for acute coronary syndrome (ICD-10 codes I21–I22 or the ICD-8/9 code 410 in hospital discharge or cause-of-death registries) and/or revascularization (NOSMESCO or Heart Patient procedural codes for coronary artery bypass grafting or coronary angioplasty), as previously described[17]; controls with codes for general heart failure were also removed from the analysis. Heart failure was defined using ICD codes from hospital discharge or

cause-of-death registries (ICD-10 codes I11.0, I13.0, I13.2, I50; ICD-9 codes 4029B, 428; ICD-8 codes 42700, 42710, 428 or 7824); KELA medication reimbursement number 201 (“Chronic Heart Failure”); or outpatient purchases of furosemide (ATC codes C03CA01 or C03EB01).

FinnGen GWAS were conducted using REGENIE v 2.2.4[14], with sex, age at death or end of follow-up, principal components 1–10, genotyping array, and genotyping batch as fixed-effect covariates. An approximate Firth’s correction was used for variants reaching nominal $P<0.05$ in an initial test; standard errors were computed by back-correcting from the Firth’s beta and Firth’s P-value.

FinnGen Ethics Statement

Study subjects in FinnGen provided informed consent for biobank research, based on the Finnish Biobank Act. Alternatively, separate research cohorts, collected prior the Finnish Biobank Act came into effect (in September 2013) and start of FinnGen (August 2017), were collected based on study-specific consents and later transferred to the Finnish biobanks after approval by Fimea (Finnish Medicines Agency), the National Supervisory Authority for Welfare and Health. Recruitment protocols followed the biobank protocols approved by Fimea. The Coordinating Ethics Committee of the Hospital District of Helsinki and Uusimaa (HUS) statement number for the FinnGen study is Nr HUS/990/2017.

The FinnGen study is approved by Finnish Institute for Health and Welfare (permit numbers: THL/2031/6.02.00/2017, THL/1101/5.05.00/2017, THL/341/6.02.00/2018, THL/2222/6.02.00/2018, THL/283/6.02.00/2019, THL/1721/5.05.00/2019 and THL/1524/5.05.00/2020), Digital and population data service agency (permit numbers: VRK43431/2017-3, VRK/6909/2018-3, VRK/4415/2019-3), the Social Insurance Institution (permit numbers: KELA 58/522/2017, KELA 131/522/2018, KELA 70/522/2019, KELA 98/522/2019, KELA 134/522/2019, KELA 138/522/2019, KELA 2/522/2020, KELA 16/522/2020), Findata permit numbers THL/2364/14.02/2020, THL/4055/14.06.00/2020, THL/3433/14.06.00/2020, THL/4432/14.06/2020, THL/5189/14.06/2020, THL/5894/14.06.00/2020, THL/6619/14.06.00/2020, THL/209/14.06.00/2021, THL/688/14.06.00/2021, THL/1284/14.06.00/2021, THL/1965/14.06.00/2021, THL/5546/14.02.00/2020, THL/2658/14.06.00/2021, THL/4235/14.06.00/2021, Statistics Finland (permit numbers: TK-53-1041-17 and TK/143/07.03.00/2020 (earlier TK-53-90-20) TK/1735/07.03.00/2021, TK/3112/07.03.00/2021) and Finnish Registry for Kidney Diseases permission/extract from the meeting minutes on 4th July 2019.

The Biobank Access Decisions for FinnGen samples and data utilized in FinnGen Data Freeze 11 include: THL Biobank BB2017_55, BB2017_111, BB2018_19, BB_2018_34, BB_2018_67, BB2018_71, BB2019_7, BB2019_8, BB2019_26, BB2020_1, BB2021_65, Finnish Red Cross Blood Service Biobank 7.12.2017, Helsinki Biobank HUS/359/2017, HUS/248/2020, HUS/430/2021 §28, §29, HUS/150/2022 §12, §13, §14, §15, §16, §17, §18, §23, §58 and §59, Auria Biobank AB17-5154 and amendment #1 (August 17 2020) and amendments BB_2021-0140, BB_2021-0156 (August 26 2021, Feb 2 2022), BB_2021-0169, BB_2021-0179, BB_2021-0161, AB20-5926 and amendment #1 (April 23 2020) and it´s modification (Sep 22 2021), BB_2022-0262, BB_2022-0256, Biobank Borealis of Northern Finland_2017_1013, 2021_5010, 2021_5018, 2021_5015, 2021_5015 Amendment, 2021_5023, 2021_5023 Amendment, 2021_5017, 2022_6001, 2022_6006 Amendment, BB22-0067, 2022_0262, Biobank of Eastern Finland 1186/2018 and amendment 22§/2020, 53§/2021, 13§/2022, 14§/2022, 15§/2022, 27§/2022, 28§/2022, 29§/2022, 33§/2022, 35§/2022, 36§/2022, 37§/2022, 39§/2022, 7§/2023, Finnish Clinical Biobank Tampere MH0004 and amendments (21.02.2020 & 06.10.2020), 8§/2021, 9§/2021, §9/2022, §10/2022, §12/2022, 13§/2022, §20/2022, §21/2022, §22/2022, §23/2022, 28§/2022, 29§/2022, 30§/2022, 31§/2022, 32§/2022, 38§/2022, 40§/2022, 42§/2022, 1§/2023, Central Finland Biobank 1-2017, BB_2021-0161, BB_2021-0169, BB_2021-0179, BB_2021-0170, BB_2022-0256, and Terveystalo Biobank STB 2018001 and amendment 25th Aug 2020, Finnish Hematological Registry and Clinical Biobank decision 18th June 2021, Arctic biobank P0844: ARC_2021_1001.

FinnGen Acknowledgements

We want to acknowledge the participants and investigators of FinnGen study. The FinnGen project is funded by two grants from Business Finland (HUS 4685/31/2016 and UH 4386/31/2016) and the following industry partners: AbbVie Inc., AstraZeneca UK Ltd, Biogen MA Inc., Bristol Myers Squibb (and Celgene Corporation & Celgene International II Sàrl), Genentech Inc., Merck Sharp & Dohme LCC, Pfizer Inc., GlaxoSmithKline Intellectual Property Development Ltd., Sanofi US Services Inc., Maze Therapeutics Inc., Janssen Biotech Inc, Novartis Pharma AG, and Boehringer Ingelheim International GmbH. Following biobanks are acknowledged for delivering biobank samples to FinnGen: Auria Biobank (www.auria.fi/biopankki), THL Biobank (www.thl.fi/biobank), Helsinki Biobank (www.helsinginbiopankki.fi), Biobank Borealis of Northern Finland (https://www.ppshp.fi/Tutkimus-ja-opetus/Biopankki/Pages/Biobank-Borealis-briefly-in-English.aspx), Finnish Clinical Biobank Tampere (www.tays.fi/en-US/Research_and_development/Finnish_Clinical_Biobank_Tampere), Biobank of Eastern Finland (www.ita-suomenbiopankki.fi/en), Central Finland Biobank (www.ksshp.fi/fi-FI/Potilaalle/Biopankki), Finnish Red Cross Blood Service Biobank (www.veripalvelu.fi/verenluovutus/biopankkitoiminta), Terveystalo Biobank (www.terveystalo.com/fi/Yritystietoa/Terveystalo-Biopankki/Biopankki/) and Arctic Biobank (https://www.oulu.fi/en/university/faculties-and-units/faculty-medicine/northern-finland-birth-cohorts-and-arctic-biobank). All Finnish Biobanks are members of BBMRI.fi infrastructure (www.bbmri.fi). Finnish Biobank Cooperative -FINBB (https://finbb.fi/) is the coordinator of BBMRI-ERIC operations in Finland. The Finnish biobank data can be accessed through the Fingenious® services (https://site.fingenious.fi/en/) managed by FINBB.

FinnGen banner authors

Full Name	Affiliation	Role 1	Role 2
Aarno Palotie	Institute for Molecular Medicine Finland (FIMM), HiLIFE, University of Helsinki, Helsinki, Finland; Broad Institute of MIT and Harvard; Massachusetts General Hospital	Steering Committee	Steering Committee
Mark Daly	Institute for Molecular Medicine Finland (FIMM), HiLIFE, University of Helsinki, Helsinki, Finland; Broad Institute of MIT and Harvard; Massachusetts General Hospital	Steering Committee	Steering Committee
Bridget Riley-Gills	Abbvie, Chicago, IL, United States	Steering Committee	Pharmaceutical companies
Howard Jacob	Abbvie, Chicago, IL, United States	Steering Committee	Pharmaceutical companies
Coralie Viollet	Astra Zeneca, Cambridge, United Kingdom	Steering Committee	Pharmaceutical companies
Slavé Petrovski	Astra Zeneca, Cambridge, United Kingdom	Steering Committee	Pharmaceutical companies
Chia-Yen Chen	Biogen, Cambridge, MA, United States	Steering Committee	Pharmaceutical companies
Sally John	Biogen, Cambridge, MA, United States	Steering Committee	Pharmaceutical companies
George Okafo	Boehringer Ingelheim, Ingelheim am Rhein, Germany	Steering Committee	Pharmaceutical companies
Robert Plenge	Bristol Myers Squibb, New York, NY, United States	Steering Committee	Pharmaceutical companies
Joseph Maranville	Bristol Myers Squibb, New York, NY, United States	Steering Committee	Pharmaceutical companies
Mark McCarthy	Genentech, San Francisco, CA, United States	Steering Committee	Pharmaceutical companies
Rion Pendergrass	Genentech, San Francisco, CA, United States	Steering Committee	Pharmaceutical companies
Jonathan Davitte	GlaxoSmithKline, Collegeville, PA, United States	Steering Committee	Pharmaceutical companies
Kirsi Auro	GlaxoSmithKline, Espoo, Finland	Steering Committee	Pharmaceutical companies
Simonne Longerich	Merck, Kenilworth, NJ, United States	Steering Committee	Pharmaceutical companies
Anders Mälarstig	Pfizer, New York, NY, United States	Steering Committee	Pharmaceutical companies
Anna Vlahiotis	Pfizer, New York, NY, United States	Steering Committee	Pharmaceutical companies
Katherine Klinger	Translational Sciences, Sanofi R&D, Framingham, MA, USA	Steering Committee	Pharmaceutical companies
Clement Chatelain	Translational Sciences, Sanofi R&D, Framingham, MA, USA	Steering Committee	Pharmaceutical companies
Jorg Blankenstein	Translational Sciences, Sanofi R&D, Framingham, MA, USA	Steering Committee	Pharmaceutical companies
Karol Estrada	Maze Therapeutics, San Francisco, CA, United States	Steering Committee	Pharmaceutical companies
Robert Graham	Maze Therapeutics, San Francisco, CA, United States	Steering Committee	Pharmaceutical companies
Dawn Waterworth	Janssen Research & Development, LLC, Spring House, PA, United States	Steering Committee	Pharmaceutical companies
Chris O´Donnell	Novartis Institutes for BioMedical Research, Cambridge, MA, United States	Steering Committee	Pharmaceutical companies
Nicole Renaud	Novartis Institutes for BioMedical Research, Cambridge, MA, United States	Steering Committee	Pharmaceutical companies

Tomi P. Mäkelä	HiLIFE, University of Helsinki, Finland, Finland	Steering Committee	**University of Helsinki & Biobanks**
Jaakko Kaprio	Institute for Molecular Medicine Finland (FIMM), HiLIFE, University of Helsinki, Helsinki, Finland	Steering Committee	**University of Helsinki & Biobanks**
Minna Ruddock	Arctic biobank / University of Oulu	Steering Committee	**University of Helsinki & Biobanks**
Petri Virolainen	Auria Biobank / University of Turku / Hospital District of Southwest Finland, Turku, Finland	Steering Committee	**University of Helsinki & Biobanks**
Antti Hakanen	Auria Biobank / University of Turku / Hospital District of Southwest Finland, Turku, Finland	Steering Committee	**University of Helsinki & Biobanks**
Terhi Kilpi	THL Biobank / Finnish Institute for Health and Welfare (THL), Helsinki, Finland	Steering Committee	**University of Helsinki & Biobanks**
Markus Perola	THL Biobank / Finnish Institute for Health and Welfare (THL), Helsinki, Finland	Steering Committee	**University of Helsinki & Biobanks**
Jukka Partanen	Finnish Red Cross Blood Service / Finnish Hematology Registry and Clinical Biobank, Helsinki, Finland	Steering Committee	**University of Helsinki & Biobanks**
Taneli Raivio	Helsinki Biobank / Helsinki University and Hospital District of Helsinki and Uusimaa, Helsinki	Steering Committee	**University of Helsinki & Biobanks**
Jani Tikkanen	Northern Finland Biobank Borealis / University of Oulu / Northern Ostrobothnia Hospital District, Oulu, Finland	Steering Committee	**University of Helsinki & Biobanks**
Raisa Serpi	Northern Finland Biobank Borealis / University of Oulu / Northern Ostrobothnia Hospital District, Oulu, Finland	Steering Committee	**University of Helsinki & Biobanks**
Kati Kristiansson	Finnish Clinical Biobank Tampere / University of Tampere / Pirkanmaa Hospital District, Tampere, Finland	Steering Committee	**University of Helsinki & Biobanks**
Veli-Matti Kosma	Biobank of Eastern Finland / University of Eastern Finland / Northern Savo Hospital District, Kuopio, Finland	Steering Committee	**University of Helsinki & Biobanks**
Jari Laukkanen	Central Finland Biobank / University of Jyväskylä / Central Finland Health Care District, Jyväskylä, Finland	Steering Committee	**University of Helsinki & Biobanks**
Marco Hautalahti	FINBB - Finnish biobank cooperative	Steering Committee	**University of Helsinki & Biobanks**
Outi Tuovila	Business Finland, Helsinki, Finland	Steering Committee	**Other Experts/ Non-Voting Members**
Jeffrey Waring	Abbvie, Chicago, IL, United States	Scientific Committee	**Pharmaceutical companies**
Bridget Riley-Gillis	Abbvie, Chicago, IL, United States	Scientific Committee	**Pharmaceutical companies**
Fedik Rahimov	Abbvie, Chicago, IL, United States	Scientific Committee	**Pharmaceutical companies**
Ioanna Tachmazidou	Astra Zeneca, Cambridge, United Kingdom	Scientific Committee	**Pharmaceutical companies**
Chia-Yen Chen	Biogen, Cambridge, MA, United States	Scientific Committee	**Pharmaceutical companies**
Zhihao Ding	Boehringer Ingelheim, Ingelheim am Rhein, Germany	Scientific Committee	**Pharmaceutical companies**
Marc Jung	Boehringer Ingelheim, Ingelheim am Rhein, Germany	Scientific Committee	**Pharmaceutical companies**
Hanati Tuoken	Boehringer Ingelheim, Ingelheim am Rhein, Germany	Scientific Committee	**Pharmaceutical companies**
Shameek Biswas	Bristol Myers Squibb, New York, NY, United States	Scientific Committee	**Pharmaceutical companies**

Rion Pendergrass	Genentech, San Francisco, CA, United States	Scientific Committee	**Pharmaceutical companies**
Jonathan Davitte	GlaxoSmithKline, Collegeville, PA, United States	Scientific Committee	**Pharmaceutical companies**
Neha Raghavan	Merck, Kenilworth, NJ, United States	Scientific Committee	**Pharmaceutical companies**
Adriana Huertas-Vazquez	Merck, Kenilworth, NJ, United States	Scientific Committee	**Pharmaceutical companies**
Jae-Hoon Sul	Merck, Kenilworth, NJ, United States	Scientific Committee	**Pharmaceutical companies**
Anders Mälarstig	Pfizer, New York, NY, United States	Scientific Committee	**Pharmaceutical companies**
Xinli Hu	Pfizer, New York, NY, United States	Scientific Committee	**Pharmaceutical companies**
Åsa Hedman	Pfizer, New York, NY, United States	Scientific Committee	**Pharmaceutical companies**
Katherine Klinger	Translational Sciences, Sanofi R&D, Framingham, MA, USA	Scientific Committee	**Pharmaceutical companies**
Robert Graham	Maze Therapeutics, San Francisco, CA, United States	Scientific Committee	**Pharmaceutical companies**
Dawn Waterworth	Janssen Research & Development, LLC, Spring House, PA, United States	Scientific Committee	**Pharmaceutical companies**
Nicole Renaud	Novartis Institutes for BioMedical Research, Cambridge, MA, United States	Scientific Committee	**Pharmaceutical companies**
Ma´en Obeidat	Novartis Institutes for BioMedical Research, Cambridge, MA, United States	Scientific Committee	**Pharmaceutical companies**
Jonathan Chung	Novartis Institutes for BioMedical Research, Cambridge, MA, United States	Scientific Committee	**Pharmaceutical companies**
Jonas Zierer	Novartis Institutes for BioMedical Research, Cambridge, MA, United States	Scientific Committee	**Pharmaceutical companies**
Mari Niemi	Novartis Institutes for BioMedical Research, Cambridge, MA, United States	Scientific Committee	**Pharmaceutical companies**
Samuli Ripatti	Institute for Molecular Medicine Finland (FIMM), HiLIFE, University of Helsinki, Helsinki, Finland	Scientific Committee	**University of Helsinki & Biobanks**
Johanna Schleutker	Auria Biobank / Univ. of Turku / Hospital District of Southwest Finland, Turku, Finland	Scientific Committee	**University of Helsinki & Biobanks**
Markus Perola	THL Biobank / Finnish Institute for Health and Welfare (THL), Helsinki, Finland	Scientific Committee	**University of Helsinki & Biobanks**
Mikko Arvas	Finnish Red Cross Blood Service / Finnish Hematology Registry and Clinical Biobank, Helsinki, Finland	Scientific Committee	**University of Helsinki & Biobanks**
Olli Carpén	Helsinki Biobank / Helsinki University and Hospital District of Helsinki and Uusimaa, Helsinki	Scientific Committee	**University of Helsinki & Biobanks**
Reetta Hinttala	Northern Finland Biobank Borealis / University of Oulu / Northern Ostrobothnia Hospital District, Oulu, Finland	Scientific Committee	**University of Helsinki & Biobanks**
Johannes Kettunen	Northern Finland Biobank Borealis / University of Oulu / Northern Ostrobothnia Hospital District, Oulu, Finland	Scientific Committee	**University of Helsinki & Biobanks**
Arto Mannermaa	Biobank of Eastern Finland / University of Eastern Finland / Northern Savo Hospital District, Kuopio, Finland	Scientific Committee	**University of Helsinki & Biobanks**
Katriina Aalto-Setälä	Faculty of Medicine and Health Technology, Tampere University, Tampere, Finland	Scientific Committee	**University of Helsinki & Biobanks**
Mika Kähönen	Finnish Clinical Biobank Tampere / University of Tampere / Pirkanmaa Hospital District, Tampere, Finland	Scientific Committee	**University of Helsinki & Biobanks**
Jari Laukkanen	Central Finland Biobank / University of Jyväskylä / Central Finland Health Care District, Jyväskylä, Finland	Scientific Committee	**University of Helsinki & Biobanks**

Johanna Mäkelä	FINBB - Finnish biobank cooperative	Scientific Committee	**University of Helsinki & Biobanks**
Reetta Kälviäinen	Northern Savo Hospital District, Kuopio, Finland	Clinical Groups	**Neurology Group**
Valtteri Julkunen	Northern Savo Hospital District, Kuopio, Finland	Clinical Groups	**Neurology Group**
Hilkka Soininen	Northern Savo Hospital District, Kuopio, Finland	Clinical Groups	**Neurology Group**
Anne Remes	Northern Ostrobothnia Hospital District, Oulu, Finland	Clinical Groups	**Neurology Group**
Mikko Hiltunen	University of Eastern Finland, Kuopio, Finland	Clinical Groups	**Neurology Group**
Jukka Peltola	Pirkanmaa Hospital District, Tampere, Finland	Clinical Groups	**Neurology Group**
Minna Raivio	Hospital District of Helsinki and Uusimaa, Helsinki, Finland	Clinical Groups	**Neurology Group**
Pentti Tienari	Hospital District of Helsinki and Uusimaa, Helsinki, Finland	Clinical Groups	**Neurology Group**
Juha Rinne	Hospital District of Southwest Finland, Turku, Finland	Clinical Groups	**Neurology Group**
Roosa Kallionpää	Hospital District of Southwest Finland, Turku, Finland	Clinical Groups	**Neurology Group**
Juulia Partanen	Institute for Molecular Medicine Finland, HiLIFE, University of Helsinki, Finland	Clinical Groups	**Neurology Group**
Adam Ziemann	Abbvie, Chicago, IL, United States	Clinical Groups	**Neurology Group**
Nizar Smaoui	Abbvie, Chicago, IL, United States	Clinical Groups	**Neurology Group**
Anne Lehtonen	Abbvie, Chicago, IL, United States	Clinical Groups	**Neurology Group**
Susan Eaton	Biogen, Cambridge, MA, United States	Clinical Groups	**Neurology Group**
Heiko Runz	Biogen, Cambridge, MA, United States	Clinical Groups	**Neurology Group**
Sanni Lahdenperä	Biogen, Cambridge, MA, United States	Clinical Groups	**Neurology Group**
Shameek Biswas	Bristol Myers Squibb, New York, NY, United States	Clinical Groups	**Neurology Group**
Natalie Bowers	Genentech, San Francisco, CA, United States	Clinical Groups	**Neurology Group**
Edmond Teng	Genentech, San Francisco, CA, United States	Clinical Groups	**Neurology Group**
Rion Pendergrass	Genentech, San Francisco, CA, United States	Clinical Groups	**Neurology Group**
Fanli Xu	GlaxoSmithKline, Brentford, United Kingdom	Clinical Groups	**Neurology Group**
David Pulford	GlaxoSmithKline, Stevenage, United Kingdom	Clinical Groups	**Neurology Group**
Kirsi Auro	GlaxoSmithKline, Espoo, Finland	Clinical Groups	**Neurology Group**
Laura Addis	GlaxoSmithKline, Brentford, United Kingdom	Clinical Groups	**Neurology Group**
John Eicher	GlaxoSmithKline, Brentford, United Kingdom	Clinical Groups	**Neurology Group**
Qingqin S Li	Janssen Research & Development, LLC, Titusville, NJ 08560, United States	Clinical Groups	**Neurology Group**
Karen He	Janssen Research & Development, LLC, Spring House, PA, United States	Clinical Groups	**Neurology Group**
Ekaterina Khramtsova	Janssen Research & Development, LLC, Spring House, PA, United States	Clinical Groups	**Neurology Group**
Neha Raghavan	Merck, Kenilworth, NJ, United States	Clinical Groups	**Neurology Group**
Martti Färkkilä	Hospital District of Helsinki and Uusimaa, Helsinki, Finland	Clinical Groups	**Gastroenterology Group**
Jukka Koskela	Hospital District of Helsinki and Uusimaa, Helsinki, Finland	Clinical Groups	**Gastroenterology Group**
Sampsa Pikkarainen	Hospital District of Helsinki and Uusimaa, Helsinki, Finland	Clinical Groups	**Gastroenterology Group**
Airi Jussila	Pirkanmaa Hospital District, Tampere, Finland	Clinical Groups	**Gastroenterology Group**
Katri Kaukinen	Pirkanmaa Hospital District, Tampere, Finland	Clinical Groups	**Gastroenterology Group**

Timo Blomster	Northern Ostrobothnia Hospital District, Oulu, Finland	Clinical Groups	**Gastroenterology Group**
Mikko Kiviniemi	Northern Savo Hospital District, Kuopio, Finland	Clinical Groups	**Gastroenterology Group**
Markku Voutilainen	Hospital District of Southwest Finland, Turku, Finland	Clinical Groups	**Gastroenterology Group**
Mark Daly	Institute for Molecular Medicine, Finland (FIMM), HiLIFE, University of Helsinki, Helsinki, Finland; Broad Institute of MIT and Harvard; Massachusetts General Hospital	Clinical Groups	**Gastroenterology Group**
Jeffrey Waring	Abbvie, Chicago, IL, United States	Clinical Groups	**Gastroenterology Group**
Nizar Smaoui	Abbvie, Chicago, IL, United States	Clinical Groups	**Gastroenterology Group**
Fedik Rahimov	Abbvie, Chicago, IL, United States	Clinical Groups	**Gastroenterology Group**
Anne Lehtonen	Abbvie, Chicago, IL, United States	Clinical Groups	**Gastroenterology Group**
Tim Lu	Genentech, San Francisco, CA, United States	Clinical Groups	**Gastroenterology Group**
Natalie Bowers	Genentech, San Francisco, CA, United States	Clinical Groups	**Gastroenterology Group**
Rion Pendergrass	Genentech, San Francisco, CA, United States	Clinical Groups	**Gastroenterology Group**
Linda McCarthy	GlaxoSmithKline, Brentford, United Kingdom	Clinical Groups	**Gastroenterology Group**
Amy Hart	Janssen Research & Development, LLC, Spring House, PA, United States	Clinical Groups	**Gastroenterology Group**
Meijian Guan	Janssen Research & Development, LLC, Spring House, PA, United States	Clinical Groups	**Gastroenterology Group**
Jason Miller	Merck, Kenilworth, NJ, United States	Clinical Groups	**Gastroenterology Group**
Kirsi Kalpala	Pfizer, New York, NY, United States	Clinical Groups	**Gastroenterology Group**
Melissa Miller	Pfizer, New York, NY, United States	Clinical Groups	**Gastroenterology Group**
Xinli Hu	Pfizer, New York, NY, United States	Clinical Groups	**Gastroenterology Group**
Kari Eklund	Hospital District of Helsinki and Uusimaa, Helsinki, Finland	Clinical Groups	**Rheumatology Group**
Antti Palomäki	Hospital District of Southwest Finland, Turku, Finland	Clinical Groups	**Rheumatology Group**
Pia Isomäki	Pirkanmaa Hospital District, Tampere, Finland	Clinical Groups	**Rheumatology Group**
Laura Pirilä	Hospital District of Southwest Finland, Turku, Finland	Clinical Groups	**Rheumatology Group**
Oili Kaipiainen-Seppänen	Northern Savo Hospital District, Kuopio, Finland	Clinical Groups	**Rheumatology Group**
Johanna Huhtakangas	Northern Ostrobothnia Hospital District, Oulu, Finland	Clinical Groups	**Rheumatology Group**
Nina Mars	Institute for Molecular Medicine Finland (FIMM), HiLIFE, University of Helsinki, Helsinki, Finland	Clinical Groups	**Rheumatology Group**
Jeffrey Waring	Abbvie, Chicago, IL, United States	Clinical Groups	**Rheumatology Group**
Fedik Rahimov	Abbvie, Chicago, IL, United States	Clinical Groups	**Rheumatology Group**
Apinya Lertratanakul	Abbvie, Chicago, IL, United States	Clinical Groups	**Rheumatology Group**
Nizar Smaoui	Abbvie, Chicago, IL, United States	Clinical Groups	**Rheumatology Group**
Anne Lehtonen	Abbvie, Chicago, IL, United States	Clinical Groups	**Rheumatology Group**
Coralie Viollet	AstraZeneca, Cambridge, United Kingdom	Clinical Groups	**Rheumatology Group**
Marla Hochfeld	Bristol Myers Squibb, New York, NY, United States	Clinical Groups	**Rheumatology Group**
Natalie Bowers	Genentech, San Francisco, CA, United States	Clinical Groups	**Rheumatology Group**
Rion Pendergrass	Genentech, San Francisco, CA, United States	Clinical Groups	**Rheumatology Group**
Jorge Esparza Gordillo	GlaxoSmithKline, Brentford, United Kingdom	Clinical Groups	**Rheumatology Group**

Kirsi Auro	GlaxoSmithKline, Espoo, Finland	Clinical Groups	**Rheumatology Group**
Dawn Waterworth	Janssen Research & Development, LLC, Spring House, PA, United States	Clinical Groups	**Rheumatology Group**
Fabiana Farias	Merck, Kenilworth, NJ, United States	Clinical Groups	**Rheumatology Group**
Kirsi Kalpala	Pfizer, New York, NY, United States	Clinical Groups	**Rheumatology Group**
Nan Bing	Pfizer, New York, NY, United States	Clinical Groups	**Rheumatology Group**
Xinli Hu	Pfizer, New York, NY, United States	Clinical Groups	**Rheumatology Group**
Tarja Laitinen	Pirkanmaa Hospital District, Tampere, Finland	Clinical Groups	**Pulmonology Group**
Margit Pelkonen	Northern Savo Hospital District, Kuopio, Finland	Clinical Groups	**Pulmonology Group**
Paula Kauppi	Hospital District of Helsinki and Uusimaa, Helsinki, Finland	Clinical Groups	**Pulmonology Group**
Hannu Kankaanranta	University of Gothenburg, Gothenburg, Sweden/ Seinäjoki Central Hospital, Seinäjoki, Finland/ Tampere University, Tampere, Finland	Clinical Groups	**Pulmonology Group**
Terttu Harju	Northern Ostrobothnia Hospital District, Oulu, Finland	Clinical Groups	**Pulmonology Group**
Riitta Lahesmaa	Hospital District of Southwest Finland, Turku, Finland	Clinical Groups	**Pulmonology Group**
Nizar Smaoui	Abbvie, Chicago, IL, United States	Clinical Groups	**Pulmonology Group**
Coralie Viollet	AstraZeneca, Cambridge, United Kingdom	Clinical Groups	**Pulmonology Group**
Susan Eaton	Biogen, Cambridge, MA, United States	Clinical Groups	**Pulmonology Group**
Hubert Chen	Genentech, San Francisco, CA, United States	Clinical Groups	**Pulmonology Group**
Rion Pendergrass	Genentech, San Francisco, CA, United States	Clinical Groups	**Pulmonology Group**
Natalie Bowers	Genentech, San Francisco, CA, United States	Clinical Groups	**Pulmonology Group**
Joanna Betts	GlaxoSmithKline, Brentford, United Kingdom	Clinical Groups	**Pulmonology Group**
Kirsi Auro	GlaxoSmithKline, Espoo, Finland	Clinical Groups	**Pulmonology Group**
Rajashree Mishra	GlaxoSmithKline, Brentford, United Kingdom	Clinical Groups	**Pulmonology Group**
Majd Mouded	Novartis, Basel, Switzerland	Clinical Groups	**Pulmonology Group**
Debby Ngo	Novartis, Basel, Switzerland	Clinical Groups	**Pulmonology Group**
Teemu Niiranen	Finnish Institute for Health and Welfare (THL), Helsinki, Finland	Clinical Groups	**Cardiometabolic Diseases Group**
Felix Vaura	Finnish Institute for Health and Welfare (THL), Helsinki, Finland	Clinical Groups	**Cardiometabolic Diseases Group**
Veikko Salomaa	Finnish Institute for Health and Welfare (THL), Helsinki, Finland	Clinical Groups	**Cardiometabolic Diseases Group**
Kaj Metsärinne	Hospital District of Southwest Finland, Turku, Finland	Clinical Groups	**Cardiometabolic Diseases Group**
Jenni Aittokallio	Hospital District of Southwest Finland, Turku, Finland	Clinical Groups	**Cardiometabolic Diseases Group**
Mika Kähönen	Pirkanmaa Hospital District, Tampere, Finland	Clinical Groups	**Cardiometabolic Diseases Group**
Jussi Hernesniemi	Pirkanmaa Hospital District, Tampere, Finland	Clinical Groups	**Cardiometabolic Diseases Group**
Daniel Gordin	Hospital District of Helsinki and Uusimaa, Helsinki, Finland	Clinical Groups	**Cardiometabolic Diseases Group**
Juha Sinisalo	Hospital District of Helsinki and Uusimaa, Helsinki, Finland	Clinical Groups	**Cardiometabolic Diseases Group**
Marja-Riitta Taskinen	Hospital District of Helsinki and Uusimaa, Helsinki, Finland	Clinical Groups	**Cardiometabolic Diseases Group**
Tiinamaija Tuomi	Hospital District of Helsinki and Uusimaa, Helsinki, Finland	Clinical Groups	**Cardiometabolic Diseases Group**

Timo Hiltunen	Hospital District of Helsinki and Uusimaa, Helsinki, Finland	Clinical Groups	**Cardiometabolic Diseases Group**
Jari Laukkanen	Central Finland Health Care District, Jyväskylä, Finland	Clinical Groups	**Cardiometabolic Diseases Group**
Amanda Elliott	Institute for Molecular Medicine Finland (FIMM), HiLIFE, University of Helsinki, Helsinki, Finland; Broad Institute, Cambridge, MA, USA and Massachusetts General Hospital, Boston, MA, USA	Clinical Groups	**Cardiometabolic Diseases Group**
Mary Pat Reeve	Institute for Molecular Medicine Finland (FIMM), HiLIFE, University of Helsinki, Helsinki, Finland	Clinical Groups	**Cardiometabolic Diseases Group**
Sanni Ruotsalainen	Institute for Molecular Medicine Finland (FIMM), HiLIFE, University of Helsinki, Helsinki, Finland	Clinical Groups	**Cardiometabolic Diseases Group**
Dirk Paul	Astra Zeneca, Cambridge, United Kingdom	Clinical Groups	**Cardiometabolic Diseases Group**
Natalie Bowers	Genentech, San Francisco, CA, United States	Clinical Groups	**Cardiometabolic Diseases Group**
Rion Pendergrass	Genentech, San Francisco, CA, United States	Clinical Groups	**Cardiometabolic Diseases Group**
Audrey Chu	GlaxoSmithKline, Brentford, United Kingdom	Clinical Groups	**Cardiometabolic Diseases Group**
Kirsi Auro	GlaxoSmithKline, Espoo, Finland	Clinical Groups	**Cardiometabolic Diseases Group**
Dermot Reilly	Janssen Research & Development, LLC, Boston, MA, United States	Clinical Groups	**Cardiometabolic Diseases Group**
Mike Mendelson	Novartis, Boston, MA, United States	Clinical Groups	**Cardiometabolic Diseases Group**
Jaakko Parkkinen	Pfizer, New York, NY, United States	Clinical Groups	**Cardiometabolic Diseases Group**
Melissa Miller	Pfizer, New York, NY, United States	Clinical Groups	**Cardiometabolic Diseases Group**
Tuomo Meretoja	Department of Breast Surgery, Helsinki University Hospital Comprehensive Cancer Center and University of Helsinki, Helsinki, Finland	Clinical Groups	**Oncology Group**
Heikki Joensuu	Department of Oncology, Helsinki University Hospital Comprehensive Cancer Center and University of Helsinki, Helsinki, Finland	Clinical Groups	**Oncology Group**
Olli Carpén	Hospital District of Helsinki and Uusimaa, Helsinki, Finland	Clinical Groups	**Oncology Group**
Johanna Mattson	Hospital District of Helsinki and Uusimaa, Helsinki, Finland	Clinical Groups	**Oncology Group**
Eveliina Salminen	Hospital District of Helsinki and Uusimaa, Helsinki, Finland	Clinical Groups	**Oncology Group**
Annika Auranen	Pirkanmaa Hospital District , Tampere, Finland	Clinical Groups	**Oncology Group**
Peeter Karihtala	Department of Oncology, Helsinki University Hospital Comprehensive Cancer Center and University of Helsinki, Helsinki, Finland	Clinical Groups	**Oncology Group**
Päivi Auvinen	Northern Savo Hospital District, Kuopio, Finland	Clinical Groups	**Oncology Group**
Klaus Elenius	Hospital District of Southwest Finland, Turku, Finland	Clinical Groups	**Oncology Group**
Johanna Schleutker	Hospital District of Southwest Finland, Turku, Finland	Clinical Groups	**Oncology Group**
Esa Pitkänen	Institute for Molecular Medicine Finland (FIMM), HiLIFE, University of Helsinki, Helsinki, Finland	Clinical Groups	**Oncology Group**

Nina Mars	Institute for Molecular Medicine Finland (FIMM), HiLIFE, University of Helsinki, Helsinki, Finland	Clinical Groups	**Oncology Group**
Mark Daly	Institute for Molecular Medicine Finland (FIMM), HiLIFE, University of Helsinki, Helsinki, Finland; Broad Institute of MIT and Harvard; Massachusetts General Hospital	Clinical Groups	**Oncology Group**
Relja Popovic	Abbvie, Chicago, IL, United States	Clinical Groups	**Oncology Group**
Jeffrey Waring	Abbvie, Chicago, IL, United States	Clinical Groups	**Oncology Group**
Bridget Riley-Gillis	Abbvie, Chicago, IL, United States	Clinical Groups	**Oncology Group**
Anne Lehtonen	Abbvie, Chicago, IL, United States	Clinical Groups	**Oncology Group**
Margarete Fabre	AstraZeneca, Cambridge, United Kingdom	Clinical Groups	**Oncology Group**
Jennifer Schutzman	Genentech, San Francisco, CA, United States	Clinical Groups	**Oncology Group**
Natalie Bowers	Genentech, San Francisco, CA, United States	Clinical Groups	**Oncology Group**
Rion Pendergrass	Genentech, San Francisco, CA, United States	Clinical Groups	**Oncology Group**
Diptee Kulkarni	GlaxoSmithKline, Brentford, United Kingdom	Clinical Groups	**Oncology Group**
Kirsi Auro	GlaxoSmithKline, Espoo, Finland	Clinical Groups	**Oncology Group**
Alessandro Porello	Janssen Research & Development, LLC, Spring House, PA, United States	Clinical Groups	**Oncology Group**
Andrey Loboda	Merck, Kenilworth, NJ, United States	Clinical Groups	**Oncology Group**
Heli Lehtonen	Pfizer, New York, NY, United States	Clinical Groups	**Oncology Group**
Stefan McDonough	Pfizer, New York, NY, United States	Clinical Groups	**Oncology Group**
Sauli Vuoti	Janssen-Cilag Oy, Espoo, Finland	Clinical Groups	**Oncology Group**
Kai Kaarniranta	Northern Savo Hospital District, Kuopio, Finland; Department of Molecular Genetics, University of Lodz, Lodz, Poland	Clinical Groups	**Opthalmology Group**
Joni A Turunen	Helsinki University Hospital and University of Helsinki, Helsinki, Finland; Eye Genetics Group, Folkhälsan Research Center, Helsinki, Finland	Clinical Groups	**Opthalmology Group**
Terhi Ollila	Hospital District of Helsinki and Uusimaa, Helsinki, Finland	Clinical Groups	**Opthalmology Group**
Hannu Uusitalo	Pirkanmaa Hospital District, Tampere, Finland	Clinical Groups	**Opthalmology Group**
Juha Karjalainen	Institute for Molecular Medicine Finland (FIMM), HiLIFE, University of Helsinki, Helsinki, Finland	Clinical Groups	**Opthalmology Group**
Esa Pitkänen	Institute for Molecular Medicine Finland (FIMM), HiLIFE, University of Helsinki, Helsinki, Finland	Clinical Groups	**Opthalmology Group**
Mengzhen Liu	Abbvie, Chicago, IL, United States	Clinical Groups	**Opthalmology Group**
Heiko Runz	Biogen, Cambridge, MA, United States	Clinical Groups	**Opthalmology Group**
Stephanie Loomis	Biogen, Cambridge, MA, United States	Clinical Groups	**Opthalmology Group**
Erich Strauss	Genentech, San Francisco, CA, United States	Clinical Groups	**Opthalmology Group**
Natalie Bowers	Genentech, San Francisco, CA, United States	Clinical Groups	**Opthalmology Group**
Hao Chen	Genentech, San Francisco, CA, United States	Clinical Groups	**Opthalmology Group**
Rion Pendergrass	Genentech, San Francisco, CA, United States	Clinical Groups	**Opthalmology Group**
Kaisa Tasanen	Northern Ostrobothnia Hospital District, Oulu, Finland	Clinical Groups	**Dermatology Group**
Laura Huilaja	Northern Ostrobothnia Hospital District, Oulu, Finland	Clinical Groups	**Dermatology Group**
Katariina Hannula-Jouppi	Hospital District of Helsinki and Uusimaa, Helsinki, Finland	Clinical Groups	**Dermatology Group**
Teea Salmi	Pirkanmaa Hospital District, Tampere, Finland	Clinical Groups	**Dermatology Group**
Sirkku Peltonen	Hospital District of Southwest Finland, Turku, Finland	Clinical Groups	**Dermatology Group**

Leena Koulu	Hospital District of Southwest Finland, Turku, Finland	Clinical Groups	**Dermatology Group**
Nizar Smaoui	Abbvie, Chicago, IL, United States	Clinical Groups	**Dermatology Group**
Fedik Rahimov	Abbvie, Chicago, IL, United States	Clinical Groups	**Dermatology Group**
Anne Lehtonen	Abbvie, Chicago, IL, United States	Clinical Groups	**Dermatology Group**
David Choy	Genentech, San Francisco, CA, United States	Clinical Groups	**Dermatology Group**
Rion Pendergrass	Genentech, San Francisco, CA, United States	Clinical Groups	**Dermatology Group**
Dawn Waterworth	Janssen Research & Development, LLC, Spring House, PA, United States	Clinical Groups	**Dermatology Group**
Kirsi Kalpala	Pfizer, New York, NY, United States	Clinical Groups	**Dermatology Group**
Ying Wu	Pfizer, New York, NY, United States	Clinical Groups	**Dermatology Group**
Pirkko Pussinen	Hospital District of Helsinki and Uusimaa, Helsinki, Finland	Clinical Groups	**Odontology Group**
Aino Salminen	Hospital District of Helsinki and Uusimaa, Helsinki, Finland	Clinical Groups	**Odontology Group**
Tuula Salo	Hospital District of Helsinki and Uusimaa, Helsinki, Finland	Clinical Groups	**Odontology Group**
David Rice	Hospital District of Helsinki and Uusimaa, Helsinki, Finland	Clinical Groups	**Odontology Group**
Pekka Nieminen	Hospital District of Helsinki and Uusimaa, Helsinki, Finland	Clinical Groups	**Odontology Group**
Ulla Palotie	Hospital District of Helsinki and Uusimaa, Helsinki, Finland	Clinical Groups	**Odontology Group**
Maria Siponen	Northern Savo Hospital District, Kuopio, Finland	Clinical Groups	**Odontology Group**
Liisa Suominen	Northern Savo Hospital District, Kuopio, Finland	Clinical Groups	**Odontology Group**
Päivi Mäntylä	Northern Savo Hospital District, Kuopio, Finland	Clinical Groups	**Odontology Group**
Ulvi Gursoy	Hospital District of Southwest Finland, Turku, Finland	Clinical Groups	**Odontology Group**
Vuokko Anttonen	Northern Ostrobothnia Hospital District, Oulu, Finland	Clinical Groups	**Odontology Group**
Kirsi Sipilä	Research Unit of Oral Health Sciences Faculty of Medicine, University of Oulu, Oulu, Finland; Medical Research Center, Oulu, Oulu University Hospital and University of Oulu, Oulu, Finland	Clinical Groups	**Odontology Group**
Rion Pendergrass	Genentech, San Francisco, CA, United States	Clinical Groups	**Odontology Group**
Hannele Laivuori	Institute for Molecular Medicine Finland (FIMM), HiLIFE, University of Helsinki, Helsinki, Finland	Clinical Groups	**Women's Health and Reproduction Group**
Venla Kurra	Pirkanmaa Hospital District, Tampere, Finland	Clinical Groups	**Women's Health and Reproduction Group**
Laura Kotaniemi-Talonen	Pirkanmaa Hospital District, Tampere, Finland	Clinical Groups	**Women's Health and Reproduction Group**
Oskari Heikinheimo	Hospital District of Helsinki and Uusimaa, Helsinki, Finland	Clinical Groups	**Women's Health and Reproduction Group**
Ilkka Kalliala	Hospital District of Helsinki and Uusimaa, Helsinki, Finland	Clinical Groups	**Women's Health and Reproduction Group**
Lauri Aaltonen	Hospital District of Helsinki and Uusimaa, Helsinki, Finland	Clinical Groups	**Women's Health and Reproduction Group**
Varpu Jokimaa	Hospital District of Southwest Finland, Turku, Finland	Clinical Groups	**Women's Health and Reproduction Group**
Johannes Kettunen	Northern Ostrobothnia Hospital District, Oulu, Finland	Clinical Groups	**Women's Health and Reproduction Group**
Marja Vääräsmäki	Northern Ostrobothnia Hospital District, Oulu, Finland	Clinical Groups	**Women's Health and Reproduction Group**

Outi Uimari	Northern Ostrobothnia Hospital District, Oulu, Finland	Clinical Groups	**Women’s Health and Reproduction Group**
Laure Morin-Papunen	Northern Ostrobothnia Hospital District, Oulu, Finland	Clinical Groups	**Women’s Health and Reproduction Group**
Maarit Niinimäki	Northern Ostrobothnia Hospital District, Oulu, Finland	Clinical Groups	**Women’s Health and Reproduction Group**
Terhi Piltonen	Northern Ostrobothnia Hospital District, Oulu, Finland	Clinical Groups	**Women’s Health and Reproduction Group**
Katja Kivinen	Institute for Molecular Medicine Finland (FIMM), HiLIFE, University of Helsinki, Helsinki, Finland	Clinical Groups	**Women’s Health and Reproduction Group**
Elisabeth Widen	Institute for Molecular Medicine Finland (FIMM), HiLIFE, University of Helsinki, Helsinki, Finland	Clinical Groups	**Women’s Health and Reproduction Group**
Taru Tukiainen	Institute for Molecular Medicine Finland (FIMM), HiLIFE, University of Helsinki, Helsinki, Finland	Clinical Groups	**Women’s Health and Reproduction Group**
Mary Pat Reeve	Institute for Molecular Medicine Finland (FIMM), HiLIFE, University of Helsinki, Helsinki, Finland	Clinical Groups	**Women’s Health and Reproduction Group**
Mark Daly	Institute for Molecular Medicine Finland (FIMM), HiLIFE, University of Helsinki, Helsinki, Finland; Broad Institute of MIT and Harvard; Massachusetts General Hospital	Clinical Groups	**Women’s Health and Reproduction Group**
Niko Välimäki	University of Helsinki, Helsinki, Finland	Clinical Groups	**Women’s Health and Reproduction Group**
Eija Laakkonen	University of Jyväskylä, Jyväskylä, Finland	Clinical Groups	**Women’s Health and Reproduction Group**
Jaakko Tyrmi	University of Oulu, Oulu, Finland / University of Tampere, Tampere, Finland	Clinical Groups	**Women’s Health and Reproduction Group**
Heidi Silven	University of Oulu, Oulu, Finland	Clinical Groups	**Women’s Health and Reproduction Group**
Eeva Sliz	University of Oulu, Oulu, Finland	Clinical Groups	**Women’s Health and Reproduction Group**
Riikka Arffman	University of Oulu, Oulu, Finland	Clinical Groups	**Women’s Health and Reproduction Group**
Susanna Savukoski	University of Oulu, Oulu, Finland	Clinical Groups	**Women’s Health and Reproduction Group**
Triin Laisk	Estonian biobank, Tartu, Estonia	Clinical Groups	**Women’s Health and Reproduction Group**
Natalia Pujol	Estonian biobank, Tartu, Estonia	Clinical Groups	**Women’s Health and Reproduction Group**
Mengzhen Liu	Abbvie, Chicago, IL, United States	Clinical Groups	**Women’s Health and Reproduction Group**
Bridget Riley-Gillis	Abbvie, Chicago, IL, United States	Clinical Groups	**Women’s Health and Reproduction Group**
Rion Pendergrass	Genentech, San Francisco, CA, United States	Clinical Groups	**Women’s Health and Reproduction Group**
Janet Kumar	GlaxoSmithKline, Collegeville, PA, United States	Clinical Groups	**Women’s Health and Reproduction Group**
Kirsi Auro	GlaxoSmithKline, Espoo, Finland	Clinical Groups	**Women’s Health and Reproduction Group**
Iiris Hovatta	University of Helsinki, Finland	Clinical Groups	**Depression group**
Chia-Yen Chen	Biogen, Cambridge, MA, United States	Clinical Groups	**Depression group**
Erkki Isometsä	Hospital District of Helsinki and Uusimaa, Helsinki, Finland	Clinical Groups	**Depression group**
Hanna Ollila	Institute for Molecular Medicine Finland (FIMM), HiLIFE, University of Helsinki, Helsinki, Finland	Clinical Groups	**Depression group**

Jaana Suvisaari	Finnish Institute for Health and Welfare (THL), Helsinki, Finland	Clinical Groups	**Depression group**
Antti Mäkitie	Department of Otorhinolaryngology - Head and Neck Surgery, University of Helsinki and Helsinki University Hospital, Helsinki, Finland	Clinical Groups	**ENT (ear, nose and throath) Group**
Argyro Bizaki-Vallaskangas	Pirkanmaa Hospital District, Tampere, Finland	Clinical Groups	**ENT (ear, nose and throath) Group**
Sanna Toppila-Salmi	University of Eastern Finland and Kuopio University Hospital, Department of Otorhinolaryngology, Kuopio, Finland and Department of Allergy, Helsinki University Hospital and University of Helsinki, Finland	Clinical Groups	**ENT (ear, nose and throath) Group**
Tytti Willberg	Hospital District of Southwest Finland, Turku, Finland	Clinical Groups	**ENT (ear, nose and throath) Group**
Elmo Saarentaus	Institute for Molecular Medicine Finland (FIMM), HiLIFE, University of Helsinki, Helsinki, Finland	Clinical Groups	**ENT (ear, nose and throath) Group**
Antti Aarnisalo	Hospital District of Helsinki and Uusimaa, Helsinki, Finland	Clinical Groups	**ENT (ear, nose and throath) Group**
Eveliina Salminen	Hospital District of Helsinki and Uusimaa, Helsinki, Finland	Clinical Groups	**ENT (ear, nose and throath) Group**
Elisa Rahikkala	Northern Ostrobothnia Hospital District, Oulu, Finland	Clinical Groups	**ENT (ear, nose and throath) Group**
Johannes Kettunen	Northern Ostrobothnia Hospital District, Oulu, Finland	Clinical Groups	**ENT (ear, nose and throath) Group**
Kristiina Aittomäki	Department of Medical Genetics, Helsinki University Central Hospital, Helsinki, Finland	Clinical Groups	**POI (premature ovarian failure) Group**
Fredrik Åberg	Transplantation and Liver Surgery Clinic, Helsinki University Hospital, Helsinki University, Helsinki, Finland	Clinical Groups	**LiverScore Group**
Mitja Kurki	Institute for Molecular Medicine Finland (FIMM), HiLIFE, University of Helsinki, Helsinki, Finland; Broad Institute, Cambridge, MA, United States	FinnGen Analysis working group	FinnGen Analysis working group
Samuli Ripatti	Institute for Molecular Medicine Finland (FIMM), HiLIFE, University of Helsinki, Helsinki, Finland	FinnGen Analysis working group	FinnGen Analysis working group
Mark Daly	Institute for Molecular Medicine, Finland (FIMM), HiLIFE, University of Helsinki, Helsinki, Finland; Broad Institute of MIT and Harvard; Massachusetts General Hospital	FinnGen Analysis working group	FinnGen Analysis working group
Juha Karjalainen	Institute for Molecular Medicine Finland (FIMM), HiLIFE, University of Helsinki, Helsinki, Finland	FinnGen Analysis working group	FinnGen Analysis working group
Aki Havulinna	Institute for Molecular Medicine Finland (FIMM), HiLIFE, University of Helsinki, Helsinki, Finland; Finnish Institute for Health and Welfare (THL), Helsinki, Finland	FinnGen Analysis working group	FinnGen Analysis working group
Juha Mehtonen	Institute for Molecular Medicine Finland (FIMM), HiLIFE, University of Helsinki, Helsinki, Finland	FinnGen Analysis working group	FinnGen Analysis working group
Priit Palta	Institute for Molecular Medicine Finland (FIMM), HiLIFE, University of Helsinki, Helsinki, Finland	FinnGen Analysis working group	FinnGen Analysis working group
Shabbeer Hassan	Institute for Molecular Medicine Finland (FIMM), HiLIFE, University of Helsinki, Helsinki, Finland	FinnGen Analysis working group	FinnGen Analysis working group
Pietro Della Briotta Parolo	Institute for Molecular Medicine Finland (FIMM), HiLIFE, University of Helsinki, Helsinki, Finland	FinnGen Analysis working group	FinnGen Analysis working group
Wei Zhou	Broad Institute, Cambridge, MA, United States	FinnGen Analysis working group	FinnGen Analysis working group
Mutaamba Maasha	Broad Institute, Cambridge, MA, United States	FinnGen Analysis working group	FinnGen Analysis working group

Shabbeer Hassan	Institute for Molecular Medicine Finland (FIMM), HiLIFE, University of Helsinki, Helsinki, Finland	**FinnGen Analysis working group**	**FinnGen Analysis working group**
Susanna Lemmelä	Institute for Molecular Medicine Finland (FIMM), HiLIFE, University of Helsinki, Helsinki, Finland	**FinnGen Analysis working group**	**FinnGen Analysis working group**
Manuel Rivas	University of Stanford, Stanford, CA, United States	**FinnGen Analysis working group**	**FinnGen Analysis working group**
Aarno Palotie	Institute for Molecular Medicine Finland (FIMM), HiLIFE, University of Helsinki, Helsinki, Finland	**FinnGen Analysis working group**	**FinnGen Analysis working group**
Aoxing Liu	Institute for Molecular Medicine Finland (FIMM), HiLIFE, University of Helsinki, Helsinki, Finland	**FinnGen Analysis working group**	**FinnGen Analysis working group**
Arto Lehisto	Institute for Molecular Medicine Finland (FIMM), HiLIFE, University of Helsinki, Helsinki, Finland	**FinnGen Analysis working group**	**FinnGen Analysis working group**
Andrea Ganna	Institute for Molecular Medicine Finland (FIMM), HiLIFE, University of Helsinki, Helsinki, Finland	**FinnGen Analysis working group**	**FinnGen Analysis working group**
Vincent Llorens	Institute for Molecular Medicine Finland (FIMM), HiLIFE, University of Helsinki, Helsinki, Finland	**FinnGen Analysis working group**	**FinnGen Analysis working group**
Hannele Laivuori	Institute for Molecular Medicine Finland (FIMM), HiLIFE, University of Helsinki, Helsinki, Finland	**FinnGen Analysis working group**	**FinnGen Analysis working group**
Taru Tukiainen	Institute for Molecular Medicine Finland (FIMM), HiLIFE, University of Helsinki, Helsinki, Finland	**FinnGen Analysis working group**	**FinnGen Analysis working group**
Mary Pat Reeve	Institute for Molecular Medicine Finland (FIMM), HiLIFE, University of Helsinki, Helsinki, Finland	**FinnGen Analysis working group**	**FinnGen Analysis working group**
Henrike Heyne	Institute for Molecular Medicine Finland (FIMM), HiLIFE, University of Helsinki, Helsinki, Finland	**FinnGen Analysis working group**	**FinnGen Analysis working group**
Nina Mars	Institute for Molecular Medicine Finland (FIMM), HiLIFE, University of Helsinki, Helsinki, Finland	**FinnGen Analysis working group**	**FinnGen Analysis working group**
Joel Rämö	Institute for Molecular Medicine Finland (FIMM), HiLIFE, University of Helsinki, Helsinki, Finland	**FinnGen Analysis working group**	**FinnGen Analysis working group**
Elmo Saarentaus	Institute for Molecular Medicine Finland (FIMM), HiLIFE, University of Helsinki, Helsinki, Finland	**FinnGen Analysis working group**	**FinnGen Analysis working group**
Hanna Ollila	Institute for Molecular Medicine Finland (FIMM), HiLIFE, University of Helsinki, Helsinki, Finland	**FinnGen Analysis working group**	**FinnGen Analysis working group**
Satu Strausz	Institute for Molecular Medicine Finland (FIMM), HiLIFE, University of Helsinki, Helsinki, Finland	**FinnGen Analysis working group**	**FinnGen Analysis working group**
Tuula Palotie	University of Helsinki and Hospital District of Helsinki and Uusimaa, Helsinki, Finland	**FinnGen Analysis working group**	**FinnGen Analysis working group**
Kimmo Palin	University of Helsinki, Helsinki, Finland	**FinnGen Analysis working group**	**FinnGen Analysis working group**
Javier Garcia-Tabuenca	University of Tampere, Tampere, Finland	**FinnGen Analysis working group**	**FinnGen Analysis working group**
Harri Siirtola	University of Tampere, Tampere, Finland	**FinnGen Analysis working group**	**FinnGen Analysis working group**
Tuomo Kiiskinen	Institute for Molecular Medicine Finland (FIMM), HiLIFE, University of Helsinki, Helsinki, Finland	**FinnGen Analysis working group**	**FinnGen Analysis working group**
Jiwoo Lee	Institute for Molecular Medicine Finland (FIMM), HiLIFE, University of Helsinki, Helsinki, Finland; Broad Institute, Cambridge, MA, United States	**FinnGen Analysis working group**	**FinnGen Analysis working group**
Kristin Tsuo	Institute for Molecular Medicine Finland (FIMM), HiLIFE, University of Helsinki, Helsinki, Finland; Broad Institute, Cambridge, MA, United States	**FinnGen Analysis working group**	**FinnGen Analysis working group**
Amanda Elliott	Institute for Molecular Medicine Finland (FIMM), HiLIFE, University of Helsinki, Helsinki, Finland; Broad Institute, Cambridge, MA, USA and Massachusetts General Hospital, Boston, MA, USA	**FinnGen Analysis working group**	**FinnGen Analysis working group**

Kati Kristiansson	THL Biobank / Finnish Institute for Health and Welfare (THL), Helsinki, Finland	**FinnGen Analysis working group**	**FinnGen Analysis working group**
Mikko Arvas	Finnish Red Cross Blood Service / Finnish Hematology Registry and Clinical Biobank, Helsinki, Finland	**FinnGen Analysis working group**	**FinnGen Analysis working group**
Kati Hyvärinen	Finnish Red Cross Blood Service, Helsinki, Finland	**FinnGen Analysis working group**	**FinnGen Analysis working group**
Jarmo Ritari	Finnish Red Cross Blood Service, Helsinki, Finland	**FinnGen Analysis working group**	**FinnGen Analysis working group**
Olli Carpén	Helsinki Biobank / Helsinki University and Hospital District of Helsinki and Uusimaa, Helsinki	**FinnGen Analysis working group**	**FinnGen Analysis working group**
Johannes Kettunen	Northern Finland Biobank Borealis / University of Oulu / Northern Ostrobothnia Hospital District, Oulu, Finland	**FinnGen Analysis working group**	**FinnGen Analysis working group**
Katri Pylkäs	University of Oulu, Oulu, Finland	**FinnGen Analysis working group**	**FinnGen Analysis working group**
Eeva Sliz	University of Oulu, Oulu, Finland	**FinnGen Analysis working group**	**FinnGen Analysis working group**
Minna Karjalainen	University of Oulu, Oulu, Finland	**FinnGen Analysis working group**	**FinnGen Analysis working group**
Tuomo Mantere	Northern Finland Biobank Borealis / University of Oulu / Northern Ostrobothnia Hospital District, Oulu, Finland	**FinnGen Analysis working group**	**FinnGen Analysis working group**
Eeva Kangasniemi	Finnish Clinical Biobank Tampere / University of Tampere / Pirkanmaa Hospital District, Tampere, Finland	**FinnGen Analysis working group**	**FinnGen Analysis working group**
Sami Heikkinen	University of Eastern Finland, Kuopio, Finland	**FinnGen Analysis working group**	**FinnGen Analysis working group**
Arto Mannermaa	Biobank of Eastern Finland / University of Eastern Finland / Northern Savo Hospital District, Kuopio, Finland	**FinnGen Analysis working group**	**FinnGen Analysis working group**
Eija Laakkonen	University of Jyväskylä, Jyväskylä, Finland	**FinnGen Analysis working group**	**FinnGen Analysis working group**
Nina Pitkänen	Auria Biobank / University of Turku / Hospital District of Southwest Finland, Turku, Finland	**FinnGen Analysis working group**	**FinnGen Analysis working group**
Samuel Lessard	Translational Sciences, Sanofi R&D, Framingham, MA, USA	**FinnGen Analysis working group**	**FinnGen Analysis working group**
Clément Chatelain	Translational Sciences, Sanofi R&D, Framingham, MA, USA	**FinnGen Analysis working group**	**FinnGen Analysis working group**
Lila Kallio	Auria Biobank / University of Turku / Hospital District of Southwest Finland, Turku, Finland	**Biobank directors**	**Biobank directors**
Tiina Wahlfors	THL Biobank / Finnish Institute for Health and Welfare (THL), Helsinki, Finland	**Biobank directors**	**Biobank directors**
Jukka Partanen	Finnish Red Cross Blood Service / Finnish Hematology Registry and Clinical Biobank, Helsinki, Finland	**Biobank directors**	**Biobank directors**
Eero Punkka	Helsinki Biobank / Helsinki University and Hospital District of Helsinki and Uusimaa, Helsinki	**Biobank directors**	**Biobank directors**
Raisa Serpi	Northern Finland Biobank Borealis / University of Oulu / Northern Ostrobothnia Hospital District, Oulu, Finland	**Biobank directors**	**Biobank directors**

Sanna Siltanen	Finnish Clinical Biobank Tampere / University of Tampere / Pirkanmaa Hospital District, Tampere, Finland	Biobank directors	Biobank directors
Veli-Matti Kosma	Biobank of Eastern Finland / University of Eastern Finland / Northern Savo Hospital District, Kuopio, Finland	Biobank directors	Biobank directors
Tiina Jokela	Central Finland Biobank / University of Jyväskylä / Central Finland Health Care District, Jyväskylä, Finland	Biobank directors	Biobank directors
Anu Jalanko	Institute for Molecular Medicine Finland (FIMM), HiLIFE, University of Helsinki, Helsinki, Finland	FinnGen Teams	Administration
Auli Toivola	Institute for Molecular Medicine Finland (FIMM), HiLIFE, University of Helsinki, Helsinki, Finland	FinnGen Teams	Administration
Huei-Yi Shen	Institute for Molecular Medicine Finland (FIMM), HiLIFE, University of Helsinki, Helsinki, Finland	FinnGen Teams	Administration
Risto Kajanne	Institute for Molecular Medicine Finland (FIMM), HiLIFE, University of Helsinki, Helsinki, Finland	FinnGen Teams	Administration
Rodos Rodosthenous	Institute for Molecular Medicine Finland (FIMM), HiLIFE, University of Helsinki, Helsinki, Finland	FinnGen Teams	Administration
Mervi Aavikko	Institute for Molecular Medicine Finland (FIMM), HiLIFE, University of Helsinki, Helsinki, Finland	FinnGen Teams	Administration
Helen Cooper	Institute for Molecular Medicine Finland (FIMM), HiLIFE, University of Helsinki, Helsinki, Finland	FinnGen Teams	Administration
Denise Öller	Institute for Molecular Medicine Finland (FIMM), HiLIFE, University of Helsinki, Helsinki, Finland	FinnGen Teams	Administration
Tarja Laitinen	Institute for Molecular Medicine Finland (FIMM), HiLIFE, University of Helsinki, Helsinki, Finland	FinnGen Teams	Administration
Rasko Leinonen	Institute for Molecular Medicine Finland (FIMM), HiLIFE, University of Helsinki, Helsinki, Finland; European Molecular Biology Laboratory, European Bioinformatics Institute, Cambridge, UK	FinnGen Teams	Administration
Henna Palin	Finnish Clinical Biobank Tampere / University of Tampere / Pirkanmaa Hospital District, Tampere, Finland	FinnGen Teams	Administration
Malla-Maria Linna	Helsinki Biobank / Helsinki University and Hospital District of Helsinki and Uusimaa, Helsinki	FinnGen Teams	Administration
Mitja Kurki	Institute for Molecular Medicine Finland (FIMM), HiLIFE, University of Helsinki, Helsinki, Finland; Broad Institute, Cambridge, MA, United States	FinnGen Teams	Analysis
Juha Karjalainen	Institute for Molecular Medicine Finland (FIMM), HiLIFE, University of Helsinki, Helsinki, Finland	FinnGen Teams	Analysis
Pietro Della Briotta Parolo	Institute for Molecular Medicine Finland (FIMM), HiLIFE, University of Helsinki, Helsinki, Finland	FinnGen Teams	Analysis
Arto Lehisto	Institute for Molecular Medicine Finland (FIMM), HiLIFE, University of Helsinki, Helsinki, Finland	FinnGen Teams	Analysis
Juha Mehtonen	Institute for Molecular Medicine Finland (FIMM), HiLIFE, University of Helsinki, Helsinki, Finland	FinnGen Teams	Analysis
Wei Zhou	Broad Institute, Cambridge, MA, United States	FinnGen Teams	Analysis
Masahiro Kanai	Broad Institute, Cambridge, MA, United States	FinnGen Teams	Analysis
Mutaamba Maasha	Broad Institute, Cambridge, MA, United States	FinnGen Teams	Analysis
Zhili Zheng	Broad Institute, Cambridge, MA, United States	FinnGen Teams	Analysis

Hannele Laivuori	Institute for Molecular Medicine Finland (FIMM), HiLIFE, University of Helsinki, Helsinki, Finland	FinnGen Teams	**Clinical Endpoint Development**
Aki Havulinna	Institute for Molecular Medicine Finland (FIMM), HiLIFE, University of Helsinki, Helsinki, Finland; Finnish Institute for Health and Welfare (THL), Helsinki, Finland	FinnGen Teams	**Clinical Endpoint Development**
Susanna Lemmelä	Institute for Molecular Medicine Finland (FIMM), HiLIFE, University of Helsinki, Helsinki, Finland	FinnGen Teams	**Clinical Endpoint Development**
Tuomo Kiiskinen	Institute for Molecular Medicine Finland (FIMM), HiLIFE, University of Helsinki, Helsinki, Finland	FinnGen Teams	**Clinical Endpoint Development**
L. Elisa Lahtela	Institute for Molecular Medicine Finland (FIMM), HiLIFE, University of Helsinki, Helsinki, Finland	FinnGen Teams	**Clinical Endpoint Development**
Mari Kaunisto	Institute for Molecular Medicine Finland (FIMM), HiLIFE, University of Helsinki, Helsinki, Finland	FinnGen Teams	**Communication**
Elina Kilpeläinen	Institute for Molecular Medicine Finland (FIMM), HiLIFE, University of Helsinki, Helsinki, Finland	FinnGen Teams	**E-Science**
Tianduanyi Wang	Institute for Molecular Medicine Finland (FIMM), HiLIFE, University of Helsinki, Helsinki, Finland	FinnGen Teams	**E-Science**
Timo P. Sipilä	Institute for Molecular Medicine Finland (FIMM), HiLIFE, University of Helsinki, Helsinki, Finland	FinnGen Teams	**E-Science**
Oluwaseun Alexander Dada	Institute for Molecular Medicine Finland (FIMM), HiLIFE, University of Helsinki, Helsinki, Finland	FinnGen Teams	**E-Science**
Awaisa Ghazal	Institute for Molecular Medicine Finland (FIMM), HiLIFE, University of Helsinki, Helsinki, Finland	FinnGen Teams	**E-Science**
Anastasia Kytölä	Institute for Molecular Medicine Finland (FIMM), HiLIFE, University of Helsinki, Helsinki, Finland	FinnGen Teams	**E-Science**
Rigbe Weldatsadik	Institute for Molecular Medicine Finland (FIMM), HiLIFE, University of Helsinki, Helsinki, Finland	FinnGen Teams	**E-Science**
Sanni Ruotsalainen	Institute for Molecular Medicine Finland (FIMM), HiLIFE, University of Helsinki, Helsinki, Finland	FinnGen Teams	**E-Science**
Jaska Uimonen	Institute for Molecular Medicine Finland (FIMM), HiLIFE, University of Helsinki, Helsinki, Finland	FinnGen Teams	**E-Science**
Kati Donner	Institute for Molecular Medicine Finland (FIMM), HiLIFE, University of Helsinki, Helsinki, Finland	FinnGen Teams	**Genotyping**
Anu Loukola	Helsinki Biobank / Helsinki University and Hospital District of Helsinki and Uusimaa, Helsinki	FinnGen Teams	**Sample Collection Coordination**
Päivi Laiho	THL Biobank / Finnish Institute for Health and Welfare (THL), Helsinki, Finland	FinnGen Teams	**Sample Logistics**
Tuuli Sistonen	THL Biobank / Finnish Institute for Health and Welfare (THL), Helsinki, Finland	FinnGen Teams	**Sample Logistics**
Essi Kaiharju	THL Biobank / Finnish Institute for Health and Welfare (THL), Helsinki, Finland	FinnGen Teams	**Sample Logistics**
Markku Laukkanen	THL Biobank / Finnish Institute for Health and Welfare (THL), Helsinki, Finland	FinnGen Teams	**Sample Logistics**
Elina Järvensivu	THL Biobank / Finnish Institute for Health and Welfare (THL), Helsinki, Finland	FinnGen Teams	**Sample Logistics**
Sini Lähteenmäki	THL Biobank / Finnish Institute for Health and Welfare (THL), Helsinki, Finland	FinnGen Teams	**Sample Logistics**
Lotta Männikkö	THL Biobank / Finnish Institute for Health and Welfare (THL), Helsinki, Finland	FinnGen Teams	**Sample Logistics**
Regis Wong	THL Biobank / Finnish Institute for Health and Welfare (THL), Helsinki, Finland	FinnGen Teams	**Sample Logistics**
Auli Toivola	THL Biobank / Finnish Institute for Health and Welfare (THL), Helsinki, Finland	FinnGen Teams	**Sample Logistics**

Minna Brunfeldt	THL Biobank / Finnish Institute for Health and Welfare (THL), Helsinki, Finland	FinnGen Teams	**Registry Data Operations**
Susanna Lemmelä	Institute for Molecular Medicine Finland (FIMM), HiLIFE, University of Helsinki, Helsinki, Finland	FinnGen Teams	**Registry Data Operations**
Sami Koskelainen	THL Biobank / Finnish Institute for Health and Welfare (THL), Helsinki, Finland	FinnGen Teams	**Registry Data Operations**
Tero Hiekkalinna	THL Biobank / Finnish Institute for Health and Welfare (THL), Helsinki, Finland	FinnGen Teams	**Registry Data Operations**
Teemu Paajanen	THL Biobank / Finnish Institute for Health and Welfare (THL), Helsinki, Finland	FinnGen Teams	**Registry Data Operations**
Priit Palta	Institute for Molecular Medicine Finland (FIMM), HiLIFE, University of Helsinki, Helsinki, Finland	FinnGen Teams	**Sequencing Informatics**
Shuang Luo	Institute for Molecular Medicine Finland (FIMM), HiLIFE, University of Helsinki, Helsinki, Finland	FinnGen Teams	**Sequencing Informatics**
Mary Pat Reeve	Institute for Molecular Medicine Finland (FIMM), HiLIFE, University of Helsinki, Helsinki, Finland	FinnGen Teams	**Trajectory**
Shanmukha Sampath Padmanabhuni	Institute for Molecular Medicine Finland (FIMM), HiLIFE, University of Helsinki, Helsinki, Finland	FinnGen Teams	**Trajectory**
Marianna Niemi	University of Tampere, Tampere, Finland	FinnGen Teams	**Trajectory**
Harri Siirtola	University of Tampere, Tampere, Finland	FinnGen Teams	**Trajectory**
Javier Gracia-Tabuenca	University of Tampere, Tampere, Finland	FinnGen Teams	**Trajectory**
Mika Helminen	University of Tampere, Tampere, Finland	FinnGen Teams	**Trajectory**
Tiina Luukkaala	University of Tampere, Tampere, Finland	FinnGen Teams	**Trajectory**
Iida Vähätalo	University of Tampere, Tampere, Finland	FinnGen Teams	**Trajectory**
Iina Laak	Institute for Molecular Medicine Finland (FIMM), HiLIFE, University of Helsinki, Helsinki, Finland	FinnGen Teams	**Data protection officer**
Marco Hautalahti	Finnish Biobank Cooperative - FINBB	FinnGen Teams	**FINBB - Finnish biobank cooperative**
Johanna Mäkelä	Finnish Biobank Cooperative - FINBB	FinnGen Teams	**FINBB - Finnish biobank cooperative**
Saija Haapa-Paananen	Finnish Biobank Cooperative - FINBB	FinnGen Teams	**FINBB - Finnish biobank cooperative**
Sarah Smith	Finnish Biobank Cooperative - FINBB	FinnGen Teams	**FINBB - Finnish biobank cooperative**
Tom Southerington	Finnish Biobank Cooperative - FINBB	FinnGen Teams	**FINBB - Finnish biobank cooperative**
Meri Lähteenmäki	Finnish Biobank Cooperative - FINBB	FinnGen Teams	**FINBB - Finnish biobank cooperative**

UK Biobank study description and methods

The UK Biobank is a large population-based prospective cohort study from the United Kingdom with rich phenotypic and genetic data on 500,000 individuals aged 40–69 at enrollment[18]. Available genetic data currently include genome-wide imputed data for almost all participants[18]. Briefly, genotyping was performed using Affymetrix UK Biobank Axiom (450,000 samples) and Affymetrix UK BiLEVE axiom (50,000 samples) arrays. Subsequently, the genetic data were imputed to the Haplotype Reference Consortium panel and UK10K + 1000 Genomes panels (version 3 imputed data). We removed any samples that had withdrawn their consent, samples that were outliers for heterozygosity or missingness, individuals with putative sex chromosome aneuploidy and individuals with a mismatch between self-reported and genetically inferred sex, as determined by central quality-control. Use of the UK Biobank was performed under application number 17488.

We ran GWAS for NI-DCM and NICM, defined by ICD coding (ICD10 I42.0 for NI-DCM; ICD10 I42.0, ICD10 I50.1 or ICD9 4281 for NICM). Prevalent and incident cases were combined. Cases were excluded from the analysis if they had antecedent codes for acute coronary syndrome and/or revascularization, as previously described[17]; controls with codes for general heart failure (as defined in [19]) were also removed from the analysis.

We used REGENIE[14] v3.1.1 to run the GWAS. Variants from the genotyping array were used for null-model fitting in step 1. In both step 1 and 2, we adjusted for age, age^2, sex, PC1-4, and PCS among PC5-20 if associated with either NI-DCM or NICM at nominal significance (P<0.05 among unrelated samples). An approximate Firth's correction was used for variants reaching nominal P<0.05 in an initial test; standard errors were computed by back-correcting from the Firth's beta and Firth's P-value.

Massachusetts General Brigham Biobank study description and methods

The Massachusetts General Brigham Biobank (MGB) is an ongoing observational biobank enrolling participants from a multicenter health system in Massachusetts, USA[20]. Participants are enrolled with broad-based consent collected by local research coordinators, either as part of a collaborative research study or electronically through a patient portal[21]. Demographic data, blood samples and surveys are collected at baseline and linked to electronic health record data. All adult patients provided informed consent to participate. A small number of children were enrolled with IRB-approved assent forms; upon reaching 18 years of age all enrolled children had to provide consent or were removed from the study. The Human Research Committee of MGB approved the Biobank protocol (2009P002312).

All samples were genotyped using the GlobalScreeningArray version 1. The genotype array data underwent stringent QC. Variant QC consisted of removal of variants with allele count <2, missingness >2%, Hardy Weinberg equilibrium test *P*-value <1e-6 (in each continental super-population), and those with discordant frequencies as compared to gnomAD (X^2 statistic >300; applied in each continental super-population). Sample QC consisted of removal of outliers for heterozygosity or missingness, removal of samples with a mismatch between inferred and self-reported sex, and removal of samples with a mismatch between exome sequencing and array calls. Principal component analysis and relatedness inference were performed using PC-Relate[22] and PC-Air[23], while ancestry labels (for continental super-populations) were learned from a k-nearest-neighbor model trained on 1000Genomes project data[3]. Following these stringent QC procedures, data were subsequently genome-wide imputed to the TOPMed imputation panel (r2) on the Michigan Imputation Server (submitted by batch)[24].

We ran GWAS for NI-DCM and NICM, defined by ICD coding (I42.0 for NI-DCM, I42.0 and I50.1 for NICM). Prevalent and incident cases were combined. Cases were excluded from the analysis if they had coronary disease (irrespective of timing), as defined centrally by the biobank. Controls with codes for general heart failure (as defined in [19]) were also removed from the analysis.

We used REGENIE[14] v3.1.1 to run the GWAS. Variants from the genotyping array were used for null-model fitting in step 1. In both step 1 and 2, we adjusted for age, age^2, sex, PC1-4, and PCS among PC5-20 if associated with either NI-DCM or NICM at nominal significance ($P<0.05$ among unrelated samples). An approximate Firth's correction was used for variants reaching nominal $P<0.05$ in an initial test; standard errors were computed by back-correcting from the Firth's beta and Firth's P-value.

UK Biobank is generously supported by its founding funders the Wellcome Trust and UK Medical Research Council, as well as the British Heart Foundation, Cancer Research UK, Department of Health, Northwest Regional Development Agency and Scottish Government.

All of Us Research Program description and methods

Sequencing and quality control

Each Genome Center performed quality control (QC) of the specimens obtained from the All of Us Biobank. Sample preparation and normalization and DNA library construction have been reported previously[25], after which samples underwent whole genome sequencing (WGS). Details on sequencing, variant calling and quality control are described in the program's genomic data quality report [https://support.researchallofus.org/hc/en-us/articles/4617899955092-All-of-Us-Genomic-Quality-Report-]. In brief, processing consisted of an initial per-sample QC, including fingerprint concordance (array vs. WGS data), sex concordance (genetically determined vs. self-reported), cross-individual contamination rate and coverage to detect major errors, such as sample swaps or contamination. Participants who failed these tests were removed from the release. The WGS variants were then called jointly to reduce systematic biases. Additional sample QC procedures were then performed on the joint callsets, including hard threshold flagging (e.g., number of SNPs: < 2.4M and > 5.0M) and population outlier flagging. Variants QC was performed after sample QC, flagging specific variants in the callset. Processes included hard threshold filters (e.g., ExcessHet, QUAL score) and Allele-Specific Variant Quality Score Recalibration (AS-VQSR or VQSR).

For PRS analyses, we focussed on the v7 short-read ACAF dataset with split variants (ie multi-allelic variants were split to represent separate bi-allelic variants). On this dataset, we performed additional QC steps at the genotype and variant level. Specifically, we restricted genotypes to those that passed the central QC procedures (ie, FT==PASS) and have a Genotype Quality value > 20. We then filtered out variants that were monomorphic or had call rates <95%.

For analyses of rare genetic variants, we used the v7 short-read exome dataset with split variants. On this dataset, we performed additional QC steps at the genotype and variant level. Specifically, we restricted to genotypes that passed central QC procedures (ie, FT==PASS) and had Genotype Quality value >20; we then filtered out variants that were monomorphic or had call rates <90%. Based on these variants, we defined carriers of rare pathogenic or likely pathogenic variants based on the following criteria: variants i) that were protein-coding variants within ClinGen strong/definitive genes for DCM (*BAG3*, *DES*, *TNNT2*, *FLNC*, *PLN*, *LMNA*, *MYH7*, *RBM20*, *SCN5A*, *TNNC1*, *TTN* and *DSP*), ii) had minor allele frequency <0.1% in the dataset and <0.1% in each major continental super-population in gnomAD exomes v2 (ref.[26]), iii) were reported in ClinVar as likely pathogenic (LP) or pathogenic (P) (following previous ClinVar filtering procedures[27,28]; pull updated in April 2023), and iv) were reported in ClinVar with a phenotypic assertion of DCM. In addition, we included rare predicted loss-of-function (LOF) variants (determined by LOFTEE; https://github.com/konradjk/loftee) for a subset of genes (*BAG3*, *FLNC*, *LMNA*, *TTN* and *DSP*) restricting to rare high-confidence LOF variants with no flags and restricting to canonical transcripts (*BAG3*, *FLNC*, *LMNA* and *DSP*) or cardiac-expressed exons (*TTN*).

For additional sample QC, we removed flagged participants (population outliers), possible duplicates, samples with a call rate of < 90%, and individuals with missing genetically-determined sex, resulting in 242,902 participants in the current analysis dataset. Based on central relatedness inference, we then further restricted all analyses to samples with complete EHR linkage and who were genetically unrelated, leaving 195,533 samples.

Principal component analysis

All of Us applied the analysis pipeline using the gnomAD v3.1 release [https://gnomad.broadinstitute.org/news/2020-10-gnomad-v3-1-new-content-methods-

annotations-and-data-availability/] to calculate principal components (PCs) of ancestry and assign ancestry labels. Specifically, AoU first identified 150,229 high-quality sites that can be called accurately in both the Human Genome Diversity Project (HGDP) and 1000 Genomes (1000G) dataset (training sample) and the All of Us dataset. High-quality sites were defined as autosomal, bi-allelic single nucleotide variants (SNVs) with a minor allele frequency > 0.1% and a call rate > 99%. These sites were then LD-pruned with a cut-off of r2 = 0.1. All of Us centrally calculated the first 16 PCs in the training sample (HGDP and 1000G, using the hwe_normalized_pca() Hail function) with high-quality SNVs and projected the All of Us samples into the PCA space to generate the first 16 PCs. The number of PCs was determined in the gnomAD resource that the first 16 PCs captured global ancestry variation well. A clear drop in information content was observed for higher PCs.

Ancestry assignments

To assign ancestry categories to the WGS participants, All of Us trained a random forest classifier on the HGDP and 1000G samples with known ancestry labels using the 16 PCs and applied the model to the AoU sample. Therefore, available ancestry categories were those used in the gnomAD, HGDP and 1000G resources, including African, Latino/Native American/Ad Mixed American, East Asian, Middle Eastern, European, Other, and South Asian. Ancestry categories were assigned to participants based on the probability generated by the random forest model. A cut-off of 75% was used, and all remaining samples were assigned to the "Other" group. To assess the accuracy of the predictions, AoU calculated a concordance metric between the self-reported ethnicity and the ancestry predictions, and reported an estimate of 0.915 [https://support.researchallofus.org/hc/en-us/articles/4617899955092-All-of-Us-Genomic-Quality-Report-].

Phenotype definitions

NI-DCM and NICM were defined using International Classifications of Disease (ICD) 10 codes: I42.0 “Dilated cardiomyopathy” for NI-DCM; I42.0 “Dilated cardiomyopathy” or I50.1 “Left heart failure” for NICM. For both phenotypes cases were excluded if they had antecedent codes for acute coronary events and/or revascularization procedures: Exclusions were based on ICD10-CM codes I21 (and all subgroupings), I22 (and all subgrouping), I23 (and all subgrouping), I24 (and all subgrouping), I25.2; ICD9-CM codes 410 (and all subgrouping), I411 (and all subgrouping), I412 (and all subgrouping); Current Procedure Terminology (CPT) codes 33510, 33511, 33512, 33533, 33534, 33535, 33536, 92920, 92921, 92924, 92925, 92928, 92933, 92934, 92937, 92938, 92941, 92943, 92944, 92973, 92975.

For broad heart failure we used the central *All of Us* defined “Heart failure” phenotype. Systolic heart failure was defined using ICD10-CM codes: I50.2 “Systolic congestive heart failure”, and subcodes I50.20 “Unspecified systolic heart failure”, I50.21 “Acute systolic (congestive) heart failure”, I50.22 “Chronic systolic (congestive) heart failure, and I50.23 “Acute on chronic systolic (congestive) heart failure”. Hypertension was defined using SNOMED codes 1201005 “Benign essential hypertension”, 59621000 “Essential hypertension”, 63287004 “Benign essential hypertension in obstetric context”, 72022006 “Essential hypertension in obstetric context”, 78808002 “Essential hypertension complicating AND/OR reason for care during pregnancy”, 78975002 “Malignant essential hypertension”; using ICD9-CM codes: 401 “Essential hypertension”, 401.0 “Malignant essential hypertension”, 401.1 “Benign essential hypertension”, 401.9 “Unspecified essential hypertension”; and using ICD10-CM code: I10 “Essential (primary) hypertension”. Acute myocardial infarction was defined using SNOMED codes 401303003 “Acute ST segment elevation myocardial infarction”, 401314000 “Acute non-ST segment elevation myocardial infarction”, 54329005 “Acute myocardial infarction of anterior wall”, 57054005 “Acute

myocardial infarction”, 65547006 “Acute myocardial infarction of inferolateral wall”, 70211005 “Acute myocardial infarction of anterolateral wall”, 70422006 “Acute subendocardial infarction”, 76593002 “Acute myocardial infarction of inferoposterior wall”; ICD9-CM codes: 410 “Acute myocardial infarction”, 410.0 (and subcodes 410.0, 410.00, 410.01, 410.02, 410.1, 410.10, 410.11, 410.12, 410.20, 410.21, 410.22, 410.3, 410.30, 410.31, 410.32, 410.4, 410.42, 410.5, 410.50, 410.51, 410.52, 410.70, 410.71, 410.72, 410.8, 410.80, 410.81, 410.9, 410.91, 410.92); and ICD10-CM codes I21 “Acute myocardial infarction” (and subcodes I21.01, I21.02, I21.09, I21.11, I21.19, I21.21, I21.29, I21.3, I21.4, I21.9), I23.1 “Atrial septal defect as current complication following acute myocardial infarction”, I23.3 “Rupture of cardiac wall without hemopericardium as current complication following acute myocardial infarction”, I23.6 “Thrombosis of atrium, auricular appendage, and ventricle as current complications following acute myocardial infarction”, and I23.8 “Other current complications following acute myocardial infarction”

Acknowledgements

We gratefully acknowledge All of Us participants for their contributions, without whom this research would not have been possible. We also thank the National Institutes of Health’s All of Us Research Program for making available the participant data [and/or samples and/or cohort] examined in this study.

Processing of summary statistics for molecular trait MR and colocalization

INDELS and ambiguous variants were removed; all variants were lifted to GRCh37 using liftOver; variants were restricted to those with at least 70% of total cases (GWAS-DCM) or at least 70% of total effective sample size (MTAG-DCM) contributing to the outcome GWAS; variants were restricted to those found in both the exposure and outcome dataset; and variants were restricted to those found in the LD reference - a reference built from high-quality hard-called imputed data from 5k random European UK Biobank participants.

Processing of summary statistics for MR analysis

For any given MR comparison, we first harmonized summary statistics by i) lifting over to GRCh37 if on a different build, ii) removing ambiguous variants and indels, iii), removing variants with <70% of the total case numbers contributing to the DCM GWAS, iv) removing variants with MAF<1% in either study (if present in the summary statistics), v) removing variants with imputation accuracy <0.3 in either study (if present in the summary statistics), vi) aligning effect and reference alleles, and vii) restricting to variants also present in the LD reference (built from 5k random European ancestry samples from UKB). After the harmonization, prior to MR, variants were filtered and pruned based on genome-wide significance (P<5e-8) and r^2 <0.0005 taking 10Mb windows.

Assessment of NI-DCM and NICM phenotypes from biobank data

We aimed to assess the validity of our biobank-based non-ischemic DCM (NI-DCM) phenotype. For our strict NI-DCM phenotype - from a meta-analysis of FinnGen, UK Biobank and MGB - we observed comparable heritability estimates ($h^2_{SNP,\ liability}$=14.1%, SE=1.6%; by LDSC[29]) as compared to the GWAS meta-analysis of clinically ascertained DCM cases ($h^2_{SNP,\ liability}$=16.4%, SE=1.8%) (**Supplementary Table 4**). Furthermore, biobank-ascertained NI-DCM was strongly genetically correlated with clinically ascertained DCM (r_g = 0.73, SE=0.07, by bivariate LD score regression [LDSC[29,30]]; **Supplementary Table 5**). It should be noted that this genetic correlation is likely attenuated by a somewhat divergent LD structure between both meta-analyses - given the isolated structure of Finland as compared to non-Finnish Europeans[16]. Indeed, when comparing the clinical Garnier et al. dataset to a meta-analysis of UKB and MGB, we found an even higher point estimate for the genetic correlation between biobank-based NI-DCM and clinically-ascertained DCM (r_g = 0.82, SE=0.22, *P*=0.0002). Taken together, these analyses provide strong genetic support for our strict, biobank-based phenotypic construct of DCM.

For comparison with our NI-DCM construct, we also evaluated a more permissive, biobank-based disease definition of non-ischemic cardiomyopathy (NICM)—meant to capture LV dysfunction in the absence of antecedent ischemic heart disease[17]—as pursued in prior GWAS of DCM from biobank populations. As compared to the NI-DCM phenotype, we observed a substantially lower heritability estimate with the NICM phenotype ($h^2_{SNP,\ liability}$=6.9%, SE=0.7%) and fewer significant loci (21 vs 26), despite substantially more cases (N=13,478) (**Supplementary Table 4** and **Extended Data Figure 1**). These results highlight that a more stringent DCM phenotype may reveal a stronger genetic/heritable component. Therefore, for our biobank-based analyses, we proceeded with the strict NI-DCM phenotype.

Novelty of significant loci

We then evaluated the novelty of loci identified in our GWAS and MTAG analysis. In GWAS-DCM, of 38 distinct loci, we identified 27 loci that were not identified in previously-published DCM GWAS papers (**Extended Data Figure 3**). We then queried two published GWAS papers for DCM-relevant traits, including i) a study of DCM-relevant LV functional traits (LVEF, LVESVi, LVEDVi, and SVi) by Pirruccello et al.[31] and ii) a study of general HF (including a GWAS and MTAG analysis) by Levin et al.[32] Of the 27 novel loci, 12 were also not identified in these relevant traits (**Supplementary Table 8**).

In MTAG-DCM, of 65 significant loci, we identified 50 loci not identified in previously-published GWAS papers for DCM (**Extended Data Figure 3**). Of these novel loci, 24 were also not identified for DCM-relevant LV functional traits and general HF, in the studies by Pirruccello et al. and Levin et al. (**Supplementary Table 12**).

Assessment of pleiotropy for significant loci

We aimed to identify pleiotropic effects for the lead variants identified in our GWAS-DCM and MTAG-DCM analyses (**Supplementary Tables 34-38**). First, we queried the Cardiovascular Disease Knowledge Portal (CVDKP; https://cvd.hugeamp.org/) to identify pleiotropic associations for relevant cardiovascular diseases and quantitative traits. At the suggestive significance level set by the portal, 33 of 38 GWAS-DCM loci showed potential pleiotropic associations with relevant traits (**Supplementary Tables 34 and 38**), which include cardiac MRI traits, ECG traits, HF, atrial fibrillation, and heart rate. In contrast, only two loci showed pleiotropic effects on coronary artery disease, of which one had a discordant effect between DCM and coronary disease (*ADAMTS7*). Similarly, of 65 MTAG-DCM loci, 40 loci showed potential pleiotropic associations with relevant traits (excluding MRI traits; **Supplementary Tables 36 and 38**); only three loci showed pleiotropic effects on coronary artery disease (again including the discordant *ADAMTS7* locus).

The above look-up was based on the CVDKP, which is focused on cardiovascular traits. As such, this pleiotropy look-up was naturally biased towards potentially relevant traits, and would miss important pleiotropic associations outside of the cardiovascular system. We therefore performed a second look-up using a publicly-available phenome-wide disease analysis (PheWAS) from the UK Biobank (**Supplementary Tables 35 and 37**). Reassuringly, the vast majority of suggestive associations involved arrhythmia, conduction disease, hypertension, heart failure, and related cardiovascular diseases; there were only limited suggestive associations in other organ systems. These findings show that the phenotypic consequences of our DCM loci largely involve the cardiovascular system; furthermore, these results support the validity of DCM loci.

Replication analyses

We aimed to assemble a large replication cohort to validate the findings from our discovery analyses. To this end, we combined data from a parallel GWAS effort for DCM from the Heart Failure Molecular Epidemiology for Therapeutic Targets (HERMES) consortium[33], data from the Million Veteran Program (MVP), and data from the All of Us Research Program. In our approach, we were careful to include only samples that were not already included in our discovery datasets (as outlined in more detail for each dataset below), which yielded a replication meta-analysis of up to 13,258 cases and 1,435,678 controls.

HERMES

In a parallel effort, the HERMES consortium recently released a manuscript describing a European-ancestry GWAS meta-analysis for DCM. This effort included both 'hard DCM' cases and 'broad' DCM cases (defined as LV systolic dysfunction in absence of a number of secondary causes), totalling 14,255 cases and 1,199,156 controls. We refer to the associated preprint for details on genotyping, phenotyping and GWAS analyses[33]. We note that a substantial number of 'hard DCM' datasets from HERMES also contributed to the present GWAS-DCM. Therefore, to remove the possibility of overlapping samples, the HERMES meta-analyses were rerun restricting to non-overlapping datasets. These included BioVU, CHB, deCODE, DiscovEHR-GSA, DiscovEHR-Omni, EstBiobank, GoDARTS-ILLUMINA, PIVUS, ULSAM, DCM-UCL, and GEL. The datasets were combined using an inverse-variance-weighted fixed-effects meta-analysis, totalling up to 8,480 cases and 756,404 controls. The lead variants from GWAS-DCM and MTAG-DCM were extracted from this meta-analysis.

MVP

Cohort description

The Veterans Affairs Million Veteran Program (MVP) started recruiting US military veterans from 63 Veterans Affairs (VA) facilities across the United States in 2011 (ref.[34]). Veterans aged 18 years and older are recruited into MVP where participants are linked to VA electronic health records (EHR), complete a questionnaire, and submit a blood sample at enrollment. The EHR includes information on inpatient International Classification of Disease (ICD) diagnosis codes, Current Procedural Terminology (CPT) procedure codes, and clinical laboratory measurements. Genotyping and quality control in MVP has been reported previously[35,36] and are summarized in detail below

Genotyping and quality control

Specimen collection and genotype quality control have been described in detail before[35,36]. In brief, blood specimens were collected at recruitment sites across the country then shipped within 24 hours to the VA Central Biorepository in Boston, MA for processing and storage. Study participants were genotyped using a customized Affymetrix Axiom biobank array (the MVP 1.0 Genotyping Array), containing 723,305 variants. Duplicate samples were excluded from the genetic analysis. Additional exclusion criteria included: samples with observed heterozygosity greater than the expected heterozygosity, missing genotype call rate greater than 2.5%, and incongruence between sex inferred from genetic information and gender extracted from phenotype data. Probes with high missingness (>20%), those that were monomorphic, or those with a Hardy Weinberg Equilibrium p<1x10-06 in both the overall cohort and within one of the 3 major harmonized race/ethnicity and genetic ancestry (HARE)[37] race or ethnicity groups (non-Hispanic White, non-Hispanic Black, or Hispanic/Latino). See below for HARE methods.

KING[13] was used to measure relatedness between individuals in the sample. ADMIXTURE[38] was used to calculate loadings on five 1000Genomes reference populations[3] representing the majority of ancestry within the United States - GBR (British), PEL (Peruvian), YRI (Yoruba/Nigerian), CHB (Han Chinese), and LWK (Luhya/Kenyan). Pre-analysis QC was performed to remove SNPs that were rare (MAF < 1%), had high missingness (> 5%), or had excess heterozygosity (Fst < -0.1). SNPs that passed filters were then merged with the 1000 Genomes phase 3 reference panel[3], removing SNPs that were not shared in both filesets. LD pruning was performed using the 'indep-pairwise' command in PLINK version 1.9, with window = 1000, shift = 50, and r2 = 0.05, and excluding loci with complicated LD structure (i.e. MHC and KIR). Principal components (PCs) were computed using plink2 (ref.[11]).

The HARE approach, developed by MVP, was used to assign individuals to populations or groups[37]. This machine learning algorithm leverages information from both the self-identified race/ethnicity data from the survey and data from the genome-wide array to create respective variables for downstream analyses. HARE categorized Veterans into four mutually exclusive groups: (1) non-Hispanic White, (2) non-Hispanic Black, (3) Hispanic or Latino, or (4) Asian. High concordance was observed between HARE-defined non-Hispanic White and non-Hispanic Black populations, and genetically inferred European and African ancestry populations, respectively.

Imputation to TOPMed Imputation Panel

Genetic imputation was performed to the TOPMed reference panel[24]. Pre-phasing was performed using SHAPEIT4 (v 4.1.3; ref.[39]) using 20MB chunks and 5MB overlap, and Minimac4 (ref.[40]) software was used for imputation using 20MB chunks with 3MB overlap between chunks.

Genetically Inferred Ancestry (GIA) definition

To estimate ancestry, we obtained a reference dataset from the 1000 Genomes Project and used the smartpca module in the EIGENSOFT package (https://github.com/DReichLab/EIG) to project the PC loadings from a group of unrelated individuals in the reference dataset. We merged this dataset with the MVP dataset and ran smartpca to project the PCA loadings from the reference dataset. We trained a random forest classifier using continental ancestry meta-data based on the top 10 principal components from the reference training data to define genetically inferred ancestry. We then applied this random forest to the predicted MVP PCA data and assigned ancestries to individuals with a probability greater than 50%. Those with a probability less than 50% for any particular ancestry group were excluded from the study. The final GIA population classifications were (1) African (AFR), (2) Admixed American (AMR), (3) East Asian (EAS), (4) European (EUR), or (5) South Asian (SAS).

Cardiomyopathy Phenotyping

NI-DCM cases and controls were defined using International Classification of Diseases, 9th or 10th Revision (ICD-9; ICD-10) billing codes. In MVP, the version 21.1 clinical data freeze was used, which contains EHR data up to September 30, 2021. Cases were defined by the presence of ‘dilated cardiomyopathy’ code (I42.0) excluding individuals with prior ischemic cardiomyopathy (I25.5) or coronary artery disease (CAD; I21-I24, I25.2, 410-412), or presence of a CAD code with 30 days after their first DCM code. Controls were defined by a lack of DCM code then individuals were excluded if they ever had codes for heart failure, hypertrophic cardiomyopathy (I42.1, I42.2, 425.1), alcoholic cardiomyopathy (I42.6, 425.5), peripartum cardiomyopathy (O90.3, 674.5), secondary cardiomyopathy (425.9), or drug induced cardiomyopathy (I42.7). Date of first event was defined as the date of the occurrence of the first code. This left a total of 3,964 cases (1,239 AFR, 223 AMR, 2,502 EUR) and 522,610 controls (99,878 AFR, 53,475 AMR, 369,257 EUR) for GWAS analysis.

GWAS

A case-control genome-wide association analysis (GWAS) for DCM was performed within each GIA group using REGENIE, then combined in an inverse variance weighted meta-analysis using GWAMA. Only AFR, AMR, and EUR had enough cases for analysis. A mixed model approach was implemented with adjustment for age at study enrollment, biological sex, and the first 10 genetic PCs. The lead variants from GWAS-DCM and MTAG-DCM were extracted from this meta-analysis.

All of Us

Details on sequencing and DCM phenotyping in the All of Us Research Program are described earlier in this document. For purposes of replication, we ran a GWAS analysis for our NI-DCM phenotype. Since the MGB health system contributed some samples to All of Us, we took a restrictive approach to minimize the potential for sample overlap between discovery and replication. In particular, we removed any sample in All of Us with a ZIP code from Massachusetts. This procedure left 815 NI-DCM cases, and 156,209 controls. We then used REGENIE v3.2.2 to perform a GWAS for the NI-DCM phenotype, using an approximate Firth's regression model. The lead variants from GWAS-DCM and MTAG-DCM were extracted from this multi-ancestry analysis.

Meta-analysis and quality-control

To combine data from the several replication cohorts, we performed an inverse-variance-weighted fixed-effects meta-analysis. This meta-analysis included up to 13,258 cases and 1,435,678 controls. We then filtered these results based on several criteria. First, we retained variants with MAF>1% in the replication meta-analysis and with at least 1000 cases contributing to the replication meta-analysis. Second, per locus, we restricted to the single strongest lead variant in

discovery. This procedure left qualifying replication results for 36/38 GWAS-DCM loci and for 64/65 MTAG-DCM loci. *P*-values were computed as one-sided *P*-values taking into account the direction of effect in discovery.

We first assessed the calibration of effect sizes between replication and discovery. When restricting to previously-established DCM loci, we found that effect sizes in replication were attenuated to ~0.5 of the GWAS-DCM discovery effect sizes. For MTAG-DCM, previously-established loci were attenuated to ~0.56 of discovery. Similar calibration was seen also when assessing all loci (**Extended Data Figure 4**). The attenuation of effect sizes is likely a reflection of i) the broader case definition used in most of the HERMES cohorts - for which we established a substantially lower heritability estimate - and ii) the older age of DCM cases included in MVP. Other contributory factors may be the inclusion of several non-European ancestry samples from MVP and AoU, and Winner's curse inflating effect sizes in discovery. These last points do not seem substantial, however, as restriction to European ancestry samples did not meaningfully alter effect sizes, and effect size calibration was highly similar between known and novel loci on average.

Power calculations

We then computed the expected power in replication. To this end, we computed the effective sample size for each variant in each contributing dataset, computed using the formula *4/(1/cases + 1/controls)*, and then computed the meta-analysis effective sample size as the sum of these values. We then used the function *genpwr.calc()* in R package genpwr to compute power for each variant. We used the effective sample size in replication, the minor allele frequency in replication, and the 'attenuated' effect sizes based on discovery as input; we computed power assuming a logistic additive model. The attenuated effect sizes were computed based on the effect size

attenuation based on previously-established DCM loci only. Power was computed at the 'nominal' level (one-sided alpha=0.05) and at the Bonferroni-corrected level (one-sided alpha=0.05/number of testable loci). To then calculate the total number of expected replicating loci, we took the sum of the power values across loci. Assuming all discovery loci are true and assuming homogeneous effect size attenuation across loci, we estimated that we had power to replicate ~35.6 / 36 GWAS-DCM loci at the nominal level, and ~31.8 / 36 loci at the Bonferroni-corrected level. When considering only novel loci, we had power to detect ~24.6 / 25 GWAS-DCM loci at the nominal level and at ~21.7 / 25 loci at the Bonferroni-corrected level. For MTAG-DCM, we calculated that we had power to replicate ~60.4 / 64 loci at the nominal level and ~43.2 / 64 loci at the Bonferroni-corrected level. When considering only MTAG loci that were not identified in GWAS-DCM, we calculated that we had power to replicate ~28 / 31 loci at the nominal level and ~19.1 / 31 loci at the Bonferroni-corrected level.

Replication rates and results

For GWAS-DCM, we found that 36/36 (100%) of loci were concordant in direction of effect, 33/36 loci reached the nominal significance level (92%), and 26/36 loci (72%) were replicated at the Bonferroni-corrected level (**Extended Data Figure 4**). When considering only novel loci, 23/25 reached the nominal level (92%) and 18/25 reached the Bonferroni-corrected level (72%). Of non-replicating loci (*P*>0.05) two were near Mendelian cardiomyopathy genes (*PLN* and *FHOD3*). We posit that differences in genetic architecture (eg, tagging of causal variants) might underlie the difference, although this can not be proven at this time. The third non-replicating locus was near *PPP1R3C.*

For MTAG-DCM, we found that 62/64 loci (97%) were concordant in direction of effect, 56/64 (88%) reached the nominal level, and 36/64 (56%) reached the Bonferroni-corrected level

(**Extended Data Figure 4**). When considering only loci not already identified in GWAS-DCM, we found that 25/31 (81%) reached the nominal level, and 16/31 (52%) reached the Bonferroni-corrected significance level. Of note, the observed replication rates for MTAG-DCM were only slightly lower than what could be expected based on our power calculations. Of discordant loci, one was near *CSRP3* (a Mendelian cardiomyopathy gene) and one near *IGFBP3*.

Overall, the replication analyses demonstrate a substantial replicability of our initial GWAS-DCM findings. Secondly, the replication analyses provide reassurance of our MTAG approach to identify genetic signals for DCM.

HERMES banner authors

HERMES Consortium

Bitten Aagaard[1], Erik Abner[2], Lance Adams[3], Peter Almgren[4], Charlotte Andersson[5,6], Krishna G. Aragam[7,8,9], Johan Ärnlöv[10,11], Folkert W. Asselbergs[12,13,14], Geraldine Asselin[15], Anna Axelsson Raja[16], Joshua D. Backman[17], John Baksi[18], Paul J. R. Barton[19,20,18], Traci M. Bartz[21], Kiran J. Biddinger[7,9], Mary L. Biggs[21,22], Heather L. Bloom[23], Eric Boersma[24], Isabelle Bond[25], Jeffrey Brandimarto[26], Michael R. Brown[27], Søren Brunak[28], Hans-Peter Brunner-La Rocca[29], Mie Topholm Bruun[30], Rachel Buchan[19,20,18], Leonard Buckbinder[31], Henning Bundgaard[16], Douglas Cannie[25,32], Thomas P. Cappola[26], David J. Carey[33], Mark D. Chaffin[9], Philippe Charron[34,35], Daniel I. Chasman[36,37], Olympe Chazara[38], Xing Chen[31], Xu Chen[39], Jonathan H. Chung[17], William Chutkow[40], John G.F. Cleland[41,42], James P. Cook[43,44], Stuart A. Cook[19,20], Tomasz Czuba[45], Trégouët David-Alexandre[46,47], Simon de Denus[15,48], Antonio de Marvao[19,20], Abbas Dehghan[49], Graciela E. Delgado[50], Spiros Denaxas[51,12,52,13], Alexander S. Doney[53], Marcus Dörr[54,55], Joseph Dowsett[56], J. Gustav Smith[57,45,58], Marie-Pierre Dubé[15,59], Samuel C. Dudley[60], Michael E. Dunn[61], Patrick T. Ellinor[62,9], Perry Elliott[25], Gunnar Engström[4], Villard Eric[34,35], Christian Erikstrup[63,64], Tõnu Esko[2,9], Eric H. Farber-Eger[65], Ghazaleh Fatemifar[66,12], Stephan B. Felix[54,55], Chris Finan[25], Sarah Finer[67], Ian Ford[68], Francoise Fougerousse[69], René Fouodjio[15], Catherine Francis[19,18], Sophie Garnier[34,35], Mohsen Ghanbari[70], Sahar Ghasemi[71,55,72], Jonas Ghouse[73], Vilmantas Giedraitis[74], Franco Giulianini[36], John S. Gottdiener[75], Stefan Gross[54,55], Daníel F. Guðbjartsson[76,77], Hongsheng Gui[78], Karl Guo[79], Rebecca Gutmann[80], Sara Hägg[81], Christopher M. Haggerty[33], Brian Halliday[19,18], Åsa K. Hedman[82], Anna Helgadottir[76], Harry Hemingway[12,51], Albert Henry[25,12], Hans Hillege[83], Aroon D. Hingorani[84,25], Hilma Holm[76], Michael V. Holmes[85,86,87], Craig L. Hyde[31], Erik Ingelsson[88,89,90,91], Hanane Issa[12], Jaison Jacob[92], Deleuze Jean-François[93,47,94], J. Wouter Jukema[95,96], Frederick Kamanu[97,98], Isabella Kardys[24], Maryam Kavousi[70], Kay-Tee Khaw[99], Jorge R. Kizer[100], Marcus E. Kleber[50], Lars Køber[101], Andrea Koekemoer[102], Bill Kraus[103], Karoline Kuchenbaecker[104,105], Yi-Pin Lai[31], David Lanfear[78,106], Chim C. Lang[53], Claudia Langenberg[107,108,109], Michael Lee[19], Honghuang Lin[6,110], Lars Lind[111], Cecilia M. Lindgren[112,9,113], Barry London[114], Luca A. Lotta[115], Ruth C. Lovering[25], Brandon D. Lowery[65], Jian'an Luan[109], Steven A. Lubitz[62,9], R. Thomas Lumbers[13,51,12], Patrik Magnusson[81], Anubha Mahajan[113], Anders Malarstig[82,31], Charlotte Manisty[25,116], Douglass Mann[117], Kenneth B. Margulies[26], Nicholas A. Marston[118], Hilary Martin[119], Winfried März[120,121,50], Kathryn McGurk[19,20], John J.V. McMurray[122], Olle Melander[123], Giorgio Melloni[97,98], David Miller[124], James C Moon[125,25],

Ify R. Mordi[53], Thomas Morgan[40,126], Michael P. Morley[26], Andrew D. Morris[127], Andrew P. Morris[44,113], Alanna C. Morrison[27], Lori Morton[61], Michael W. Nagle[31], Christopher P. Nelson[102], Christopher Newton-Cheh[7,128,9], Alexander Niessner[129], Teemu Niiranen[130,131], Raymond Noordam[132], Michela Noseda[19], Mahdad Noursadeghi[133], Christoph Nowak[10], Michelle L. O'Donoghue[118], Declan P O'Regan[19,20], Sisse Rye Ostrowski[56,134], Anjali T. Owens[26], Colin N. A. Palmer[135], Antonis Pantazis[18], Guillaume Paré[136,137,138], Helen M. Parry[53], Lavinia Paternoster[139,140], Ole Birger Pedersen[141,134], Markus Perola[131], Louis-Philippe Lemieux Perreault[15], Marie Pigeyre[142,138], Eliana Portilla-Fernandez[70,143], Sanjay K. Prasad[19,18], Bruce M. Psaty[144,145], Kenneth M. Rice[21], Paul M. Ridker[36,37], Simon P. R. Romaine[102], Carolina Roselli[83,9], Jerome I. Rotter[146,147,148], Christian T. Ruff[118], Mark S. Sabatine[118], Neneh Sallah[12], Perttu Salo[131], Veikko Salomaa[131], Nilesh J. Samani[102], Naveed Sattar[149], Jessica van Setten[150], Sonia Shah[151,25], Svati Shah[152,153,103], Alaa A. Shalaby[154], Akshay Shekhar[61], Diane T. Smelser[33], Nicholas L. Smith[155,145,156], Erik Sørensen[157], Doug Speed[158], Sundararajan Srinivasan[135], Kari Stefansson[76,159], Steen Stender[160], David J. Stott[161], Nicholas P. Sunderland[162], Garðar Sveinbjörnsson[76], Per Svensson[163,164], Daniel I. Swerdlow[165,25], Petros Syrris[25], Mari-Liis Tammesoo[2], Jean-Claude Tardif[15,59], Upasana Tayal[19,18], Kent D. Taylor[166], Maris Teder-Laving[2], Alexander Teumer[55,167,72], Pantazis I Theotokis[20], Guðmundur Thorgeirsson[76,168,159], Unnur Thorsteinsdottir[76,159], Christian Torp-Pedersen[169], Vinicius Tragante[76], Thomas A Treibel[125,25], Stella Trompet[95,132], Danny Tuckwell[92], Benoit Tyl[170], Andre G. Uitterlinden[70,143], Henrik Ullum[171], Ana M Valdes[172], Pim van der Harst[83,150], David van Heel[173], Ramachandran S. Vasan[6,174], Felix Vaura[175,176], Abirami Veluchamy[53,135], Monique Verschuuren[177,178], Niek Verweij[83], Peter M. Visscher[179,180], Christoffer Rasmus Vissing[16], Uwe Völker[55,181], Adriaan A. Voors[83], Marion van Vugt[150], Lars Wallentin[182], Xiaosong Wang[40], Yunzhang Wang[81], James S. Ware[19,183,18,184,9], Nicholas J. Wareham[115], Dawn Waterworth[79], Peter E. Weeke[73], Raul Weiss[185], Quinn Wells[186], Harvey White[187], Kerri L. Wiggins[22], Jemma B. Wilk[31], L. Keoki Williams[78], Heming Xing[40], Xiao Xu[19,20], Jian Yang[179,180], Yifan Yang[26], Laura M. Yerges-Armstrong[79], Bing Yu[27], Faiez Zannad[188], Faye Zhao[92], Jing Hua Zhao[115], Chaoqun Zheng[16], Sean L. Zheng[19,20,18]

Affiliations

1. Department of Clinical Immunology, Aalborg Univeristy Hospital, Aalborg
2. Estonian Genome Center, Institute of Genomics, University of Tartu, Tartu, 51010, Estonia
3. Geisinger Health System, Danville, PA, USA
4. Department of Clinical Sciences, Lund University, Malmö, Sweden
5. Department of Cardiology, Herlev Gentofte Hospital, Herlev Ringvej 57, Herlev, 2650, Denmark
6. National Heart, Lung, and Blood Institute's and Boston University's Framingham Heart Study, Framingham, Massachusetts, USA

7. Cardiovascular Research Center, Massachusetts General Hospital, Boston, MA, USA
8. Center for Genomic Medicine, Massachusetts General Hospital, Boston, MA, USA
9. Program in Medical and Population Genetics, The Broad Institute of MIT and Harvard, Cambridge, MA, USA
10. Department of Neurobiology, Care Sciences and Society/ Section of Family Medicine and Primary Care, Karolinska Institutet, Stockholm, Sweden
11. School of Health and Social Sciences, Dalarna University, Falun, Sweden
12. Institute of Health Informatics, University College London, London, England, UK
13. The National Institute for Health Research University College London Hospitals Biomedical Research Centre, University College London, London, England, UK
14. Department of Cardiology, Amsterdam Cardiovascular Sciences, Amsterdam University Medical Centers, Amsterdam, The Netherlands
15. Montreal Heart Institute, Montreal, Canada
16. Department of Cardiology, The Heart Centre, Copenhagen University Hospital, Rigshospitalet, Ingen Lehmanns vej 7, Copenhagen, 2100, Denmark
17. Regeneron Genetics Center, 777 Old Saw Mill River Road, Tarrytown, NY 10591, USA
18. Royal Brompton & Harefield Hospitals, Guy's and St. Thomas' NHS Foundation Trust, London, England, UK
19. National Heart & Lung Institute, Imperial College London, London, England, UK
20. MRC London Institute of Medical Sciences, London, England, UK
21. Department of Biostatistics, University of Washington, Seattle, WA, USA
22. Department of Medicine, University of Washington, Seattle, WA, USA
23. Department of Medicine, Division of Cardiology, Emory University Medical Center, Atlanta, GA, USA
24. Department of Cardiology, Erasmus University Medical Center, Rotterdam, The Netherlands
25. Institute of Cardiovascular Science, University College London, London, England, UK
26. Penn Cardiovascular Institute, Perelman School of Medicine, University of Pennsylvania, Philadelphia, PA, USA
27. Department of Epidemiology, Human Genetics, and Environmental Sciences, The University of Texas School of Public Health, Houston, TX, 77030, USA
28. Novo Nordisk Foundation Center for Protein Research, Faculty of Health and Medical Sciences, University of Copenhagen, Copenhagen, Denmark
29. Maastricht University Medical Center, the Netherlands
30. Department of Clinical Immunology, Odense University Hospital, Odense, Denmark
31. Pfizer Worldwide Research & Development, 1 Portland St, Cambridge, MA, USA
32. London, England, UK
33. Department of Molecular and Functional Genomics, Geisinger, Danville, PA, USA

34. UMR-S1166, Research Unit on Cardiovascular Disorders, Metabolism and Nutrition, Team Genomics & Pathophysiology of Cardiovascular Diseases, Sorbonne Université, INSERM, UMR-S1166, Paris, 75013, France

35. ICAN Institute for Cardiometabolism and Nutrition, Paris, 75013, France

36. Division of Preventive Medicine, Brigham and Women's Hospital, Boston, MA, USA

37. Harvard Medical School, Boston, MA, USA

38. Centre for Genomics Research, Discovery Sciences, BioPharmaceuticals R&D, AstraZeneca, Cambridge, UK

39. Department of Medical Epidemiology and Biostatistics, Karolinska Institutet, Stockholm, Sweden.

40. Novartis Institutes for Biomedical Research

41. National Heart and Lung Institute, Imperial College, London, England, UK.

42. Robertson Centre for Biostatistics & Glasgow Clinical Trials Unit, Institute of Health and Wellbeing, University of Glasgow, Glasgow Royal Infirmary, Glasgow, UK

43. Department of Health Data Science, University of Liverpool, Liverpool, England, UK

44. Department of Biostatistics, University of Liverpool, Liverpool, England, UK

45. Department of Molecular and Clinical Medicine, Institute of Medicine, Gothenburg University and Sahlgrenska University Hospital, Gothenburg, Sweden

46. U1219, Univ. Bordeaux, INSERM, BPH, Bordeaux, 33000, France

47. Laboratory of Excellence GENMED (Medical Genomics), France

48. Faculty of Pharmacy, Université de Montréal, Montreal, Canada

49. MRC-PHE Centre for Environment and Health, Department of Epidemiology and Biostatistics, Imperial College London, London, England, UK

50. Vth Department of Medicine (Nephrology, Hypertensiology, Endocrinology, Diabetology, Rheumatology), Medical Faculty of Mannheim, University of Heidelberg, Heidelberg, Germany

51. Health Data Research UK, London, England, UK

52. British Heart Foundation Data Science Centre, London, England, UK

53. Division of Molecular & Clinical Medicine, University of Dundee, Ninewells Hospital and Medical School, Dundee, DD1 9SY, Scotland, UK

54. Department of Internal Medicine B, University Medicine Greifswald, Greifswald, Germany

55. DZHK (German Center for Cardiovascular Research), partner site Greifswald, Greifswald, Germany

56. Department of Clinical Immunology, Copenhagen University Hospital, Rigshospitalet, Copenhagen, Denmark

57. Department of Cardiology, Clinical Sciences, Lund University and Skåne University Hospital, Lund, Sweden

58. Wallenberg Center for Molecular Medicine and Lund University Diabetes Center, Lund University, Lund, Sweden

59. Faculty of Medicine, Université de Montréal, Montreal, Canada

60. Department of Medicine, Cardiovascular Division, University of Minnesota, Minneapolis, MN, USA

61. Cardiovascular Research, Regeneron Pharmaceuticals, Tarrytown, NY, USA
62. Cardiac Arrhythmia Service and Cardiovascular Research Center, Massachusetts General Hospital, Boston, MA, USA
63. Department of Clinical Immunology, Aarhus University Hospital, Aarhus, Denmark
64. Deparment of Clinical Medicine, Health, Aarhus University, Aarhus, Denmark
65. Vanderbilt Institute for Clinical and Translational Research, Vanderbilt University Medical Center, Nashville, TN, USA
66. Health Data Research UK London, University College London, England, UK
67. Centre for Primary Care and Public Health, Wolfson Institute of Population Health, Queen Mary University of London, London, E1 4NS, England, UK
68. Robertson Center for Biostatistics, Institute of Health and Wellbeing, University of Glasgow, Glasgow, Scotland, UK
69. Translational and Clinical Research, Servier Cardiovascular Center for Therapeutic Innovation, Suresnes, France
70. Department of Epidemiology, Erasmus University Medical Center, Rotterdam, The Netherlands
71. Institute of Genetic Epidemiology, Faculty of Medicine and Medical Center, University of Freiburg, Freiburg, Germany
72. Department of Psychiatry and Psychotherapy, University Medicine Greifswald, Greifswald, Germany
73. Department of Cardiology, Rigshospitalet, Copenhagen University Hospital, Denmark
74. Department of Public Health and Caring Sciences, Geriatrics, Uppsala, Sweden
75. Department of Medicine, Division of Cardiology, University of Maryland School of Medicine, Baltimore, MD, USA
76. deCODE genetics/Amgen Inc., Sturlugata 8, Reykjavik, 101, Iceland
77. School of Engineering and Natural Sciences, University of Iceland, Sæmundargata 2, Reykjavik, 101, Iceland
78. Center for Individualized and Genomic Medicine Research, Department of Internal Medicine, Henry Ford Hospital, Detroit, MI, USA
79. Human Genetics, GlaxoSmithKline, Collegeville, PA, USA
80. Division of Cardiovascular Medicine, University of Iowa Carver College of Medicine, Iowa City, IA, USA
81. Department of Medical Epidemiology and Biostatistics, Karolinska Institutet, Stockholm, Sweden
82. Cardiovascular Medicine unit, Department of Medicine Solna, Karolinska Institute, Stockholm, Sweden
83. Department of Cardiology, University Medical Center Groningen, University of Groningen, Groningen, The Netherlands
84. BHF Research Accelerator, University College London, England, UK
85. Clinical Trial Service Unit and Epidemiological Studies Unit, Nuffield Department of Population Health, Big Data Institute, University of Oxford, Oxford, UK
86. Medical Research Council Population Health Research Unit at the University of Oxford, Oxford, UK

87. National Institute for Health Research Oxford Biomedical Research Centre, Oxford University Hospital, Oxford, UK
88. Department of Medical Sciences, Molecular Epidemiology and Science for Life Laboratory, Uppsala University, Uppsala, Sweden.
89. Department of Medicine, Division of Cardiovascular Medicine, Stanford University School of Medicine, Stanford, CA 94305
90. Stanford Cardiovascular Institute, Stanford University, Stanford, CA 94305
91. Stanford Diabetes Research Center, Stanford University, Stanford, CA 94305
92. Novartis Institutes for Biomedical Research, Cambridge, MA, USA
93. Centre National de Recherche en Génomique Humaine (CNRGH), Institut de Biologie François Jacob, CEA, Université Paris-Saclay, Evry, 91057, France
94. Centre d'Etude du Polymorphisme Humain, Fondation Jean Dausset, Paris, France
95. Department of Cardiology, Leiden University Medical Center, Leiden, The Netherlands
96. Einthoven Laboratory for Experimental Vascular Medicine, LUMC, Leiden, The Netherlands
97. Cardiovascular Disease Initiative, The Broad Institute of MIT and Harvard, Cambridge, MA, USA
98. TIMI Study Group, Division of Cardiovascular Medicine, Brigham and Women's Hospital, Harvard Medical School, Boston, MA, USA
99. Department of Public Health and Primary Care, University of Cambridge, Cambridge, CB2 0QQ, UK
100. Cardiology Section, San Francisco Veterans Affairs Health System, and Departments of Medicine, Epidemiology and Biostatistics, University of California San Francisco, CA, USA
101. Department of Cardiology, Nordsjaellands Hospital, Hilleroed, Copenhagen, Denmark
102. Department of Cardiovascular Sciences, University of Leicester and NIHR Leicester Biomedical Research Centre, Glenfield Hospital, Leicester, England, UK
103. Duke Molecular Physiology Institute, Durham, NC, USA
104. Division of Psychiatry, University College London, London, W1T 7NF, England, UK
105. UCL Genetics Institute, University College London, London, WC1E 6BT, England, UK
106. Heart and Vascular Institute, Henry Ford Hospital, Detroit, MI, USA
107. Precision Healthcare University Research Institute, Queen Mary University of London, London, England, UK
108. Computational Medicine, Berlin Institute of Health (BIH) at Charité - Universitätsmedizin Berlin, Berlin, Germany
109. MRC Epidemiology Unit, Institute of Metabolic Science, University of Cambridge, Cambridge, CB2 0QQ, England, UK
110. Department of Medicine, University of Massachusetts Chan Medical School, Worcester, Massachusetts, USA
111. Department of Medical Sciences, Uppsala University, Uppsala, Sweden
112. Big Data Institute at the Li Ka Shing Centre for Health Information and Discovery, University of Oxford, Oxford, England, UK
113. Wellcome Trust Centre for Human Genetics, University of Oxford, Oxford, England, UK

114. Division of Cardiovascular Medicine and Abboud Cardiovascular Research Center, University of Iowa, Iowa City, IA, USA
115. MRC Epidemiology Unit, Institute of Metabolic Science, University of Cambridge School of Clinical Medicine, Cambridge, CB2 0QQ, UK
116. St Bartholomew's Hospital, Barts Heart Centre, UK
117. Center for Cardiovascular Research, Division of Cardiology, Department of Medicine, Washington University School of Medicine, St. Louis, MO, USA
118. TIMI Study Group, Division of Cardiovascular Medicine, Brigham and Women's Hospital, Harvard Medical School, Cambridge, MA, USA
119. Wellcome Sanger Institute, Wellcome Genome Campus, Hinxton, CB10 1RQ, England, UK
120. Clinical Institute of Medical and Chemical Laboratory Diagnostics, Medical University of Graz, Graz, Austria
121. Synlab Academy, Synlab Holding Deutschland GmbH, Mannheim, Germany
122. BHF Cardiovascular Research Centre, University of Glasgow, Scotland, United Kingdom
123. Department of Internal Medicine, Clinical Sciences, Lund University and Skåne University Hospital, Malmö, Sweden
124. Division of Biosciences, University College London, London, England, UK
125. St Bartholomew's Hospital, Barts Heart Centre
126. Vanderbilt University School of Medicine
127. Usher Institute of Population Health Sciences and Informatics, University of Edinburgh, Edinburgh, Scotland, UK
128. Center for Human Genetic Research, Massachusetts General Hospital, Boston, MA, USA
129. Department of Internal Medicine II, Division of Cardiology, Medical University of Vienna, Vienna, Austria
130. Department of Medicine, Turku University Hospital and University of Turku, Turku, Finland
131. National Institute for Health and Welfare, Helsinki, Finland
132. Section of Gerontology and Geriatrics, Department of Internal Medicine, Leiden University Medical Center, Leiden, The Netherlands
133. National Heart and Lung Institute, Imperial College, London, England, UK
134. Department of Clinical Medicine, Faculty of Health and Medical Sciences, University of Copenhagen, Copenhagen, Denmark
135. Division of Population Health and Genomics, University of Dundee, Ninewells Hospital and Medical School, Dundee, DD1 9SY, Scotland, UK
136. Department of Pathology and Molecular Medicine, McMaster University, Hamilton, ON, Canada
137. Thrombosis and Atherosclerosis Research Institute, Hamilton, ON, Canada
138. Population Health Research Institute, Hamilton, ON, Canada
139. Medical Research Council Integrative Epidemiology Unit, Bristol Medical School, University of Bristol, Bristol, England, UK
140. Population Health Sciences, Bristol Medical School, University of Bristol, Bristol, England, UK

141. Department of Clinical Immunology, Zealand University Hospital, Køge, Denmark
142. Department of Medicine, McMaster University, Hamilton, ON, Canada
143. Department of Internal Medicine, Erasmus University Medical Center, Rotterdam, The Netherlands
144. Cardiovascular Health Research Unit, University of Washington, Seattle, WA, USA
145. Kaiser Permanente Washington Health Research Institute, Kaiser Permanente Washington, Seattle, WA, USA
146. The Institute for Translational Genomics and Population Sciences, Harbor-UCLA Medical Center, Torrance, CA, USA
147. Departments of Pediatrics and Medicine, Harbor-UCLA Medical Center, Torrance, CA, USA
148. Los Angeles Biomedical Research Institute, Harbor-UCLA Medical Center, Torrance, CA, USA
149. BHF Cardiovascular Research Centre, University of Glasgow, Glasgow, Scotland, UK
150. Department of Cardiology, Division of Heart and Lungs, University Medical Center Utrecht, Utrecht, 3584 CX, The Netherlands
151. Institute for Molecular Bioscience, The University of Queensland, Brisbane, Australia
152. Division of Cardiology, Department of Medicine, Duke University Medical Center, Durham, NC, USA
153. Duke Clinical Research Institute, Durham, NC, USA
154. Department of Medicine, Division of Cardiology, University of Pittsburgh Medical Center and VA Pittsburgh HCS, Pittsburgh, PA, USA
155. Department of Epidemiology, University of Washington, Seattle, WA, USA
156. Seattle Epidemiologic Research and Information Center, Department of Veterans Affairs Office of Research & Development, Seattle, WA, USA
157. Department of Clinical Immunology, Copenhagen University Hospital, Rigshospitalet, Blegdamsvej 9, Copenhagen, Denmark
158. Quantitative Genetics and Genomics, Aarhus University, Aarhus, Denmark
159. Department of Medicine, University of Iceland, Sæmundargata 2, Reykjavik, 101, Iceland
160. Department of Clinical Biochemistry, Copenhagen University Hospital, Herlev and Gentofte, Denmark
161. Institute of Cardiovascular and Medical Sciences, College of Medical, Veterinary and Life Sciences, University of Glasgow, United Kingdom
162. Bristol Heart Institute / University Hospitals Bristol & Weston, University of Bristol, Bristol, England, UK
163. Department of Cardiology, Söderjukhuset, Stockholm, Sweden
164. Department of Clinical Science and Education-Södersjukhuset, Karolinska Institutet, Stockholm, Sweden
165. BenevolentAI, London, England, UK
166. Institute for Translational Genomics and Population Sciences, LABiomed and Departments of Pediatrics at Harbor-UCLA Medical Center, Torrance, Calif., USA 90502
167. Institute for Community Medicine, University Medicine Greifswald, Greifswald, Germany

168. Department of Internal Medicine, Division of Cardiology, National University Hospital of Iceland, Eiríksgata 5, Reykjavik, 101, Iceland
169. Department of Epidemiology and Biostatistics, Aalborg University Hospital, Aalborg, Denmark
170. Translational and Clinical Research, Servier Cardiovascular Center for Therapeutic Innovation, 50 rue Carnot 92284 Suresnes, France
171. Statens Serum Institut, Artillerivej 5, Copenhagen, 2300, Denmark
172. Injury, Recovery and Inflammation Sciences, School of Medicine, Nottingham, UK
173. Centre for Genomics and Child Health, Blizard Institute, Queen Mary University of London, London, E1 4AT, England, UK
174. Sections of Cardiology, Preventive Medicine and Epidemiology, Department of Medicine, Boston University Schools of Medicine and Public Health, Boston, Massachusetts, USA
175. Department of Clinical Medicine, University of Turku, Turku, Finland
176. Finnish Institute for Health and Welfare, Helsinki, Finland
177. Department Life Course and Health, Centre for Nutrition, Prevention and Health Services, National Institute for Public Health and the Environment, Bilthoven, The Netherlands
178. Julius Center for Health Sciences and Primary Care, University Medical Center Utrecht, Utrecht, The Netherlands
179. Institute for Molecular Bioscience, The University of Queensland, Brisbane, Queensland 4072, Australia
180. Queensland Brain Institute, The University of Queensland, Brisbane, Queensland 4072, Australia
181. Interfaculty Institute for Genetics and Functional Genomics, University Medicine Greifswald, Greifswald, Germany
182. Uppsala Clinical Research Center, Uppsala University, Uppsala, Sweden
183. MRC London Institute of Medical Sciences, Imperial College London, London, England, UK
184. Hammersmith Hospital, Imperial College Hospitals NHS Trust, London, England, UK
185. Department of Internal Medicine, Division of Cardiovascular Medicine, The Ohio State University Medical Center, Columbus, OH, USA
186. Division of Cardiovascular Medicine, Vanderbilt University, Nashville, TN, USA
187. Green Lane Cardiovascular Service, Auckland City Hospital, USA
188. Université de Lorraine, CHU de Nancy, Inserm and INI-CRCT (F-CRIN), Institut Lorrain du Coeur et des Vaisseaux, Vandoeuvre Lès Nancy, 54500, France

MVP banner authors

MVP Program Office

- Sumitra Muralidhar, Ph.D., Program Director
US Department of Veterans Affairs, 810 Vermont Avenue NW, Washington, DC 20420
- Jennifer Moser, Ph.D., Associate Director, Scientific Programs
US Department of Veterans Affairs, 810 Vermont Avenue NW, Washington, DC 20420
- Jennifer E. Deen, B.S., Associate Director, Cohort & Public Relations
US Department of Veterans Affairs, 810 Vermont Avenue NW, Washington, DC 20420

MVP Executive Committee

- Co-Chair: Philip S. Tsao, Ph.D.
VA Palo Alto Health Care System, 3801 Miranda Avenue, Palo Alto, CA 94304
- Co-Chair: Sumitra Muralidhar, Ph.D.
US Department of Veterans Affairs, 810 Vermont Avenue NW, Washington, DC 20420
- J. Michael Gaziano, M.D., M.P.H.
VA Boston Healthcare System, 150 S. Huntington Avenue, Boston, MA 02130
- Elizabeth Hauser, Ph.D.
Durham VA Medical Center, 508 Fulton Street, Durham, NC 27705
- Amy Kilbourne, Ph.D., M.P.H.
VA HSR&D, 2215 Fuller Road, Ann Arbor, MI 48105
- Michael Matheny, M.D., M.S., M.P.H.
VA Tennessee Valley Healthcare System, 1310 24th Ave. South, Nashville, TN 37212
- Dave Oslin, M.D.
Philadelphia VA Medical Center, 3900 Woodland Avenue, Philadelphia, PA 19104

MVP Co-Principal Investigators

- J. Michael Gaziano, M.D., M.P.H.
VA Boston Healthcare System, 150 S. Huntington Avenue, Boston, MA 02130
- Philip S. Tsao, Ph.D.
VA Palo Alto Health Care System, 3801 Miranda Avenue, Palo Alto, CA 94304

MVP Core Operations

- Jessica V. Brewer, M.P.H., Director, MVP Cohort Operations
VA Boston Healthcare System, 150 S. Huntington Avenue, Boston, MA 02130
- Mary T. Brophy M.D., M.P.H., Director, VA Central Biorepository
VA Boston Healthcare System, 150 S. Huntington Avenue, Boston, MA 02130
- Kelly Cho, M.P.H, Ph.D., Director, MVP Phenomics
VA Boston Healthcare System, 150 S. Huntington Avenue, Boston, MA 02130
- Lori Churby, B.S., Director, MVP Regulatory Affairs

VA Palo Alto Health Care System, 3801 Miranda Avenue, Palo Alto, CA 94304
- Scott L. DuVall, Ph.D., Director, VA Informatics and Computing Infrastructure (VINCI)
VA Salt Lake City Health Care System, 500 Foothill Drive, Salt Lake City, UT 84148
- Saiju Pyarajan Ph.D., Director, Data and Computational Sciences
VA Boston Healthcare System, 150 S. Huntington Avenue, Boston, MA 02130
- Robert Ringer, Pharm.D., Director, VA Albuquerque Central Biorepository
New Mexico VA Health Care System, 1501 San Pedro Drive SE, Albuquerque, NM 87108
- Luis E. Selva, Ph.D., Director, MVP Biorepository Coordination
VA Boston Healthcare System, 150 S. Huntington Avenue, Boston, MA 02130
- Shahpoor (Alex) Shayan, M.S., Director, MVP PRE Informatics
VA Boston Healthcare System, 150 S. Huntington Avenue, Boston, MA 02130
- Brady Stephens, M.S., Principal Investigator, MVP Information Center
Canandaigua VA Medical Center, 400 Fort Hill Avenue, Canandaigua, NY 14424
- Stacey B. Whitbourne, Ph.D., Director, MVP Cohort Development and Management
VA Boston Healthcare System, 150 S. Huntington Avenue, Boston, MA 02130

MVP Publications and Presentations Committee

- Co-Chair: Themistocles L. Assimes, M.D., Ph. D
VA Palo Alto Health Care System, 3801 Miranda Avenue, Palo Alto, CA 94304
- Co-Chair: Adriana Hung, M.D.; M.P.H
VA Tennessee Valley Healthcare System, 1310 24th Ave. South, Nashville, TN 37212
- Co-Chair: Henry Kranzler, M.D.
Philadelphia VA Medical Center, 3900 Woodland Avenue, Philadelphia, PA 19104

Cell type enrichment analysis using the Chaffin et al. snRNAseq dataset

Using the snRNA-seq data obtained from Chaffin et al., 2022 (ref.[41]), we performed several analyses focused on cell type enrichment. The dataset consisted of LV samples from 11 DCM patients, 16 non-failing controls and 15 HCM patients. In terms of analyses, we i) generated cell type-specific annotations for enrichment testing using stratified linkage disequilibrium score regression (s-LDSC)[42] and ii) generated 'disease-dependent' cell type annotations for enrichment testing using s-LDSC.

Cell type specific gene programs

Based on the Chaffin et al. dataset, we defined cell type-specific gene expression profiles by collapsing nuclei into 17 major cell types from the human left ventricle. We then identified differentially expressed genes in each cell type compared to all other cell types. To control for the inherent correlation of nuclei from the same individual, we created a pseudo-bulk expression profile after summing gene expression counts across all nuclei for each combination of individual and cell type. Individual and cell type combinations with fewer than 50 nuclei were omitted and lowly expressed genes were removed using the function filterByExpr() in edgeR[43]. Gene expression was normalized with DESeq2[44] and differential expression testing was performed using limma-voom[45]. Using a design matrix ~0 + cell_type + individual, we extracted an explicit contrast comparing expression in each cell type to all other cell types. For each cell type, we defined the cell type-specific profile as the top 10% most upregulated genes based on the t-statistic from the differential expression test.

s-LDSC analysis of cell type specific gene programs

We annotated SNPs within a 100 Kb window on either side of the transcribed region for each set of cell type specific genes, as in Finucane et al, 2018 (ref.[46]). Gene coordinates were based on the GRCh38 gene reference used in the snRNAseq data analysis. Using these annotations, we tested for cell type enrichment using s-LDSC, controlling for an annotation derived from all genes tested for differential expression and the baseline annotations from Finucane et al., 2015 (ref.[42]). As recommended, we report one-sided *P*-values from the tau 'coefficient' - which is conditional on all other annotations included in the model - and not the 'enrichment' statistic. As LD reference, we used the previously derived 1000 Genomes European ancestry LD reference provided with the software. To account for the 17 cell types tested for GWAS-DCM and MTAG-DCM, we applied a Bonferroni significance cutoff by setting significance at 0.05/17=0.0029.

s-LDSC analysis of disease-dependent gene programs

As described below for the Reichart dataset, we also performed an analysis of disease-dependent gene programs using the Chaffin et al. dataset (ref.[41]). We took the results from the differential expression analysis as described previously[41] (using CellBender-adjusted expression counts), and considered genes with $|\log FC| > 0.5$ and an FDR-adjusted $P < 0.05$ as 'disease-dependent' genes in the given cell type. We annotated SNPs within +/-100KB from each gene identified for each cell type and ran s-LDSC to identify GWAS heritability enrichment of these annotations, adjusting for baseline annotations from Finucane et al. 2015 (ref.[42]) and a set of annotations derived from all genes tested for differential expression in the given cell type. As recommended by Finucane et al., we report test statistics and corresponding *P*-values from the tau 'coefficient' - which is conditional on all other annotations in the model - and not the 'enrichment' statistic (which is not conditional on the other annotations).

Cell type enrichment and differential expression analyses in the Reichart et al. snRNAseq dataset

Using the snRNA-seq data obtained from Reichart et al., 2022 (ref.[47]), we performed several analyses focused on cell type enrichment and differential expression. The dataset consisted of samples from several anatomical locations (including several locations across the left and right ventricle) from 61 cardiomyopathy patients - of which 52 with DCM - and 18 non-failing controls. In terms of analyses, we i) generated cell type-specific annotations for enrichment testing using stratified linkage disequilibrium score regression (s-LDSC)[42], ii) generated differential expression data comparing left ventricles from DCM patients with non-failing control left ventricles, and iii) generated ‘disease-dependent’ cell type annotations for enrichment testing using s-LDSC.

Cell type specific gene programs

First, to test for enrichment of cell type specific gene programs in our GWAS/MTAG data, we generated a list of cell type specific genes. We removed nuclei labeled as ‘native’ or ‘lowQC’ prior to estimating cell type specific genes. We then performed ‘pseudo-bulk’ aggregation by summing gene counts across nuclei for each donor/tissue region combination, by cell type. We only retained a given donor/tissue region combination if they had at least 50 nuclei of that cell type. Lowly expressed genes identified with the filterByExpr() function in edgeR were removed. We normalized the pseudo-bulk expression with DESeq2 and fit the differential expression model ~0+cell_type+donor_tissue using limma-voom. Notably, we included a covariate for the donor/tissue region combination because each donor/tissue region will be represented across most cell types. We then extracted contrasts comparing gene expression in each focal cell type to all other cell types.

s-LDSC analysis of cell type specific gene programs

To generate annotations for s-LDSC, we sorted all genes tested for each cell type by t-statistic and selected the top 10% of genes to represent each cell type, as in Finucane et al, 2018 (ref.[46]). We annotated any SNP within +/-100KB of the genes for each cell type as 'cell type specific' SNPs. Using these annotations, we tested for cell type enrichment using s-LDSC, controlling for an annotation derived from all genes tested for differential expression and the baseline annotations from Finucane et al., 2015 (ref.[42]). As recommended by Finucane et al., we report test statistics and corresponding one-sided *P*-values from the tau 'coefficient' - which is conditional on all other annotations included in the model - and not the 'enrichment' statistic (which is not conditional on other annotations). To account for the 9 cell types tested for GWAS-DCM and MTAG-DCM, we applied a Bonferroni significance cutoff by setting significance at 0.05/9=0.0.0056.

Differential expression analysis of DCM versus controls

Second, we generated a list of differentially expressed genes between dilated cardiomyopathy (DCM) cases and normal controls by cell type. We first restricted our analysis to samples from the left ventricle (LV) and removed any nuclei flagged as low quality. Next, for a given cell type, we summed transcriptional counts across all nuclei from each donor of origin. Of note, we only generated a 'pseudo-bulk' profile for a donor if they had more than 20 nuclei of the given cell type. We then removed mitochondrial genes, ribosomal genes, and any gene that was found in <1% of nuclei from both DCM nuclei and control nuclei. We further removed lowly expressed genes using the function filterByExpr() from edgeR. We normalized the expression data using DESeq2 normalization, and then tested for differential expression between DCM cases (N_{max}=52) and non-

failing controls (N_{max}=18) using limma-voom with the model of ~1 + disease + sex. Multiple testing correction was performed using the Benjamini-Hochberg procedure.

s-LDSC analysis of disease-dependent gene programs

In contrast to the cell type specific gene programs defined by high cell type specificity of expression, we then also generated 'disease-dependent' gene programs for cell types. Disease-dependent gene programs consist of genes that are differentially expressed between the disease state and the healthy state, and therefore may consist partly of genes that are not expressed to a high degree in the given cell type or may not be cell type-specific. Such programs may capture disease-response mechanisms, rather than disease initiation mechanisms[48]. To generate disease-dependent cell type annotations of s-LDSC, we used the results from the differential expression analysis described above, and considered genes with |logFC| > 0.5 and an FDR-adjusted $P < 0.05$ as 'disease-dependent' genes in the given cell type. Of note, only 3 genes were identified in adipocytes with this procedure, and therefore we excluded adipocytes for the s-LDSC analysis. We annotated SNPs within +/-100KB from each gene identified for each cell type and ran s-LDSC to identify GWAS heritability enrichment of these annotations, adjusting for baseline annotations from Finucane et al. 2015 (ref.[42]) and a set of annotations derived from all genes tested for differential expression in the given cell type. As recommended by Finucane et al., we report test statistics and corresponding *P*-values from the tau 'coefficient' - which is conditional on all other annotations in the model - and not the 'enrichment' statistic (which is not conditional on the other annotations).

Harmonization of cell types across single cell datasets to construct LV expression patterns

We used three single cell datasets of heart to construct expression patterns for genes identified from our GWAS-DCM and MTAG-DCM. These datasets included Chaffin et al.[41] , Reichart et al.[47] , and Koenig et al.[49]. To harmonize cell type data across datasets, we used the available cell type and/or cell state annotations to collapse or split cell types into 'harmonized' cell types. In the Reichart dataset, nuclei with cell state 'PC1', 'PC2' or 'PC3' were collapsed into 'Pericytes'; nuclei with cell state 'SMC1.1', 'SMC1.2', or 'SMC2' were collapsed into 'VSMC'; nuclei with cell state 'EC7' were assigned 'Endocardial'; nuclei with cell state 'Meso' were assigned 'Epicardial'; nuclei with cell state 'EC8' were assigned 'Lymphatic endothelial'; nuclei with cell state 'EC1.0', 'EC2.0', 'EC5.0', or 'EC6.0' were assigned 'Cardiac endothelial'. In the Koenig dataset, cells/nuclei with cell type 'NK/T Cells' or 'B Cells' were collapsed into 'Lymphocyte'. In the Chaffin dataset, 'Cardiomyocyte_I', 'Cardiomyocyte_II', and 'Cardiomyocyte_III' were collapsed into 'Cardiomyocyte'; 'Endothelial_I', 'Endothelial_II', and 'Endothelial_III' were collapsed into 'Cardiac Endothelial'; 'Fibroblast_I', 'Fibroblast_II' and 'Activated_fibroblast' were collapsed into 'Fibroblast'; 'Pericyte_I' and 'Pericyte_II' were collapsed into 'Pericyte'; and 'Macrophage' and 'Proliferating_macrophage' were collapsed into 'Myeloid'.

Comparison with results from Zheng et al.

Genes prioritized in overlapping loci

Similar to our study, Zheng et al. performed a GWAS and MTAG for DCM, followed by gene prioritization through integration of several lines of evidence[33]. Of our 38 significant loci in GWAS-DCM, 20 overlapped genome-wide significant loci from GWAS for NICM/DCM reported by Zheng et al., while a total of 27 overlapped loci reported by the authors at more inclusive discovery thresholds (ie, DCM-Broad analyses at FDR1%, DCM-Strict analyses at genome-wide significance, or MTAG analyses at genome-wide significance[33]). Details on locus overlap is described in **Supplementary Table 39**. Across the 27 overlapping loci, gene prioritization from both studies nominated the exact same gene as the most likely causal gene in ~67% of the time, while ~19% of loci were partially concordant (ie, Zheng et al. described multiple genes with equal prio scores, one of which was concordant with our prioritized gene), and ~15% of loci were discordant. Of note, this intersection analysis considers all loci, even those with no gene highly-prioritized by our definitions. Therefore, we then restricted the comparison to loci with highly prioritized genes in both studies (ie, >=2.5 points and prioritized in our study AND >=3 points in Zheng et al. without ties). Strikingly, among 16 overlapping loci with 'strong prioritization' in both studies, the nominated gene was concordant in 94% of the time; only one locus was discordant (with *CRIM1* prioritized in our study and *STRN* prioritized in Zheng et al; **Supplementary Table 39**). When focusing on the 65 loci from our MTAG-DCM, 46 overlapped any of the significant loci from Zheng et al., with similar convergence of prioritization. Of all overlapping loci, ~72% nominated the same causal gene, ~13% showed partial concordance, and in ~15% of loci the most strongly prioritized gene differed between the two studies (**Supplementary Table 40**). More importantly, when restricting to loci where both studies strongly prioritized a gene, ~96% were concordant (again, only the *CRIM1* locus was discordant).

Interestingly, we note that both *CRIM1* and *STRN* are differentially expressed across several cell types in the single cell comparison of DCM LVs versus non-failing LVs. Furthermore, in our analyses (in both GWAS-DCM and MTAG-DCM) we identified two lead variants in this locus, of which one closer to *CRIM1* and one closer to *STRN*. These findings entertain the possibility that both genes have a causal role in DCM biology, although this would require functional validation.

Overall, the locus comparison results highlight a strong consistency in gene prioritization between our study and Zheng et al., in particular for genes identified with high prioritization scores.

Cell type enrichments

In the current study, we identified significant enrichment for DCM heritability only in cardiomyocyte gene programs. Zheng et al. additionally reported significant enrichment for several other cell types (eg. fibroblasts, mural cells). To understand the source of these discrepancies, we compared the similarities and differences between the two studies in more detail. Similar to our study, Zheng et al. performed cell type enrichment analyses by integrating results from their DCM GWAS data with snRNAseq data of the heart[33]. The authors re-processes the Reichart dataset[47] to serve as their expression set, and utilized an analytical pipeline similar to our cell type enrichment pipeline. Using cell type-specific gene programs - similar to our findings - the authors report significant enrichment of DCM heritability only in cardiomyocytes[33]. In contrast, the authors additionally report significant enrichment for several other cell types (eg. fibroblasts, mural cells) when using ‘disease-dependent’ gene programs. In disease-dependent gene program analyses (details on methods described in a previous Supplementary Note above), we did not uncover robust enrichments for any cell type at Bonferroni significance. At nominal significance, only

cardiomyocytes showed a (weak) consistent signal (*P*=0.04 in the Reichart dataset with positive coefficient in the Chaffin dataset).

Initially, we considered several potential explanations for this discrepancy. First, the cases included in the GWAS by Zheng et al. were only partially overlapping with our cases (including up to ~10k non-overlapping cases included in their effort). Perhaps more importantly, several of the included cohorts in Zheng et al. utilized a wider case definition - ie, any systolic dysfunction in absence of secondary causes. As such, the underlying GWAS data may have been inherently different between both studies. Nevertheless, we should note that the top loci from both studies show strong convergence, and the genetic correlations with cardiac endophenotypes were comparable between both efforts. For these reasons, we considered it less likely that the differences in the underlying GWAS data entirely explained the divergent cell type enrichment results, it may have contributed to an extent.

Second, we considered that differences in the construction of 'disease-dependent' gene programs from the snRNAseq data may have caused different results. Analytically, the approaches between both studies were highly similar. Zheng et al. used the Reichart dataset[47], which was also one of the two datasets used in our study. The authors used a similar pipeline to define 'disease-dependent' gene programs - including similar DCM/non-failing sample definitions, use of pseudo-bulking for DE-testing, and similar cutoffs for logFC and *P*-value in DE testing. One difference was that Zheng et al. re-processed the expression counts using CellBender to remove potentially remaining background noise, while we used the counts as provided by Reichart et al. We nevertheless note that CellBender was used to adjust count data in the Chaffin dataset[41] - where we also did not identify any significant enrichments for disease-dependent programs in our analyses.

Third, we considered that analytical differences in the statistical enrichment pipeline may have caused different results. Overall, the enrichment pipelines between both studies were reasonably comparable. Zheng et al. used parts of the sc-linker pipeline to perform their analyses[48]; sc-linker uses s-LDSC for enrichment testing[46], which is also the tool used by us for enrichment testing of cell type programs. We note that sc-linker uses activity-by-contact (ABC) mapping to link genes to genomic regions[48], while we used a more simple approach based on close proximity to gene bodies[46]. We note, however, that our approach yielded similar - or even stronger - enrichments for cell type-specific cardiomyocyte programs, which would indicate that this technical difference needn't be substantial. Nevertheless, as compared to cell type-specific gene programs, it is possible that ABC mapping is more important for disease-dependent programs (for which genes may be more distally regulated). Overall, the genomic mapping approach may have contributed to some extent to the different cell type enrichment results.

Critically, we found that Zheng et al. used a different statistic for hypothesis testing than used in our work. Specifically, the authors reported the 'enrichment' statistic or *Ec*. In contrast, we performed all hypothesis testing based on the 'enrichment coefficient' or *Tau_C*. When using the enrichment statistic instead of the coefficient, we recapitulate many of the significant findings reported in Zheng et al., including a pattern where a large proportion of disease-dependent gene programs reach nominal $P<0.05$ (**Supplementary Figure 13**). Within the s-LDSC and sc-linker frameworks, there are 4 major output statistics that involve/describe enrichment of heritability[46,48]. The simplest is the 'enrichment' or *Ec* statistic, which is the proportion of total heritability captured by the functional annotation of interest, divided by the proportion of SNPs included in the given annotation; this statistic is not conditional on other annotations/features fed into the s-LDSC model. The 'coefficient' or *Tau_C* is the regression coefficient from s-LDSC, which captures an 'adjusted' enrichment parameter conditional on the other annotations fed into s-LDSC. In our work, all coefficients are conditional on the baseline model (which incorporates annotations for

functional regions, including coding regions, enhancer regions, UTRs, etc); additionally, for disease-dependent programs, we included an annotation for all genes that could be assessed in differential expression testing (to account for the correct background of genes in the tissue). A third s-LDSC statistic is the *Tau_C** , which is simply a re-scaled standardized *Tau_C* statistic to represent an effect size per standard deviation of the underlying annotation. The final enrichment statistic is the 'enrichment score' or *E-score*, which was newly proposed as part of the sc-linker framework[46]. *E-score* essentially represents the difference between the *Ec* statistic for a given annotation and the background enrichment of all protein-coding genes with the relevant genomic-mapping in the given tissue. For cell type enrichment, the developers of s-LDSC previously recommended using *Tau_C* (or *Tau_C**) conditional on at least the baseline model for hypothesis testing[42,46], as this statistic corrects for the inherent enrichment of important genomic regions one might expect in GWAS. Since the publication of sc-linker, the developers recommend using *E-score* as an alternative[48], since it is corrected for the background of protein-coding regions while potentially yielding more power than *Tau_C*. Overall, one might conclude that *Ec* is the most 'liberal' statistic for enrichment testing (although prone to inflated type 1 error in cell type analyses; ref.[42]), while *Tau_C* is the most 'conservative' statistic when conditioned on the baseline model and an appropriate background of genes[42]. In their study, Zheng et al. used the 'enrichment' statistic or *Ec*, and we could indeed recapitulate several of their findings by performing hypothesis testing on *Ec* (**Supplementary Figure 13**). Importantly, these enrichments could not be recapitulated by us when conditioning on the baseline model and the appropriate background of genes (ie, when using *Tau_C*).

Taken together, the differing results from cell type enrichment analyses - of disease-dependent gene programs - may be partly explained by the reporting of a different enrichment statistic. Nevertheless, other technical differences likely contributed to some extent too - including a slightly different phenotype in GWAS and the use of a more simple genomic mapping approach in our

work. In all, across the GWAS studies, consistent evidence was found only for cardiomyocytes. For these reasons, we recommend that - outside of cardiomyocytes - enrichments in other cell types should be treated as interesting, but preliminary, at this stage.

Polygenic score prediction

Similar to our study, Zheng et al. report strong prediction of DCM using a PRS constructed from their GWAS. The authors tested their PRS in the UK Biobank, and reported prediction effect sizes of 1.76 OR increment per SD of PRS (95% CI 1.64 to 1.90). To more directly compare results, we then also tested our PRS within the UK Biobank, using the same dataset described in our GWAS and further restricting to samples with i) high-quality exome sequencing and genotyping array data available, ii) European genetic ancestry[50], iii) who were not related at a third degree or closer, and iv) who were not included within the first 45k participants with cardiac MRI data (since these samples contributed to the MTAG analyses). This procedure left 793 NI-DCM cases and 325313 controls. We then reran our main GWAS-DCM excluding UK Biobank, and constructed a new PRS using PRScs as described in our main methods[51]. Using this GWAS-DCM score (which was standardized to mean 0 and unit variance, and out of which the first 12 PCs were regressed), we then assessed the association with NI-DCM, adjusting for sex, age, age^2, PC1-12 and the genotyping array. Similarly, we re-ran the MTAG analysis using the GWAS-DCM[exclUKB] as base GWAS, and created MTAG-DCM[exclUKB] scores. The GWAS-DCM[exclUKB] score was strongly associated with NI-DCM in this dataset, with an OR increment per SD of PRS of 1.64 (95%CI 1.53 to 1.76), as was our MTAG-DCM[exclUKB] score at an OR increment per SD of PRS of 1.91 (95%CI 1.78 to 2.05). To compare more directly with the Zheng et al. PRS, we then downloaded their scoring files from the PGS catalog (GWAS:https://www.pgscatalog.org/score/PGS004861/ and MTAG: https://www.pgscatalog.org/score/PGS004861/), and scored the same samples using both

scoring files. We found that the GWAS (OR per SD 1.61; 95%CI 1.53 to 1.76) and MTAG scores (OR per SD 1.83; 95%CI 1.70 to 1.98) from the authors did well in prediction of NI-DCM, although slightly less well than the scores from our GWAS and MTAG, respectively. Using other metrics for prediction accuracy - including the variance explained, the AUC, and the AUPRC - similar patterns were observed (**Supplementary Table 41**). We note that we observed a slightly larger effect size for the Zheng et al. scores than reported by the authors; we posit that this difference is a reflection of the more stringent phenotype definition (ie, NI-DCM as compared to 'any' DCM).

Therefore, within the UK Biobank, the Zheng et al. PRSs seem to perform somewhat less well than the PRSs constructed from our data - although the confidence intervals were still overlapping. We therefore additionally assessed the Zheng et al. PRSs in the European subset of the *All of Us* dataset and within the Amsterdam dataset. In these datasets, we found that our GWAS-DCM and MTAG-DCM scores consistently achieved higher effect sizes, AUCs, and variances explained than the GWAS and MTAG scores from Zheng et al., respectively (**Supplementary Table 41**). The only exception was for AUPRC values in the *All of Us* dataset, which were marginally higher for the Zheng et al. scores.

Overall, the above results show that both studies produce scores that strongly predict NI-DCM and can transfer to datasets from different countries. The slightly better prediction of our PRS - despite considerably smaller case numbers - might reflect the higher specificity of our underlying phenotype. This would be consistent with the larger number of significant loci identified in our study. Alternatively, we note that Zheng et al. used ~700k variants for their PRS (as per PGS catalog), while our PRS was built using ~1.1M variants. The higher genome coverage might have contributed somewhat to a better prediction power using our PRS. Taken together, both studies produce strongly predictive PRS for DCM, with our PRS showing slightly better prediction of DCM in European ancestry.

Causal consequences of DCM liability

In our Mendelian randomization (MR) screen, we identified two potentially causal consequences of DCM liability, namely heart failure (HF) and platelet volume. The potentially causal effect of DCM liability on platelet volume was disputed by our sensitivity analyses. In particular, the link did not reach significance when using MR-Egger regression (**Supplementary Table 27**). For these reasons, we posit that this link likely represents a false-positive. Of note, the potentially causal link between DCM liability and HF did pass all sensitivity analyses and filters. In particular, CAUSE identified a strong causal effect of DCM liability on HF risk (g=0.06, 95%CI [0.04, 0.09]; **Figure 5a**; **Supplementary Figure 11**). This finding might reflect that a subset of HF cases have DCM, or that DCM genetics is causative of systolic HF more broadly, as investigated further in our PRS analyses.

Supplementary Tables

The Supplementary Tables can be found in the accompanying Excel file.

Supplementary Figures and Figure Legend

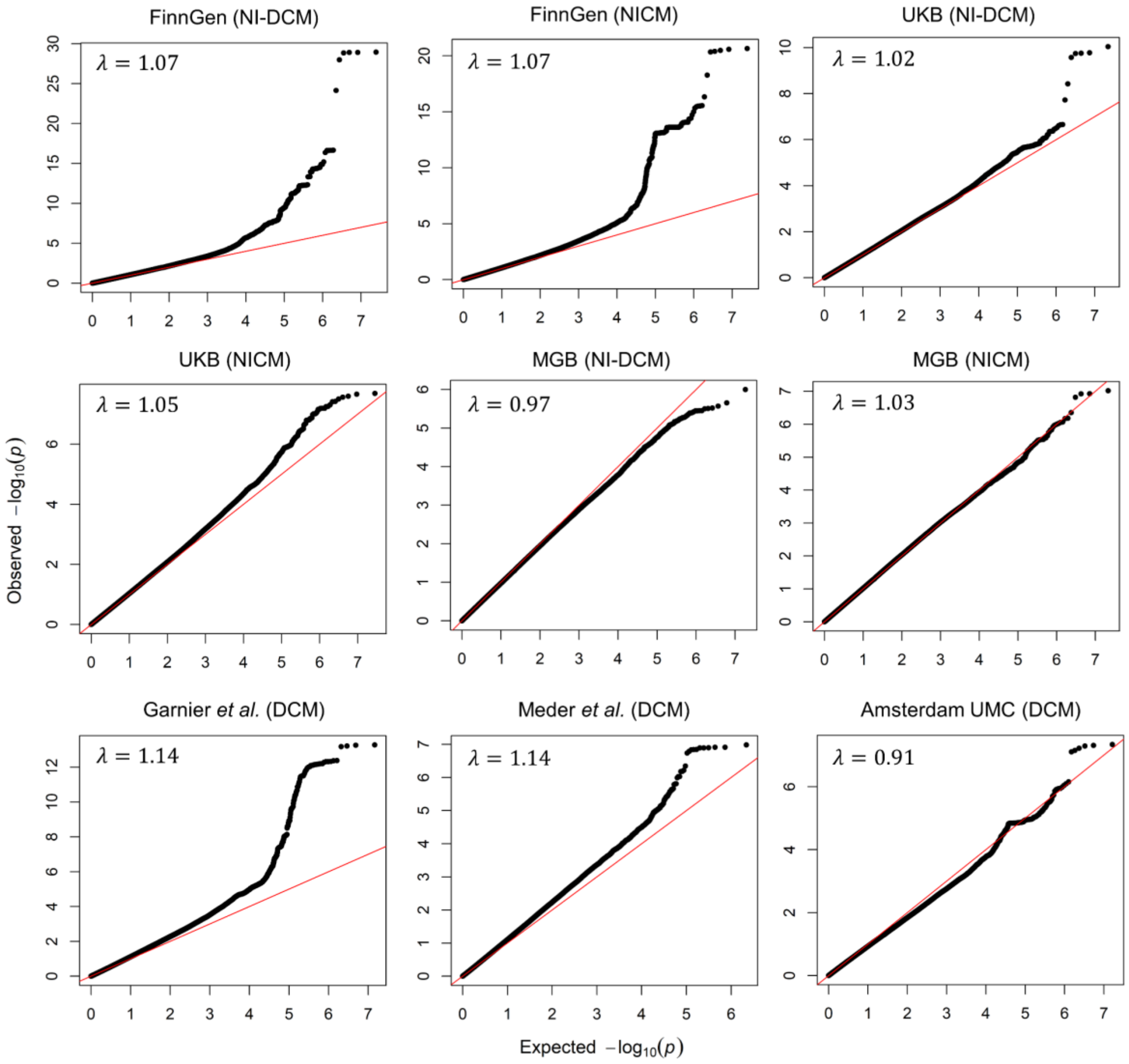

Supplementary Figure 1: Quantile-quantile plots of contributing studies to the meta-analyses of NI-DCM, NICM and clinical DCM.

Each panel shows the quantile-quantile plot for a given GWAS in a given dataset. In each quantile-quantile plot, the x-axis represents the expected -log10 of the *P*-value of variants under the null hypothesis, while the y-axis represents the observed -log10 of the *P*-value in the GWAS. The red line shows the expected calibration under the null hypothesis. The genomic inflation factor lambda (computed as the observed X^2 statistic at the median over the expected under the null) are shown in the top left of each panel, where the lambda was computed over all plotted variants. Variants are filtered to those that passed filters for inclusion into the overall

meta-analyses (**Methods**). *P*-values are derived from various logistic regression models; reported *P*-values are two-sided and unadjusted for multiple testing. Note: GWAS, genome-wide association study; NI-DCM, nonischemic dilated cardiomyopathy in a biobank dataset; NICM, nonischemic cardiomyopathy in a biobank dataset; DCM; dilated cardiomyopathy.

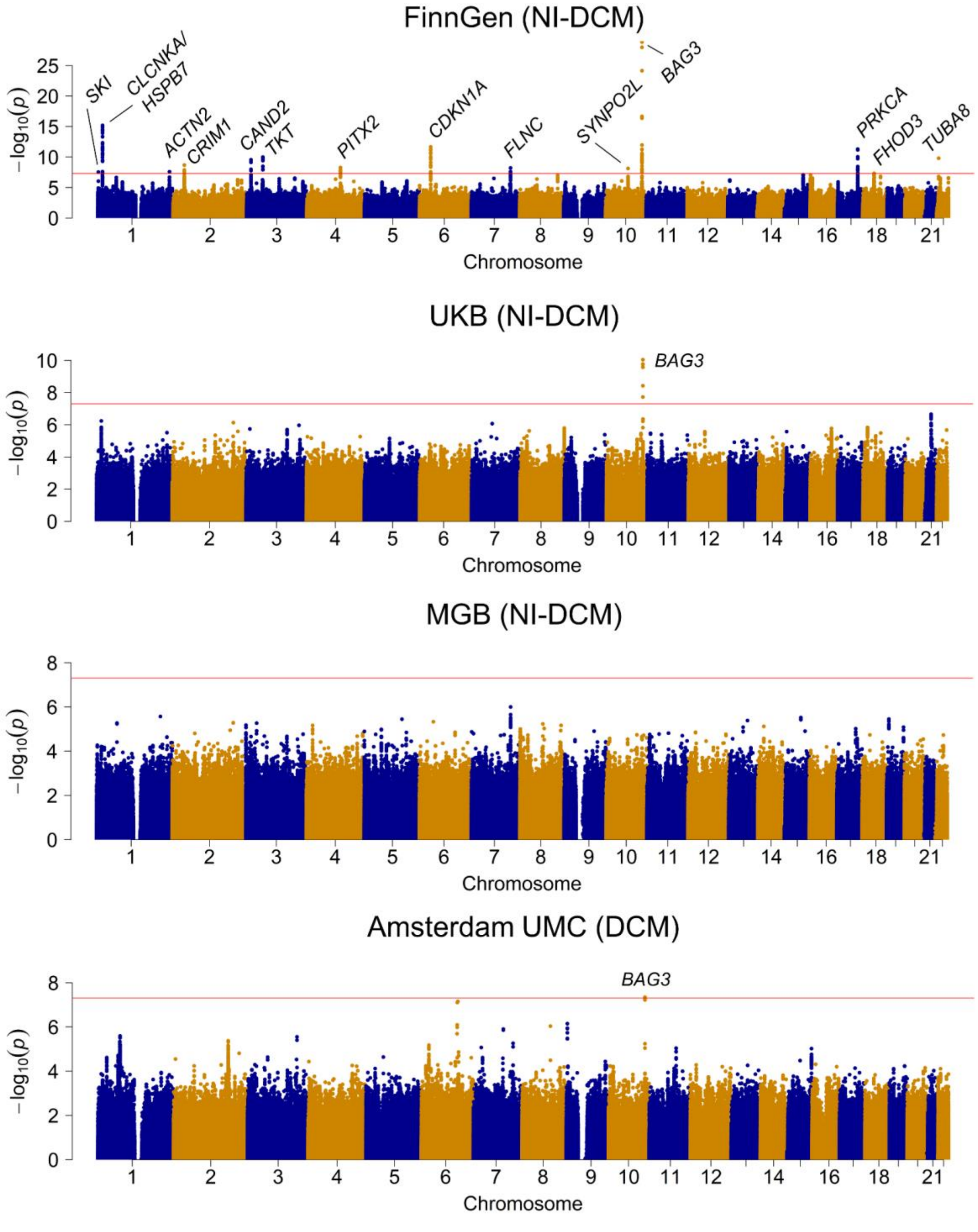

Supplementary Figure 2: Manhattan plots for biobank datasets and newly-analyzed clinical datasets for NI-DCM / clinical DCM

Each panel shows a Manhattan for GWAS of (NI-)DCM in a dataset, where each dot represents a single tested variant, the x-axis shows genomic coordinates for those variants (chromosome, and position on chromosome), while the y-axis shows the -log10 of the *P*-value from GWAS.

The red line indicates the conventional genome-wide significance level (alpha=5x10^{-8}). Loci reaching above the significance line are annotated with a gene name, where the annotated gene is harmonized with the locus name from our main GWAS (ie, highest prioritized gene in locus from GWAS-DCM/MTAG-DCM) for easy comparisons; sometimes an additional gene is highlighted to serve easier comparison to previously-published GWAS; if a locus was not identified in GWAS-DCM/MTAG-DCM, the closest protein-coding gene is used. Results here are from the biobank cohorts and from a newly analyzed clinical dataset (Amsterdam UMC dataset). *P*-values are derived from various logistic regression models; reported *P*-values are two-sided and unadjusted for multiple testing. Note: GWAS, genome-wide association study; NI-DCM, nonischemic dilated cardiomyopathy in a biobank dataset; DCM; dilated cardiomyopathy.

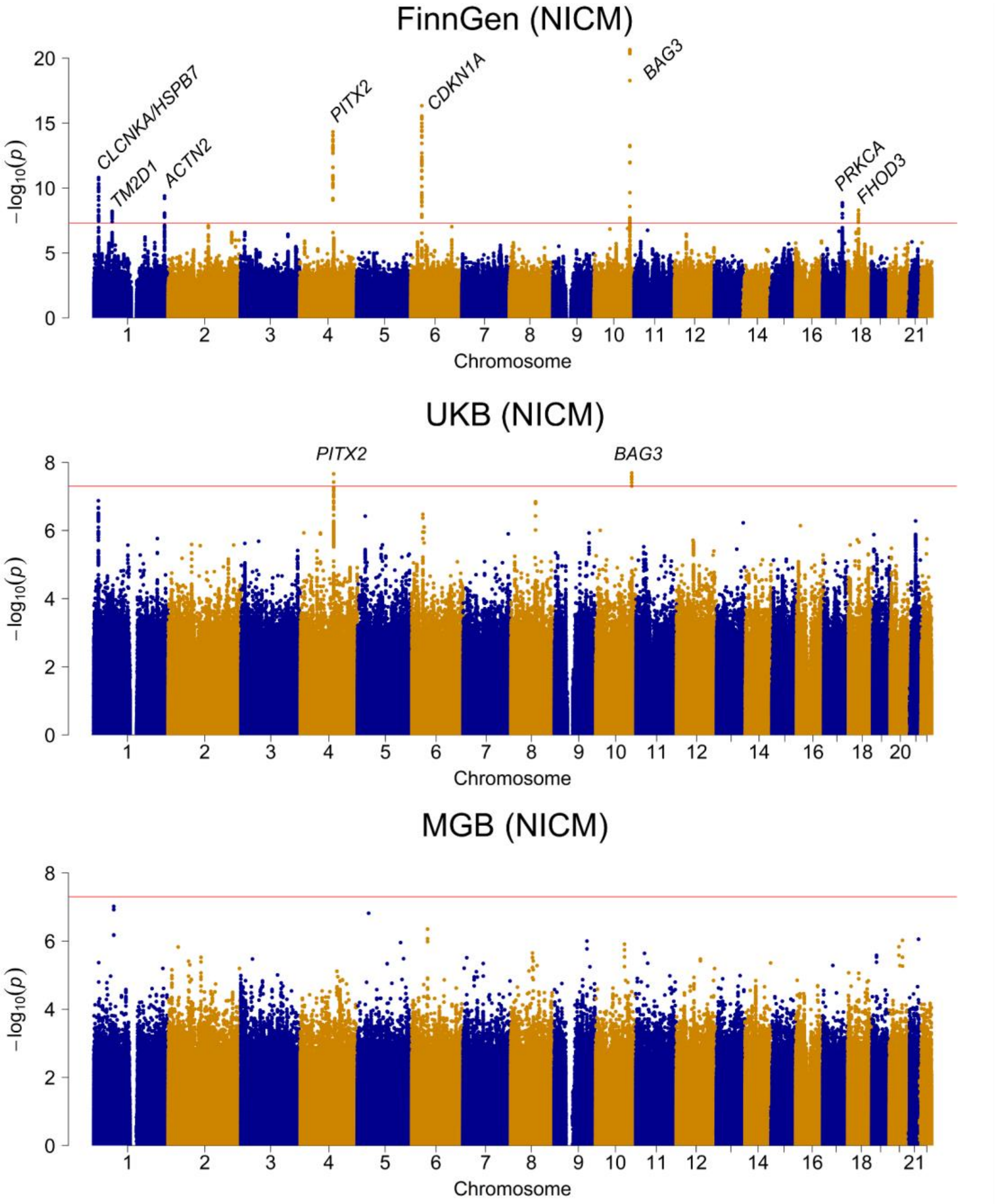

Supplementary Figure 3: Manhattan plots for biobank datasets for NICM

Each panel shows a Manhattan for GWAS of NICM in a dataset, where each dot represents a single tested variant, the x-axis shows genomic coordinates for those variants (chromosome, and position on chromosome), while the y-axis shows the -log10 of the *P*-value from GWAS. The red line indicates the conventional genome-wide significance level (alpha=5x10^{-8}). Loci reaching above the significance line are annotated with a gene name, where the annotated

gene is harmonized with the locus name from our main GWAS (ie, highest prioritized gene in locus from GWAS-DCM/MTAG-DCM) for easy comparisons; sometimes an additional gene is highlighted to serve easier comparison to previously-published GWAS; if locus was not identified in GWAS-DCM/MTAG-DCM, the closest protein-coding gene is used. *P*-values are derived from various logistic regression models; reported *P*-values are two-sided and unadjusted for multiple testing. Note: GWAS, genome-wide association study; NICM, nonischemic cardiomyopathy

Genetic correlation matrix

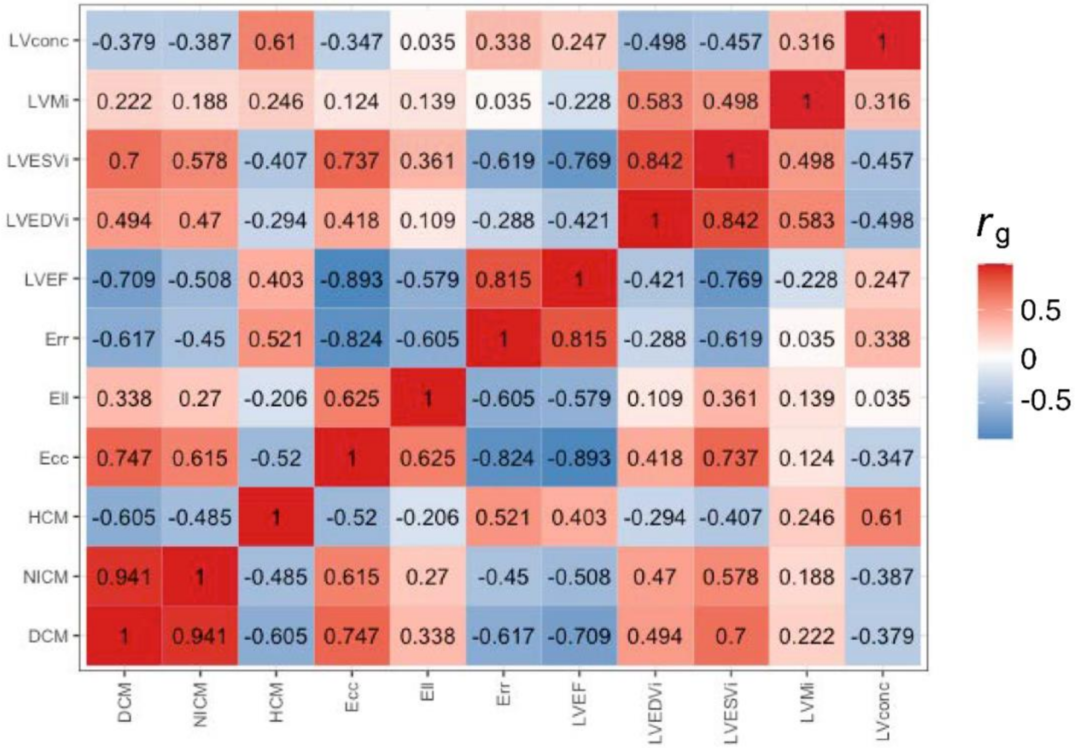

Genetic correlation matrix (absolute)

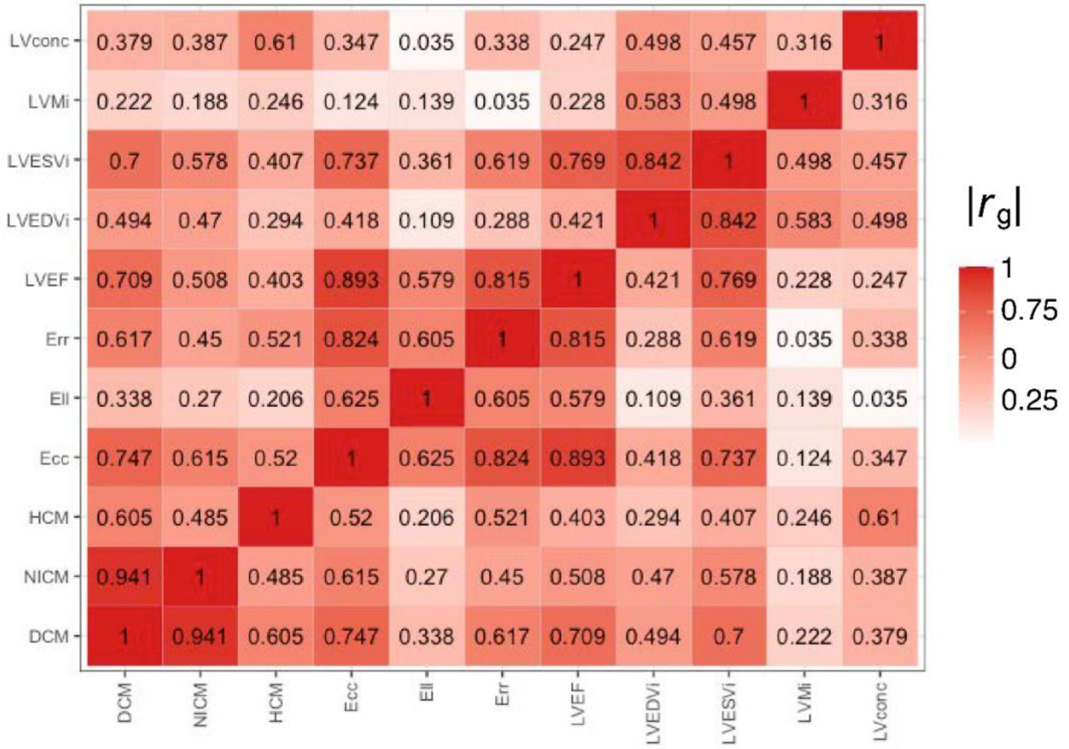

Supplementary Figure 4: Matrix of genetic correlations between DCM, other cardiomyopathic diseases, and left ventricular traits from cardiac MRI, estimated using MTAG.

These heatmaps represent correlation matrices, showing the genetic correlation between DCM (bottom left; based on GWAS-DCM meta-analysis), NICM (based on a biobank meta-analysis), HCM (based on a recent meta-analysis[52]), and 8 cardiac MRI traits (from a recent analysis[52]). In the top panel, the genetic correlations are shown with red indicating a positive value and blue indicating a negative value, while in the bottom panel the values are transformed to the absolute value for visual purposes. In both, the darker the color, the further the value is from 0. Note: MRI, magnetic resonance imaging; GWAS, genome-wide association study; DCM, dilated cardiomyopathy; NICM, nonischemic cardiomyopathy from biobank analysis; HCM, hypertrophic cardiomyopathy; Ecc, global circumferential strain; Ell, global longitudinal strain; Err, global radial strain; LVEF, left ventricular ejection fraction; LVEDVi, left ventricular end-diastolic volume indexed to body-mass-index; LVESVi, left ventricular end-systolic volume indexed to body-mass-index; LVMi, left ventricular mass indexed to body-mass-index; LVconc; left ventricular concentricity; rg, genetic correlation; abs, absolute.

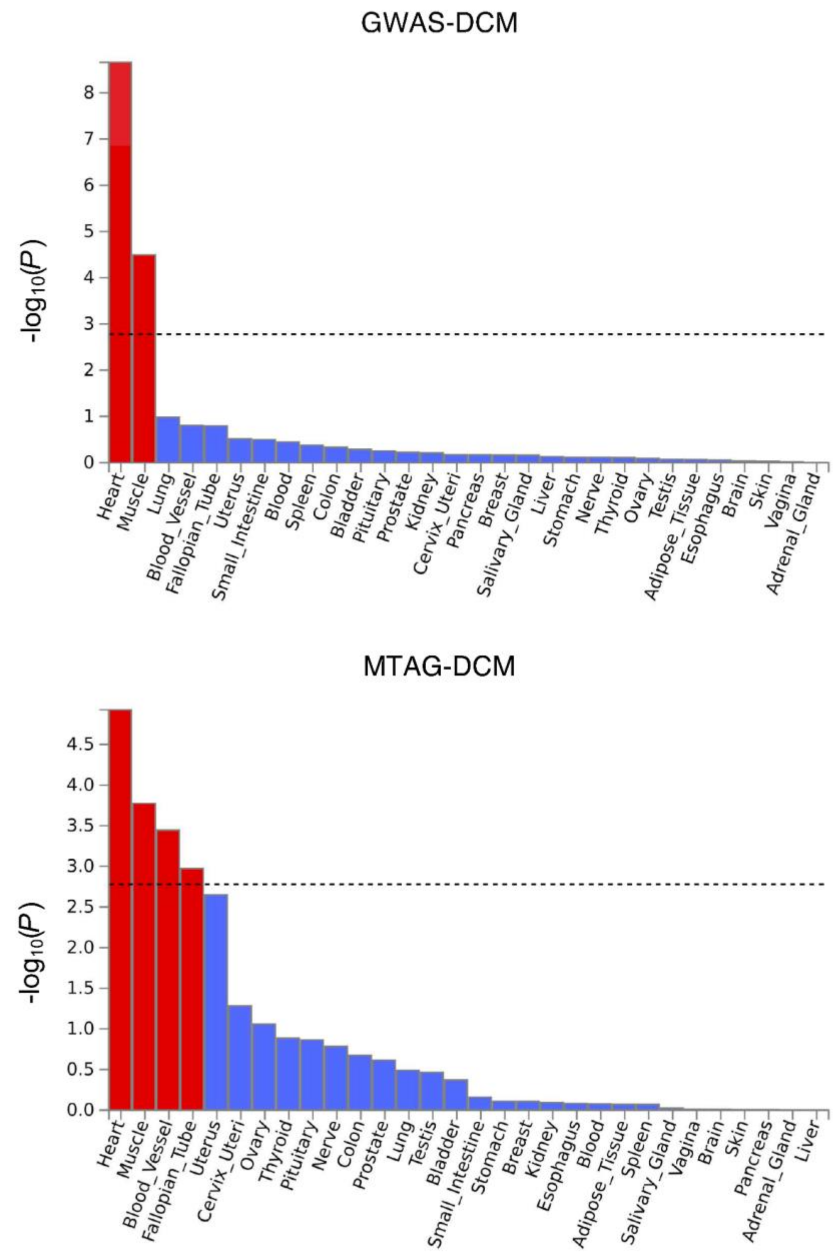

Supplementary Figure 5: Tissue enrichment of DCM heritability from bulk RNA sequencing data in GTEx v8.

In both figures, bar charts represent the -log10 of the *P*-value from the enrichment analysis, with the x-axis showing different tissues from GTEx. Tissues reaching the Bonferroni significance level are colored red. The dotted line represents the significance cutoff; tissues are ordered by their significance level. The top figure shows the results for GWAS-DCM, while the bottom shows results for MTAG-DCM. P-values are derived from a hypergeometric test; P-values can

be considered one-sided and unadjusted for multiple testing. Note: GWAS, genome-wide association study; MTAG, multi-trait analysis GWAS; DCM, dilated cardiomyopathy

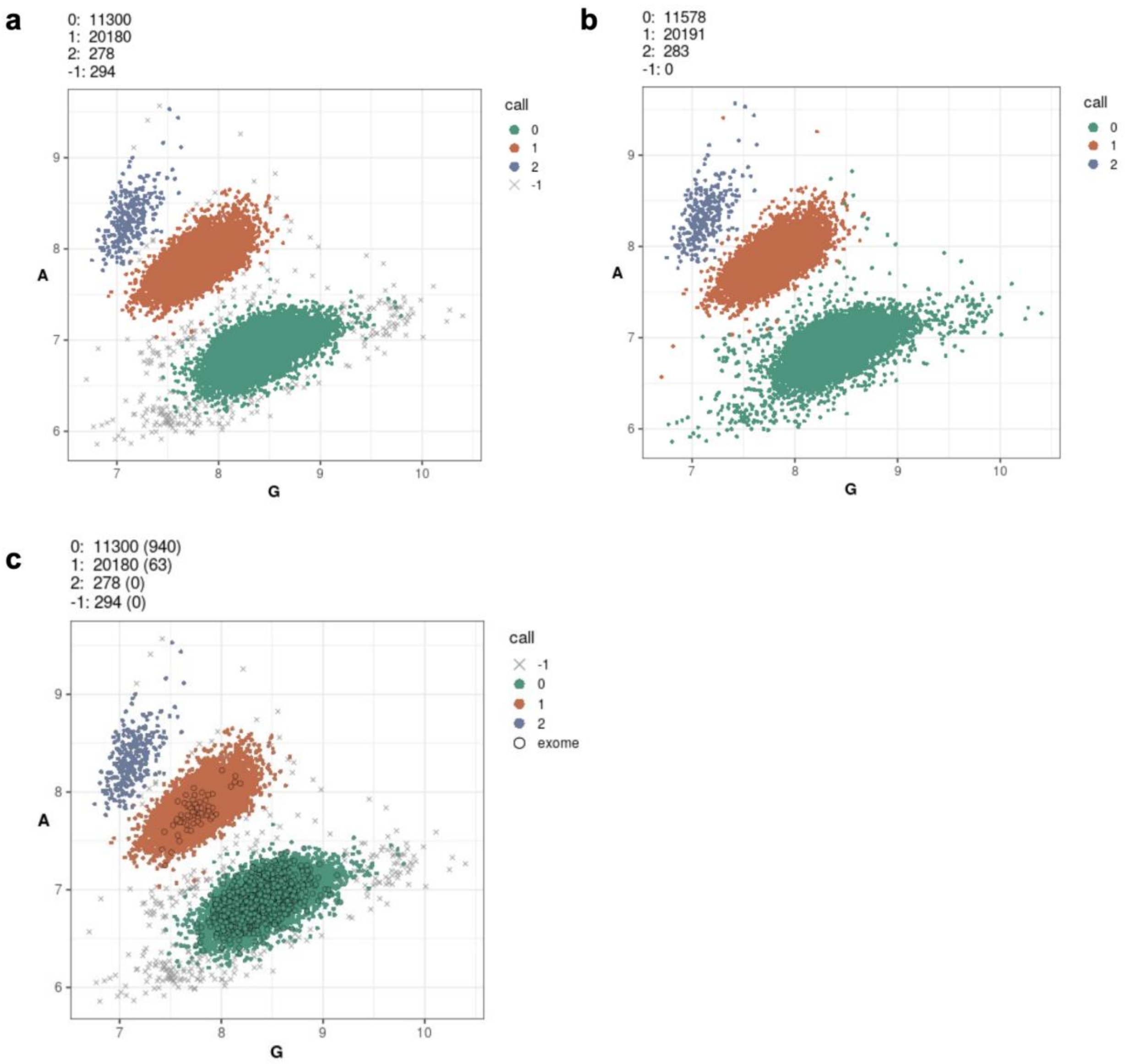

Supplementary Figure 6: Genotype cluster plots for the *TUBA8* variant 22:18609493:G:A in FinnGen

Part **a** shows signal intensities and assigned genotype clusters based on chip data for 22:18609493:G:A among 16,026 individuals from the FinnGen study. Part **b** shows imputed genotypes for the same individuals overlaid on the signal intensities from chip data. Part **c** shows exome sequencing calls in a subset of individuals demonstrating concordance with chip data. The position of the variant is listed in the GRCh37 build.

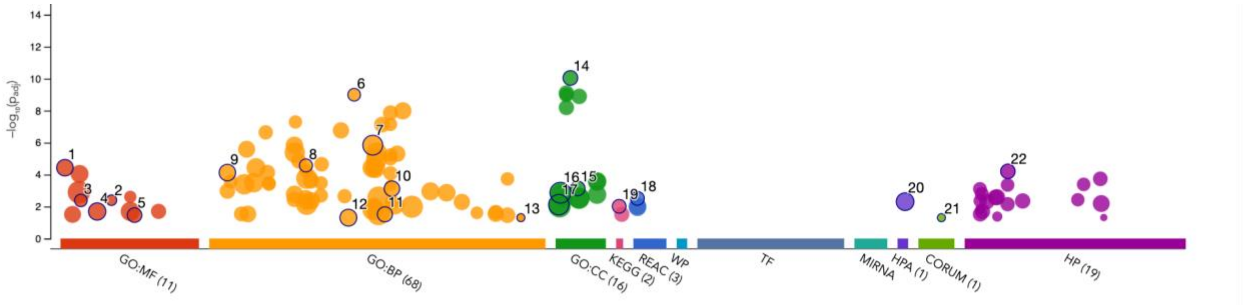

ID	Source	Term ID	Term Name	p_{adj} (query_...
1	GO:MF	GO:0003779	actin binding	3.712×10^{-5}
2	GO:MF	GO:0031432	titin binding	4.002×10^{-3}
3	GO:MF	GO:0008307	structural constituent of muscle	4.148×10^{-3}
4	GO:MF	GO:0016773	phosphotransferase activity, alcohol group as ...	2.032×10^{-2}
5	GO:MF	GO:0044325	transmembrane transporter binding	3.430×10^{-2}
6	GO:BP	GO:0045214	sarcomere organization	1.016×10^{-9}
7	GO:BP	GO:0048513	animal organ development	1.442×10^{-6}
8	GO:BP	GO:0032413	negative regulation of ion transmembrane tran...	2.684×10^{-5}
9	GO:BP	GO:0003012	muscle system process	7.487×10^{-5}
10	GO:BP	GO:0060047	heart contraction	7.630×10^{-4}
11	GO:BP	GO:0051495	positive regulation of cytoskeleton organization	3.079×10^{-2}
12	GO:BP	GO:0044057	regulation of system process	4.939×10^{-2}
13	GO:BP	GO:1905337	positive regulation of aggrephagy	4.956×10^{-2}
14	GO:CC	GO:0031674	I band	9.029×10^{-11}
15	GO:CC	GO:0042383	sarcolemma	7.354×10^{-4}
16	GO:CC	GO:0005829	cytosol	1.362×10^{-3}
17	GO:CC	GO:0005654	nucleoplasm	7.636×10^{-3}
18	REAC	REAC:R-HSA-3...	Cellular response to heat stress	3.081×10^{-3}
19	KEGG	KEGG:04012	ErbB signaling pathway	9.620×10^{-3}
20	HPA	HPA:0440343	skeletal muscle; myocytes[High]	4.952×10^{-3}
21	CORUM	CORUM:6052	BAG3-HSC70-HSPB8-CHIP complex	4.987×10^{-2}
22	HP	HP:0005162	Abnormal left ventricular function	6.239×10^{-5}

Supplementary Figure 7: Results from pathway enrichment analysis using g:Profiler.

The top of the figure represents a dot plot with on the x-axis different gene-set categories (ie, sources) implemented in g:Profiler, and on the y-axis multiple-testing adjusted one-sided *P*-values (on the -log10 scale). Each dot represents a different gene-set from the respective categories, which are restricted only to signals reaching multiple testing-adjusted one-sided *P*<0.05. *P*-values are derived from one-sided Fisher exact tests. Select gene-sets are highlighted with numbers. The bottom of the figure represents a table with results for the highlighted gene-sets. Note: adj, adjusted; GO:MF, gene ontology molecular function; GO:BP, gene ontology biological process; GO:CC, gene ontology cellular component; KEGG, Kyoto Encyclopedia of Genes and Genomes; REAC, reactome; WP, WikiPathways; TF, transcription factor targets database; MIRNA, the experimentally validated microRNA-target interactions database; HPA, Human Phenotype Ontology; CORUM, comprehensive resource of mammalian protein complexes; HP, human phenome.

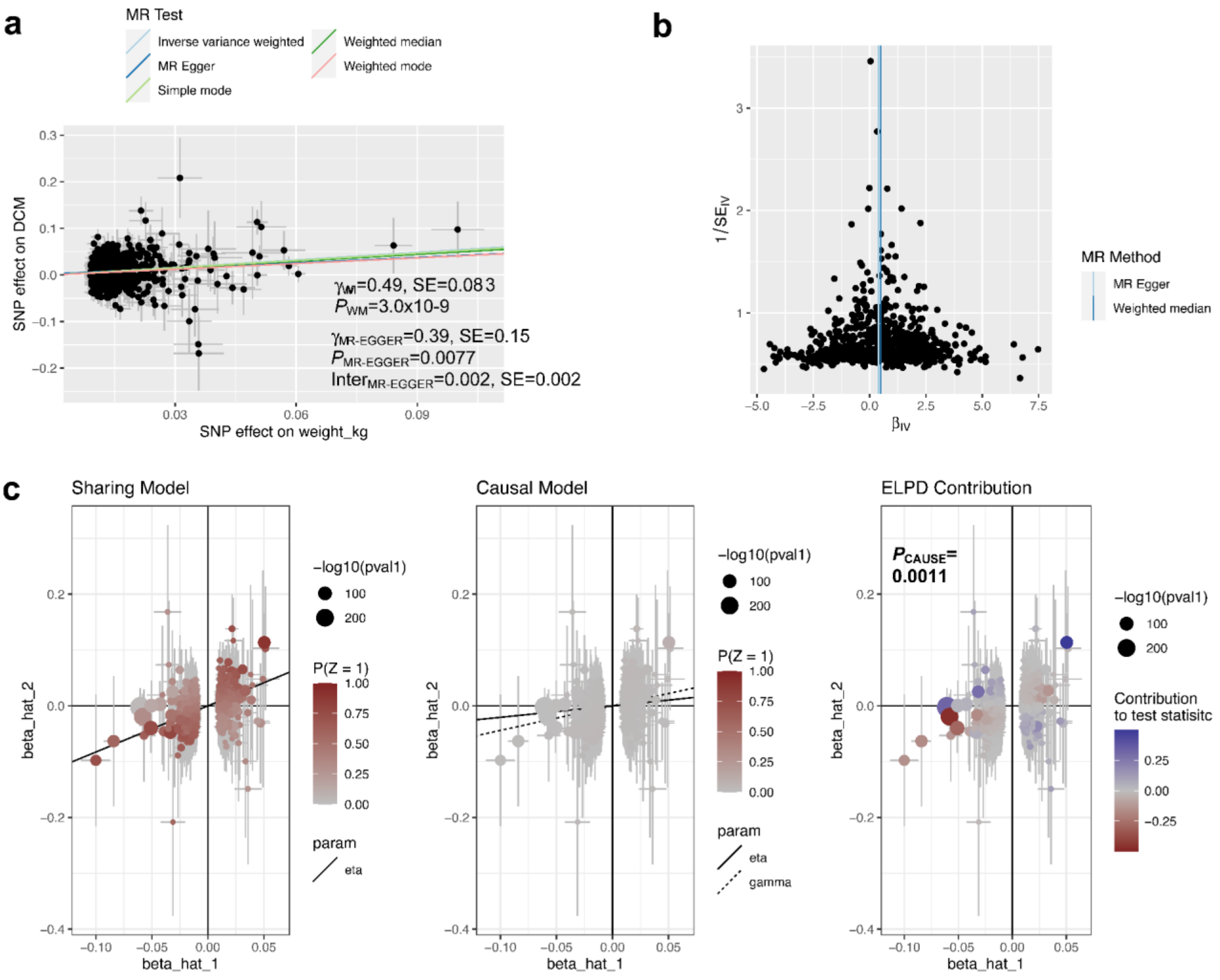

Supplementary Figure 8: Plots for Mendelian randomization analyses for weight->DCM.

Part **a** shows a scatter plot from a Mendelian randomization analysis for the association of body weight on risk of DCM (from GWAS-DCM). The x-axis shows the beta coefficients for the genetic instruments for body weight (from published GWAS[50]), while the y-axis shows the respective beta coefficients in DCM (from GWAS-DCM). Data are presented as estimated beta coefficients +/- standard errors. The Mendelian randomization estimates for the causal effect are added for various common methods. The regression parameters are added for the weighted median method, and for the MR-Egger method. $N_{instruments}$=733. Part **b** shows a funnel plot for the genetic instruments taking into account the estimated causal effects and their errors. The y-axis shows the estimated causal effect for each instrument, while the x-axis shows the inverse of the error of the estimate. The estimates follow a funnel shape, where the largest spread is found for estimates with the largest error, as expected. Part **c** shows results from the Mendelian

randomization analysis using CAUSE. In each plot, the x-axis shows the beta coefficients for the genetic instruments for body weight (from published GWAS[50]), while the y-axis shows the respective beta coefficients in DCM (from GWAS-DCM); error bars represent 95% confidence intervals. In each plot the size of the dots represents the instrument strength (based on -log10 of the exposure trait *P*-value). The left plot shows results from the 'sharing' model where only a pleiotropic pathway is modeled; here the black line represents the effect of the pleiotropic pathway. The middle plot represents results from the 'causal' model, where both a pleiotropic (black line) and causal pathway (dotted line) are modeled. The right plot shows the contribution of different variants to the ELPD test statistic of CAUSE, with brown indicating more favorable for a causal model and blue indicating less favorable for a causal model. The one-sided *P*-value from a Z-test comparing the causal model to the sharing model is added to the plot. $N_{instruments}$=2286. Note: MR, mendelian randomization; WM, weighted median; γ (gamma), estimated causal effect (slope); Inter, intercept from MR-Egger regression; SE, standard error; IV, instrumental variable; eta, estimated effect of a pleiotropic pathway. ELPD, expected log pointwise posterior density.

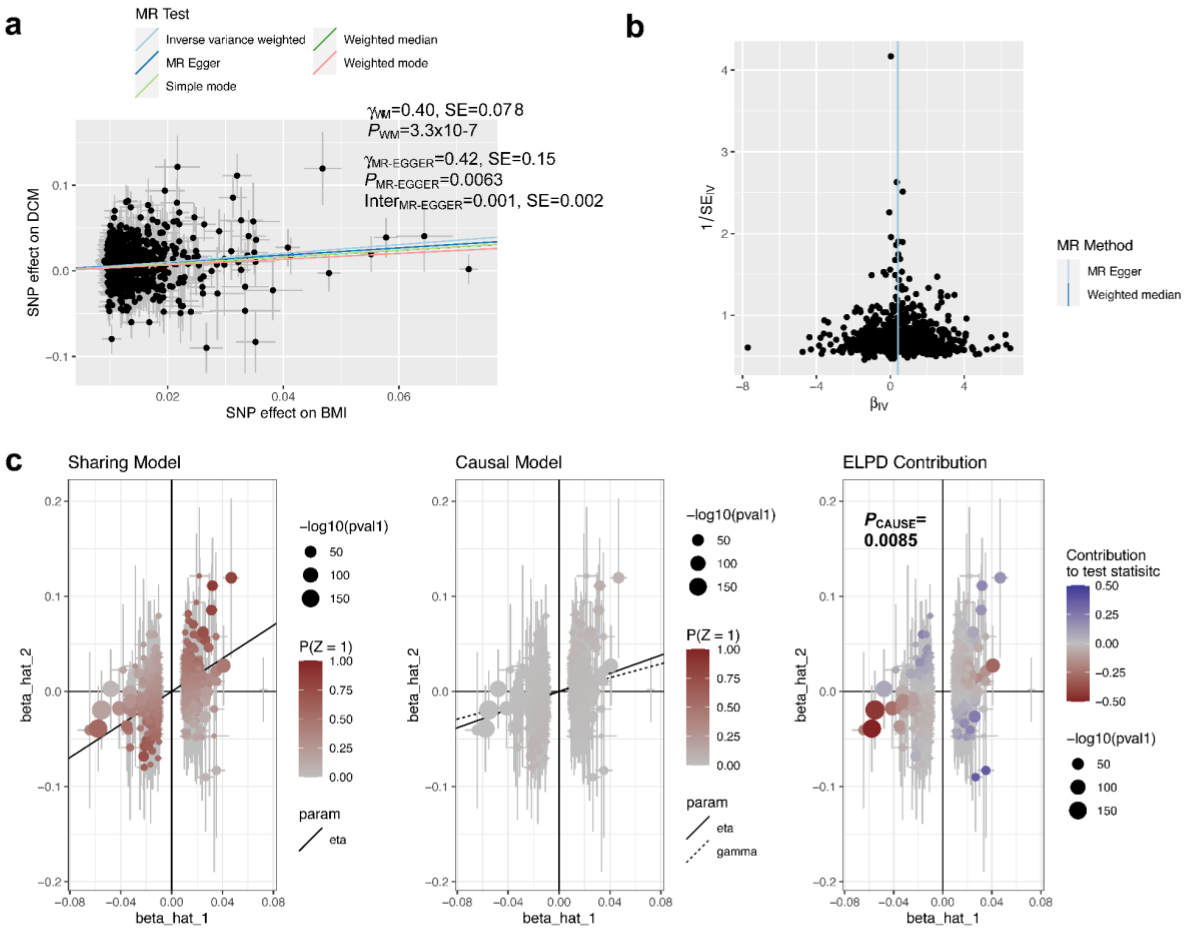

Supplementary Figure 9: Plots for Mendelian randomization analyses for BMI->DCM.

Part **a** shows a scatter plot from a Mendelian randomization analysis for the association of BMI on risk of DCM (from GWAS-DCM). The x-axis shows the beta coefficients for the genetic instruments for BMI (from published GWAS[53]), while the y-axis shows the respective beta coefficients in DCM (from GWAS-DCM). Data are presented as estimated beta coefficients +/- standard errors. The Mendelian randomization estimates for the causal effect are added for various common methods. The regression parameters are added for the weighted median method, and for the MR-Egger method. $N_{instruments}$=729. Part **b** shows a funnel plot for the genetic instruments taking into account the estimated causal effects and their errors.The y-axis shows the estimated causal effect for each instrument, while the x-axis shows the inverse of the error of the estimate. The estimates follow a funnel shape, where the largest spread is found for estimates with the largest error, as expected. Part **c** shows results from the Mendelian randomization analysis using CAUSE. In each plot, the x-axis shows the beta coefficients for the genetic instruments for BMI, while the y-axis shows the respective beta coefficients in DCM (from GWAS-DCM); error bars represent 95% confidence intervals. In each plot the size of the

dots represents the instrument strength (based on -log10 of the exposure trait *P*-value). The left plot shows results from the 'sharing' model where only a pleiotropic pathway is modeled; here the black line represents the effect of the pleiotropic pathway. The middle plot represents results from the 'causal' model, where both a pleiotropic (black line) and causal pathway (dotted line) are modeled. The right plot shows the contribution of different variants to the ELPD test statistic of CAUSE, with brown indicating more favorable for a causal model and blue indicating less favorable for a causal model. The one-sided *P*-value from a Z-test comparing the causal model to the sharing model is added to the plot. $N_{instruments}$=2223. Note: BMI, body-mass-index; MR, mendelian randomization; WM, weighted median; γ (gamma), estimated causal effect (slope); Inter, intercept from MR-Egger regression; SE, standard error; IV, instrumental variable; eta, estimated effect of a pleiotropic pathway. ELPD, expected log pointwise posterior density.

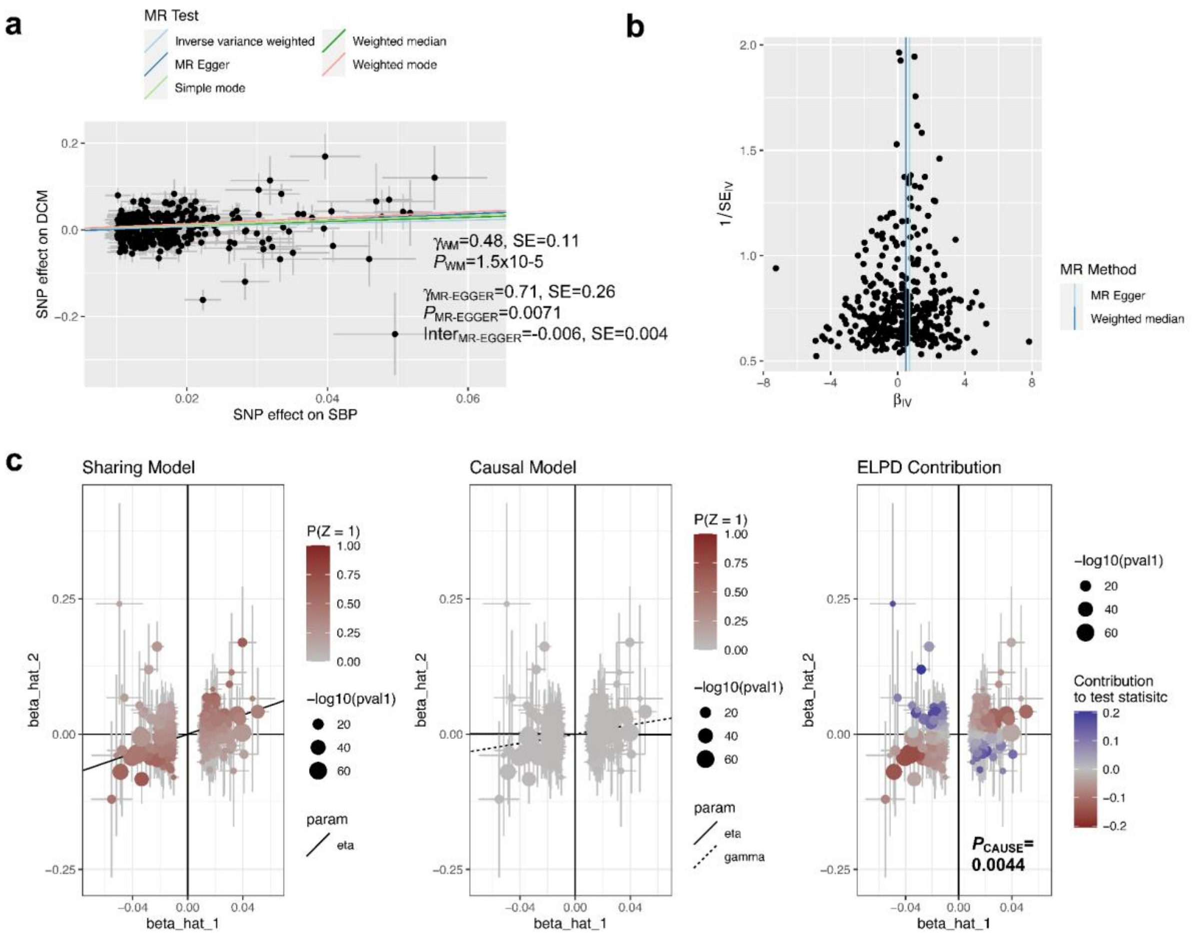

Supplementary Figure 10: Plots for Mendelian randomization analyses for systolic blood pressure->DCM.

Part **a** shows a scatter plot from a Mendelian randomization analysis for the association of systolic blood pressure on risk of DCM (from GWAS-DCM). The x-axis shows the beta coefficients for the genetic instruments for systolic blood pressure (from published GWAS[50]), while the y-axis shows the respective beta coefficients in DCM (from GWAS-DCM). Data are presented as estimated beta coefficients +/- standard errors. Mendelian randomization estimates for the causal effect are added for various common methods. The regression parameters are added for the weighted median method, and for the MR-Egger method. $N_{instruments}$=376. Part **b** shows a funnel plot for the genetic instruments taking into account the estimated causal effects and their errors. The y-axis shows the estimated causal effect for each instrument, while the x-axis shows the inverse of the error of the estimate. The estimates follow a funnel shape, where the largest spread is found for estimates with the largest error, as expected. Part **c** shows results from the Mendelian randomization analysis using CAUSE. In each plot, the x-axis shows the beta coefficients for the genetic instruments for systolic blood pressure (from published GWAS[50]), while the y-axis shows the respective beta coefficients in

DCM (from GWAS-DCM); error bars represent 95% confidence intervals. In each plot the size of the dots represents the instrument strength (based on -log10 of the exposure trait *P*-value). The left plot shows results from the 'sharing' model where only a pleiotropic pathway is modeled; here the black line represents the effect of the pleiotropic pathway. The middle plot represents results from the 'causal' model, where both a pleiotropic (black line) and causal pathway (dotted line) are modeled. The right plot shows the contribution of different variants to the ELPD test statistic of CAUSE, with brown indicating more favorable for a causal model and blue indicating less favorable for a causal model. The one-sided *P*-value from a Z-test comparing the causal model to the sharing model is added to the plot. $N_{instruments}$=1846. Note: SBP, systolic blood pressure; MR, mendelian randomization; WM, weighted median; γ (gamma), estimated causal effect (slope); Inter, intercept from MR-Egger regression; SE, standard error; IV, instrumental variable; eta, estimated effect of a pleiotropic pathway. ELPD, expected log pointwise posterior density.

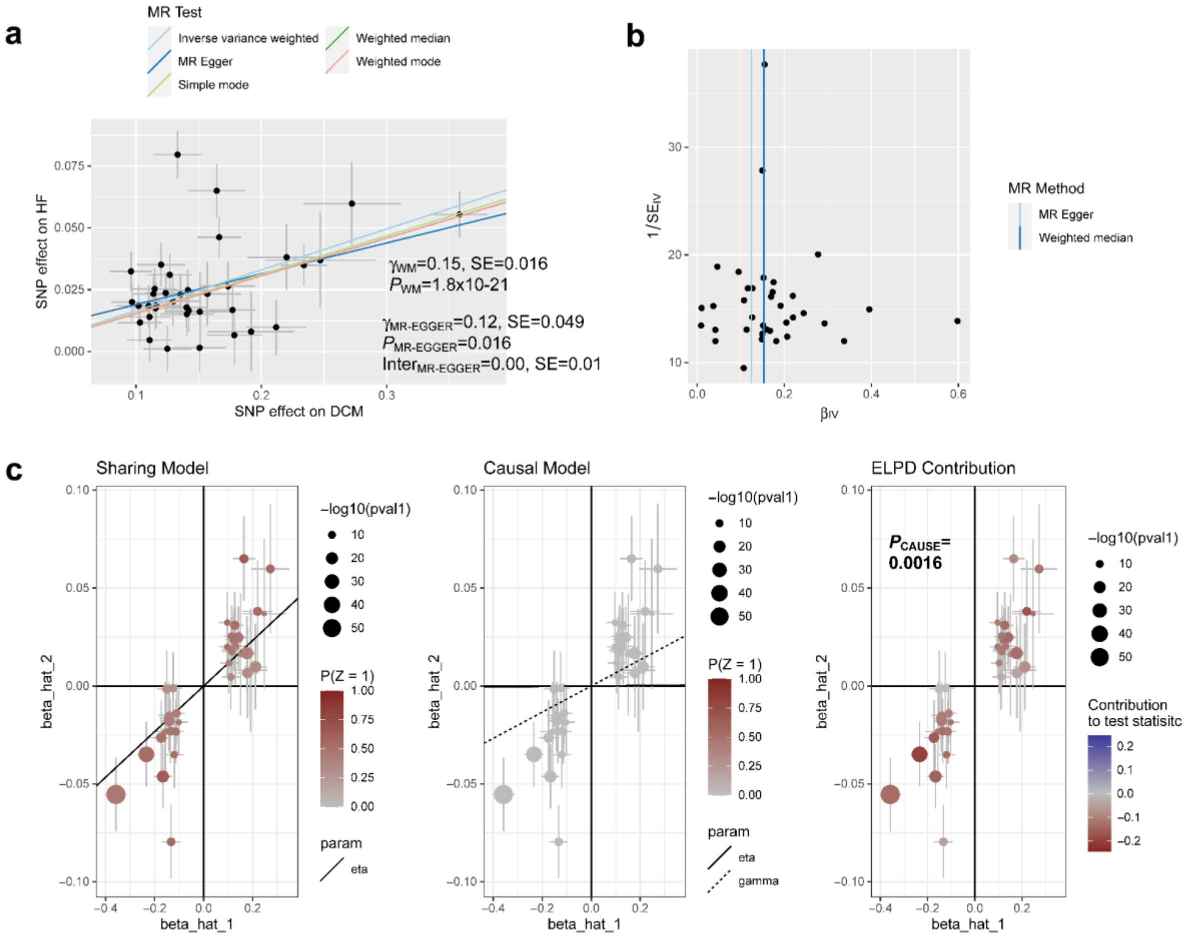

Supplementary Figure 11: Plots for Mendelian randomization analyses for DCM->heart failure.

Part **a** shows a scatter plot from a Mendelian randomization analysis for the association of DCM (from GWAS-DCM) on risk of HF (from published GWAS[54]). The x-axis shows the beta coefficients for the genetic instruments for DCM, while the y-axis shows the respective beta coefficients in HF. Data are presented as estimated beta coefficients +/- standard errors. The Mendelian randomization estimates for the causal effect are added for various common methods. The regression parameters are added for the weighted median method, and for the MR-Egger method. $N_{instruments}$=37. Part **b** shows a funnel plot for the genetic instruments taking into account the estimated causal effects and their errors. The y-axis shows the estimated causal effect for each instrument, while the x-axis shows the inverse of the error of the estimate. Part **c** shows results from the Mendelian randomization analysis using CAUSE. In each plot, the x-axis shows the beta coefficients for the genetic instruments for DCM (from GWAS-DCM), while the y-axis shows the respective beta coefficients in HF (from published GWAS[52]); error bars represent 95% confidence intervals. In each plot the size of the dots represents the instrument strength (based on -log10 of the exposure trait *P*-value). The left plot shows results

from the ‘sharing’ model where only a pleiotropic pathway is modeled; here the black line represents the effect of the pleiotropic pathway. The middle plot represents results from the ‘causal’ model, where both a pleiotropic (black line) and causal pathway (dotted line) are modeled. The right plot shows the contribution of different variants to the ELPD test statistic of CAUSE, with brown indicating more favorable for a causal model and blue indicating less favorable for a causal model. The one-sided *P*-value from a Z-test comparing the causal model to the sharing model is added to the plot. $N_{instruments}$=1050. Note: HF, heart failure; MR, mendelian randomization; WM, weighted median; γ (gamma), estimated causal effect (slope); Inter, intercept from MR-Egger regression; SE, standard error; IV, instrumental variable; eta, estimated effect of a pleiotropic pathway. ELPD, expected log pointwise posterior density.

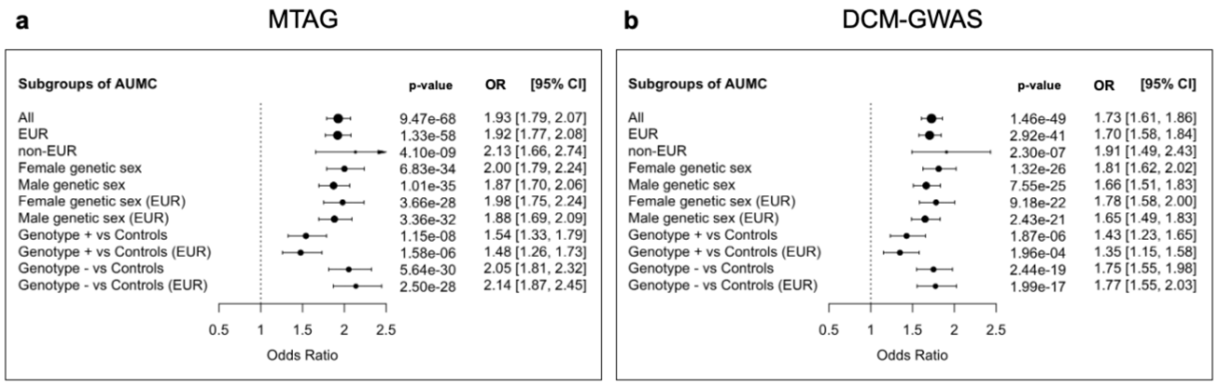

Supplementary Figure 12: Associations between DCM polygenic risk score and DCM across various subsets of the Amsterdam UMC dataset.

This forest plot shows association results for prediction of DCM using PRS, within the Amsterdam UMC dataset. Data are presented as odds ratios with 95% confidence intervals. PRS were constructed from MTAG-DCM and GWAS-DCM summary statistics after omitting the Amsterdam cohort from our meta-analyses. The figure shows results for various subsets of the Amsterdam cohort, including All samples, European ancestry only, non-European ancestry, males, females, genotype-positive samples, and genotype-negative samples. Data are based on – at most – 8185 participants of which 978 DCM cases; please see **Supplementary Table 32** for exact N numbers of each group. Note: GWAS, genome-wide association study; DCM, dilated cardiomyopathy; MTAG, multi-trait analysis GWAS; OR, odds ratio; 95%CI, 95% confidence interval.

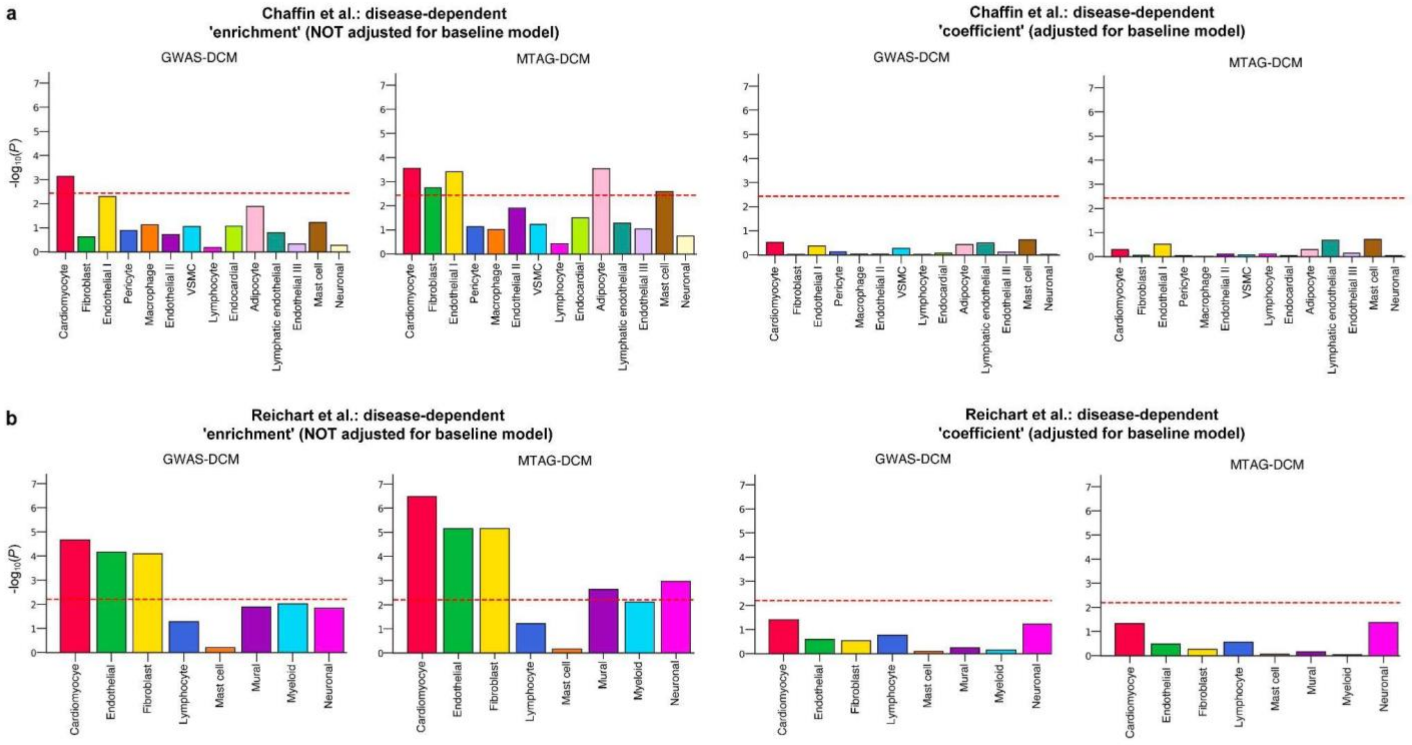

Supplementary Figure 13: Results from cell type enrichment analysis using disease-dependent gene programs.

This figure shows bar plots with on the y-axis the -log10 *P*-value of enrichment statistics, and on the x-axis different cell types identified from snRNAseq data. Part **a** shows results for the Chaffin et al. dataset, while part **b** shows results for the Reichart et al. dataset. In each panel, the left plot shows enrichment for GWAS-DCM heritability, while the right plot shows enrichment for MTAG-DCM heritability. In these analyses, cell type gene programs were based on genes with differential expression between DCM LVs and non-failing LVs. This contrasts with our main enrichment analysis, which was based on genes with cell type-specific expression (shown in **Extended Data Figure 5**). In this figure, we additionally show enrichment *P*-values based on two different enrichment parameters: The left part of the figure shows results for the 'enrichment' statistic (*Ec*), while the right part of the figure shows results for the *'Tau_C'* parameter. Throughout our work, we determine significant cell types based only on the *Tau_C* parameter (see Finucane et al. for more details on the parameters[42,46]). *P*-values are one-sided and unadjusted for multiple testing.

Supplementary References

1. Garnier, S. *et al.* Genome-wide association analysis in dilated cardiomyopathy reveals two new players in systolic heart failure on chromosomes 3p25.1 and 22q11.23. *Eur. Heart J.* **42**, 2000–2011 (2021).
2. Meder, B. *et al.* A genome-wide association study identifies 6p21 as novel risk locus for dilated cardiomyopathy. *Eur. Heart J.* **35**, 1069–1077 (2014).
3. 1000 Genomes Project Consortium *et al.* A global reference for human genetic variation. *Nature* **526**, 68–74 (2015).
4. Gerber, T. C., Kantor, B. & Williamson, E. E. *Computed Tomography of the Cardiovascular System*. (CRC Press, 2007).
5. Purcell, S. *et al.* PLINK: a tool set for whole-genome association and population-based linkage analyses. *Am. J. Hum. Genet.* **81**, 559–575 (2007).
6. Delaneau, O., Howie, B., Cox, A. J., Zagury, J.-F. & Marchini, J. Haplotype estimation using sequencing reads. *Am. J. Hum. Genet.* **93**, 687–696 (2013).
7. Howie, B. N., Donnelly, P. & Marchini, J. A flexible and accurate genotype imputation method for the next generation of genome-wide association studies. *PLoS Genet.* **5**, e1000529 (2009).
8. Marchini, J. & Howie, B. Comparing algorithms for genotype imputation. *American journal of human genetics* vol. 83 535–9; author reply 539–40 (2008).
9. Pinto, Y. M. *et al.* Proposal for a revised definition of dilated cardiomyopathy, hypokinetic non-dilated cardiomyopathy, and its implications for clinical practice: a position statement of the ESC working group on myocardial and pericardial diseases. *Eur. Heart J.* **37**, 1850–1858 (2016).
10. Ligthart, L. *et al.* The Netherlands Twin Register: Longitudinal Research Based on Twin

and Twin-Family Designs. *Twin Res. Hum. Genet.* **22**, 623–636 (2019).

11. Chang, C. C. *et al.* Second-generation PLINK: rising to the challenge of larger and richer datasets. *Gigascience* **4**, 7 (2015).
12. Gogarten, S. M. *et al.* Genetic association testing using the GENESIS R/Bioconductor package. *Bioinformatics* **35**, 5346–5348 (2019).
13. Manichaikul, A. *et al.* Robust relationship inference in genome-wide association studies. *Bioinformatics* **26**, 2867–2873 (2010).
14. Mbatchou, J. *et al.* Computationally efficient whole-genome regression for quantitative and binary traits. *Nat. Genet.* **53**, 1097–1103 (2021).
15. Jordan, E. *et al.* Evidence-Based Assessment of Genes in Dilated Cardiomyopathy. *Circulation* **144**, 7–19 (2021).
16. Kurki, M. I. *et al.* FinnGen provides genetic insights from a well-phenotyped isolated population. *Nature* **613**, 508–518 (2023).
17. Aragam, K. G. *et al.* Phenotypic Refinement of Heart Failure in a National Biobank Facilitates Genetic Discovery. *Circulation* **139**, 489–501 (2019).
18. Bycroft, C. *et al.* The UK Biobank resource with deep phenotyping and genomic data. *Nature* **562**, 203–209 (2018).
19. Jurgens, S. J. *et al.* Analysis of rare genetic variation underlying cardiometabolic diseases and traits among 200,000 individuals in the UK Biobank. *Nat. Genet.* **54**, 240–250 (2022).
20. Karlson, E. W., Boutin, N. T., Hoffnagle, A. G. & Allen, N. L. Building the Partners HealthCare Biobank at Partners Personalized Medicine: Informed Consent, Return of Research Results, Recruitment Lessons and Operational Considerations. *J Pers Med* **6**, (2016).
21. Mccormick, J. B. & Pathak, J. *Genomic Data Sharing: Case Studies, Challenges, and Opportunities for Precision Medicine*. (Academic Press, 2022).
22. Conomos, M. P., Reiner, A. P., Weir, B. S. & Thornton, T. A. Model-free Estimation of

Recent Genetic Relatedness. *Am. J. Hum. Genet.* **98**, 127–148 (2016).

23. Conomos, M. P., Miller, M. B. & Thornton, T. A. Robust inference of population structure for ancestry prediction and correction of stratification in the presence of relatedness. *Genet. Epidemiol.* **39**, 276–293 (2015).
24. Taliun, D. *et al.* Sequencing of 53,831 diverse genomes from the NHLBI TOPMed Program. *Nature* **590**, 290–299 (2021).
25. Venner, E. *et al.* Whole-genome sequencing as an investigational device for return of hereditary disease risk and pharmacogenomic results as part of the All of Us Research Program. *Genome Med.* **14**, 34 (2022).
26. Karczewski, K. J. *et al.* The mutational constraint spectrum quantified from variation in 141,456 humans. *Nature* **581**, 434–443 (2020).
27. Biddinger, K. J. *et al.* Rare and Common Genetic Variation Underlying the Risk of Hypertrophic Cardiomyopathy in a National Biobank. *JAMA Cardiol* **7**, 715–722 (2022).
28. Halford, J. L. *et al.* Endophenotype effect sizes support variant pathogenicity in monogenic disease susceptibility genes. *Nat. Commun.* **13**, 5106 (2022).
29. Bulik-Sullivan, B. K. *et al.* LD Score regression distinguishes confounding from polygenicity in genome-wide association studies. *Nat. Genet.* **47**, 291–295 (2015).
30. Bulik-Sullivan, B. *et al.* An atlas of genetic correlations across human diseases and traits. *Nat. Genet.* **47**, 1236–1241 (2015).
31. Pirruccello, J. P. *et al.* Analysis of cardiac magnetic resonance imaging in 36,000 individuals yields genetic insights into dilated cardiomyopathy. *Nat. Commun.* **11**, 2254 (2020).
32. Levin, M. G. *et al.* Genome-wide association and multi-trait analyses characterize the common genetic architecture of heart failure. *Nat. Commun.* **13**, 6914 (2022).
33. Zheng, S. L. *et al.* Genome-wide association analysis reveals insights into the molecular etiology underlying dilated cardiomyopathy. *medRxiv* (2023)

doi:10.1101/2023.09.28.23295408.

34. Gaziano, J. M. *et al.* Million Veteran Program: A mega-biobank to study genetic influences on health and disease. *J. Clin. Epidemiol.* **70**, 214–223 (2016).
35. Klarin, D. *et al.* Genetics of blood lipids among ~300,000 multi-ethnic participants of the Million Veteran Program. *Nat. Genet.* **50**, 1514–1523 (2018).
36. Hunter-Zinck, H. *et al.* Genotyping Array Design and Data Quality Control in the Million Veteran Program. *Am. J. Hum. Genet.* **106**, 535–548 (2020).
37. Fang, H. *et al.* Harmonizing Genetic Ancestry and Self-identified Race/Ethnicity in Genome-wide Association Studies. *Am. J. Hum. Genet.* **105**, 763–772 (2019).
38. Alexander, D. H., Novembre, J. & Lange, K. Fast model-based estimation of ancestry in unrelated individuals. *Genome Res.* **19**, 1655–1664 (2009).
39. Delaneau, O., Zagury, J.-F., Robinson, M. R., Marchini, J. L. & Dermitzakis, E. T. Accurate, scalable and integrative haplotype estimation. *Nat. Commun.* **10**, 5436 (2019).
40. Howie, B., Fuchsberger, C., Stephens, M., Marchini, J. & Abecasis, G. R. Fast and accurate genotype imputation in genome-wide association studies through pre-phasing. *Nat. Genet.* **44**, 955–959 (2012).
41. Chaffin, M. *et al.* Single-nucleus profiling of human dilated and hypertrophic cardiomyopathy. *Nature* **608**, 174–180 (2022).
42. Finucane, H. K. *et al.* Partitioning heritability by functional annotation using genome-wide association summary statistics. *Nat. Genet.* **47**, 1228–1235 (2015).
43. Robinson, M. D., McCarthy, D. J. & Smyth, G. K. edgeR: a Bioconductor package for differential expression analysis of digital gene expression data. *Bioinformatics* **26**, 139–140 (2010).
44. Love, M. I., Huber, W. & Anders, S. Moderated estimation of fold change and dispersion for RNA-seq data with DESeq2. *Genome Biol.* **15**, 550 (2014).
45. Ritchie, M. E. *et al.* limma powers differential expression analyses for RNA-sequencing and

microarray studies. *Nucleic Acids Res.* **43**, e47 (2015).

46. Finucane, H. K. *et al.* Heritability enrichment of specifically expressed genes identifies disease-relevant tissues and cell types. *Nat. Genet.* **50**, 621–629 (2018).
47. Reichart, D. *et al.* Pathogenic variants damage cell composition and single cell transcription in cardiomyopathies. *Science* **377**, eabo1984 (2022).
48. Jagadeesh, K. A. *et al.* Identifying disease-critical cell types and cellular processes by integrating single-cell RNA-sequencing and human genetics. *Nat. Genet.* **54**, 1479–1492 (2022).
49. Koenig, A. L. *et al.* Single-cell transcriptomics reveals cell-type-specific diversification in human heart failure. *Nat Cardiovasc Res* **1**, 263–280 (2022).
50. Jurgens, S. J. *et al.* Adjusting for common variant polygenic scores improves yield in rare variant association analyses. *Nat. Genet.* **55**, 544–548 (2023).
51. Ge, T., Chen, C.-Y., Ni, Y., Feng, Y.-C. A. & Smoller, J. W. Polygenic prediction via Bayesian regression and continuous shrinkage priors. *Nat. Commun.* **10**, 1776 (2019).
52. Tadros, R. *et al.* Large scale genome-wide association analyses identify novel genetic loci and mechanisms in hypertrophic cardiomyopathy. *medRxiv* (2023) doi:10.1101/2023.01.28.23285147.
53. Pulit, S. L. *et al.* Meta-analysis of genome-wide association studies for body fat distribution in 694 649 individuals of European ancestry. *Hum. Mol. Genet.* **28**, 166–174 (2019).
54. Shah, S. *et al.* Genome-wide association and Mendelian randomisation analysis provide insights into the pathogenesis of heart failure. *Nat. Commun.* **11**, 163 (2020).
