# Supplementary material for: Genome-wide association study reveals mechanisms underlying dilated cardiomyopathy and myocardial resilience
Source: Nat Genet. 2024 Nov 21;56(12):2636–45. doi: 10.1038/s41588-024-01975-5 (PMC11631763; doi:10.1038/s41588-024-01975-5)
Supplement: Supplementary file 2 — Reporting Summary [file 41588_2024_1975_MOESM2_ESM.pdf]

nature portfolio

Corresponding author(s): Connie R. Bezzina

Last updated by author(s): 8/26/2024

Reporting Summary

Nature Portfolio wishes to improve the reproducibility of the work that we publish. This form provides structure for consistency and transparency in reporting. For further information on Nature Portfolio policies, see our Editorial Policies and the Editorial Policy Checklist.

Statistics

For all statistical analyses, confirm that the following items are present in the figure legend, table legend, main text, or Methods section.

n/a	Confirmed	
☐	☒	The exact sample size (*n*) for each experimental group/condition, given as a discrete number and unit of measurement
☐	☒	A statement on whether measurements were taken from distinct samples or whether the same sample was measured repeatedly
☐	☒	The statistical test(s) used AND whether they are one- or two-sided<br>*Only common tests should be described solely by name; describe more complex techniques in the Methods section.*
☐	☒	A description of all covariates tested
☐	☒	A description of any assumptions or corrections, such as tests of normality and adjustment for multiple comparisons
☐	☒	A full description of the statistical parameters including central tendency (e.g. means) or other basic estimates (e.g. regression coefficient) AND variation (e.g. standard deviation) or associated estimates of uncertainty (e.g. confidence intervals)
☐	☒	For null hypothesis testing, the test statistic (e.g. *F*, *t*, *r*) with confidence intervals, effect sizes, degrees of freedom and *P* value noted<br>*Give P values as exact values whenever suitable.*
☒	☐	For Bayesian analysis, information on the choice of priors and Markov chain Monte Carlo settings
☒	☐	For hierarchical and complex designs, identification of the appropriate level for tests and full reporting of outcomes
☐	☒	Estimates of effect sizes (e.g. Cohen's *d*, Pearson's *r*), indicating how they were calculated

Our web collection on statistics for biologists contains articles on many of the points above.

Software and code

Policy information about availability of computer code

Data collection	For the Meder et al. and Garnier et al. clinical cohorts, data collection has been described previously, and no new software was used for data collection specific to the present study.<br><br>For the Amsterdam dataset, DCM cases were collected by clinicians and researchers using standard in-house electronic health record environments (ie, EPIC). Cases and controls were genotyped on the Illumina Global Screening Array; standard pre-processing and variant calling were applied by third parties according to manufacturer instructions.<br><br>For the UK Biobank, FinnGen, MGB, All of Us, HERMES and MVP datasets, data collection and data pre-processing were performed centrally, and therefore no commercial software was needed to collect data specific to the present study.
Data analysis	Processing of genotype data, quality-control, imputation, and genome-wide association analyses were performed with various software tools as described in Supplementary Table 2. Notably, in most of the datasets, various versions of PLINK were used for quality control (https://www.cog-genomics.org/plink/ and https://www.cog-genomics.org/plink/2.0/) and various versions of REGENIE were used for GWAS (https://github.com/rgcgithub/regenie). Meta-analysis of GWAS was performed using the 2011-03-25 release of METAL (https://github.com/statgen/METAL). Heritability and genetic correlation parameters were computed using LDSC version version 1.0.1 (https://github.com/bulik/ldsc). Multi-trait analysis of GWAS was performed using MTAG version 1.0.8 (https://github.com/JonJala/mtag). For Mendelian randomization analyses, we used R-packages TwoSampleMR version 0.5.6 (https://mrcieu.github.io/TwoSampleMR/), coloc version 4.0.4 (https://github.com/chr1swallace/coloc), and CAUSE version 1.2.0 (https://github.com/jean997/cause/tree/master), implemented in custom MR pipelines (https://github.com/seanjosephjurgens/MR_pipeline_sjj). Annotation of GWAS was performed using FUMA version v1.6.1 (https://fuma.ctglab.nl/), as well as MAGMA version 1.10 (https://ctg.cncr.nl/software/MAGMA/prog/magma_v1.10.zip), and PoPS version 0.2 (https://github.com/FinucaneLab/pops). Gene set enrichment analyses were performed using FUMA version 1.6.1 (https://fuma.ctglab.nl/) and g:Profiler version September 20 2023 (https://biit.cs.ut.ee/gprofiler/). For cell type specific heritability analyses, we used R-packages edgeR version 3.22.3 (https://github.com/OliverVoogd/edgeR), DESeq2 version 1.20.0 (https://github.com/thelovelab/DESeq2), and limma version 3.36.2 (https://bioconductor.org/packages/release/bioc/html/limma.html), as well as stratified LDSC version 1.0.1 (https://

github.com/bulik/ldsc). For wrangling of single cell/nucleus data, we used R-package Seurat version 5.0 (https://github.com/satijalab/seurat). For polygenic scoring analyses, we used PRScs version 2022-11 (https://github.com/getian107/PRScs) and PLINK2 (https://www.cog-genomics.org/plink/2.0/; various versions from May 2020 release onwards). All analyses that were run in R, were run in R version 4.0.0.

For manuscripts utilizing custom algorithms or software that are central to the research but not yet described in published literature, software must be made available to editors and reviewers. We strongly encourage code deposition in a community repository (e.g. GitHub). See the Nature Portfolio guidelines for submitting code & software for further information.

Data

Policy information about availability of data

All manuscripts must include a data availability statement. This statement should provide the following information, where applicable:

- Accession codes, unique identifiers, or web links for publicly available datasets
- A description of any restrictions on data availability
- For clinical datasets or third party data, please ensure that the statement adheres to our policy

Summary statistics for our GWAS meta-analyses have been made available for download through the Cardiovascular Disease Knowledge Portal (https://cvd.hugeamp.org/downloads.html); summary statistics for various meta-analyses, including clinical dataset-only and biobank dataset-only, are available (download link: https://api.kpndataregistry.org/api/d/CQyqth). Our PRS scoring weights - for both GWAS and MTAG scores – have been deposited into the PGS Catalog (publication ID: PGP000672; score IDs: PGS004946- PGS004951) and into the Cardiovascular Disease Knowledge Portal (download link: https://api.kpndataregistry.org/api/d/9jevLe). Access to individual-level data for the Meder et al. cohort, the Garnier et al. cohort, the Amsterdam UMC cohort, and MGB will not be made publicly available at this time, due to the restrictive/sensitive nature of the genomic and/or phenotypic data in question. Access to individual level UK Biobank data, both phenotypic and genetic, is available to bona fide researchers through application on the UK Biobank website (https://www.ukbiobank.ac.uk). Access to individual-level phenotypic and genetic data from All of Us Research Program is currently available to bona fide researchers within the United States through the All of Us Researcher Workbench, a cloud-based computing platform (https://www.researchallofus.org/register/). The Finnish biobank data can be accessed through the Fingenious® services (https://site.fingenious.fi/en/) managed by FINBB. Finnish Health register data can be applied for from Findata (https://findata.fi/en/data/). All processed snRNAseq/scRNAseq datasets used in the present study are publicly available: The Chaffin et al. dataset is available for download from the Broad Single Cell Portal (https://singlecell.broadinstitute.org/single_cell/study/SCP1303/single-nuclei-profiling-of-human-dilated-and-hypertrophic-cardiomyopathy); the Reichart et al. dataset was downloaded from GEO (https://www.ncbi.nlm.nih.gov/geo/download/?acc=GSE183852&format=file&file=GSE183852%5FDCM%5FIntegrated%2ERobj%2Egz); the Koenig et al. dataset was downloaded from CellxGene (https://datasets.cellxgene.cziscience.com/3716fb19-cedd-4fe5-abc4-5dbeb007fb65.rds).

Other datasets include cis-eQTLs from the eQTLGen consortium (https://www.eqtlgen.org/cis-eqtls.html); cis-eQTLs from GTEx v8 (https://www.gtexportal.org/home/downloads/adult-gtex#qtl) and tissue expression levels from GTEx v8 (https://www.gtexportal.org/home/downloads/adult-gtex#bulk_tissue_expression); pQTLs derived from the UK Biobank Pharma Proteomics Project (summary statistics for the 'combined' set from https://www.synapse.org/#!Synapse:syn51364943/files/); the 22.10 update of the OpenTargets platform (https://genetics.opentargets.org/); GWAS Catalog queried in October 2023 (https://www.ebi.ac.uk/gwas/); ANNOVAR v2017-07-17 (https://annovar.openbioinformatics.org/en/latest/); 1000 Genomes project Phase 3 (https://www.internationalgenome.org/data/); gnomAD exomes v.2.1 (https://gnomad.broadinstitute.org/downloads); the ClinVar database (https://www.ncbi.nlm.nih.gov/clinvar/) was accessed in April 2023.

Field-specific reporting

Please select the one below that is the best fit for your research. If you are not sure, read the appropriate sections before making your selection.

☒ Life sciences ☐ Behavioural & social sciences ☐ Ecological, evolutionary & environmental sciences

For a reference copy of the document with all sections, see nature.com/documents/nr-reporting-summary-flat.pdf

Life sciences study design

All studies must disclose on these points even when the disclosure is negative.

Sample size

For GWAS discovery and replication analyses, sample sizes were based on the number of samples for which phenotypic and genetic data were available in the datasets at the respective time points, with a clear aim of maximizing effective sample size. No power calculations were performed to pre-determine the required sample size.

Data exclusions

For the discovery datasets, sample exclusions are described for each cohort in detail in Supplementary Table 2. In all discovery datasets, steps were performed to identify samples with bad genetic data (ie, removal of samples with high missingness). In the biobank datasets, samples with general heart failure codes (but not fulfilling case definitions) were removed from the controls in GWAS. In all clinical case-control datasets, exclusions were applied based on imaging data, while in some datasets additional exclusions were applied based on additional disease status. Details can be found in Supplementary Table 2 and Supplementary Note.

For the All of Us WGS dataset, most QC was performed centrally and consisted of per-sample QC, including fingerprint concordance (array vs. WGS data), sex concordance (genetically determined vs. self-reported), cross-individual contamination rate and coverage to detect major errors, such as sample swaps or contamination. Participants who failed these tests were removed from the release. We removed flagged participants (population outliers) and possible duplicates from the current study.

For the MVP replication cohort, exclusions and sample selections are described in the Supplementary Note. For the HERMES datasets, sample selections and exclusions were performed previously, as described in the work of Zheng et al. (2024).

Replication

We performed a replication analysis using a meta-analysis of three datasets, namely All of Us, HERMES, and MVP. This replication meta-analysis included 13,258 DCM/NICM cases and 1,435,287 controls. This replication showed good overall replication, with 92% of GWAS-DCM

	loci reaching P<0.05 and 72% reaching Bonferroni corrected significance; of MTAG-DCM loci, 88% reached P<0.05 and 56% reached Bonferroni-corrected significance. These results show a strong replication trend in our significant loci.
Randomization	Samples were not experimentally randomized, given that the exposure in our analysis is genetic variation.
Blinding	No blinding was performed during analysis of the data. We note that the main discovery analyses represented genome-wide association tests, where all variants reaching quality-control criteria were put forward for meta-analysis and were tested for association. As such, while blinding was not formally applied, the approach should not have been affected by the absence of a formal blinding procedure.

Reporting for specific materials, systems and methods

We require information from authors about some types of materials, experimental systems and methods used in many studies. Here, indicate whether each material, system or method listed is relevant to your study. If you are not sure if a list item applies to your research, read the appropriate section before selecting a response.

Materials & experimental systems

n/a	Involved in the study	
☒	☐	Antibodies
☒	☐	Eukaryotic cell lines
☒	☐	Palaeontology and archaeology
☒	☐	Animals and other organisms
☐	☒	Human research participants
☒	☐	Clinical data
☒	☐	Dual use research of concern

Methods

n/a	Involved in the study	
☒	☐	ChIP-seq
☒	☐	Flow cytometry
☒	☐	MRI-based neuroimaging

Human research participants

Policy information about studies involving human research participants

Population characteristics

In the Meder et al. cohort, all samples were of German-White descent. Among the 909 DCM cases, 25.2% had female genetic sex, the mean age was 56.6 years (SD=12.9), and the mean left ventricular ejection fraction (LVEF) was 28.5 (SD=10.9). Among 2120 controls, 49.7% were of female genetic sex and the mean age was 57.4 (SD=14.1).

The Garnier et al. dataset consisted of samples of European genetically-determined ancestry. Several sub-cohorts comprised the dataset:
French Cardigene cases (N=408): mean age 45.1 (SD=10.7), mean LVEF 24.0 (SD=8.2), mean LVEDD 74.2 mm (SD=9.4), N with heart transplant 212.
French PHRC cases (N=204): mean age 52.0 (SD=13.0), mean LVEF 28.2 (SD=8.9), mean LVEDD 68.5 mm (SD=9.0), N with heart transplant 0.
French Eurogene cases (N=83): mean age 46.9 (SD=13.2), mean LVEF 29.2 (SD=10.2), mean LVEDD 67.5 mm (SD=8.1), N with heart transplant 0.
Italy Eurogene cases (N=82): mean age 43.1 (SD=13.4), mean LVEF 27.2 (SD=7.9), mean LVEDD 66.5 mm (SD=8.5), N with heart transplant 0.
German Eurogene cases (N=214): mean age 45.9 (SD=11.7), mean LVEF 30.3 (SD=11.3), mean LVEDD 69.8 mm (SD=9.7), N with heart transplant 0.
Germany Berlin cases (N=987): mean age 44.0 (SD=11.6), mean LVEF 24.1 (SD=9.7), mean LVEDD 68 mm (SD=10.0).
UK Royal Brompton (N=109): mean age 54.9 (SD=13.4), mean LVEF 30 (SD=8.7), mean LVEDV 347.9 ml (SD=132.6).
US MAGNet cases (N=631): mean age 51.1 (SD=14.2), mean LVEF 20.3 (SD=9.9), mean LVEDD 58.5 (SD=22.3), N with heart transplant 328.
French controls (N=1084) were sourced from the PPS3 study, including 731 with male genetic sex and 353 with female genetic sex, with mean age 62 (SD=6.4).
German controls (N=3,264) were sourced from the population-based KORA F4 study, including 1579 with male genetic sex and 1685 with female genetic sex, with mean age 57.4 (SD=12.9).
Italian controls (N=92) were collected as part of the EHF study, including 70 with male genetic sex and 22 with female genetic sex, with mean age 49.8 (SD=12.7).

The Amsterdam dataset consisted of cases (N=978) from Amsterdam UMC, of which 560 had male genetic sex (57.3%), 783 were of genetically-determined European ancestry (80.6%), with a median LVEF of 28% (Q1,Q3: [20.00, 37.00]), and with median age of 57 years (Q1,Q3: [48.00, 65.00]). Of controls sourced from the Dutch Twin registry (N=7207), 6978 were of genetically-determined European ancestry, and 3172 (44.0%) had male genetic sex.

In the UK Biobank (N=472474), 1065 individuals were identified as cases for nonischemic DCM. 95% of individuals were of genetically-determined European ancestry (94% of cases, 95% of controls), 45% were of male genetic sex (69% of cases, 45% of controls), and mean enrollment age was 57 years (SD=8); of cases mean enrollment age was 60 (SD=7) years, and of controls mean enrollment age was 57 (SD=8) years.

In FinnGen (N=422920), 3550 individuals were identified as cases for nonischemic DCM. 100% of individuals were of genetically-determined Finnish ancestry, 43% were of male genetic sex (74% of cases, 42% of controls), and mean enrollment age was 52 years (SD=18); of cases mean enrollment age was 61 (SD=13) years, and of controls mean enrollment age was 52 (SD=18) years.

In MGB (N=42637), 407 individuals were identified as cases for nonischemic DCM. 84% of individuals were of genetically-determined European ancestry, 42% were of male genetic sex (63% of cases, 41% of controls), and mean enrollment age was 51 years (SD=17); of cases mean enrollment age was 57 (SD=15) years, and of controls mean enrollment age was 51 (SD=17) years.

The All of Us dataset (N= 195533), is an ancestrally-diverse dataset (53% European genetic ancestry, 21% African, 16% Admixed-American ancestry, 1.9% East-Asian ancestry, 0.92% South-Asian ancestry, 0.22% Middle-Eastern ancestry, 8.0% other ancestry), with 75649 (38.7%) being of male genetic sex, and with mean age of 52.4 (SD=16.8). 928 (0.47%) were cases for nonischemic DCM, while 5123 (2.6%) were cases for broad systolic heart failure.

For details on the MVP and HERMES (sub-)cohorts used in replication, please see the Supplementary Note.

Recruitment

In the Meder et al. cohort, cases were ascertained from clinical (cardiological) medical centers in/near Heidelberg, Germany. Controls were sourced from existing population-based reference cohorts.

In the Garnier et al. dataset, all cases were ascertained from clinical (cardiological) medical centers. French controls from PPS3 were ascertained during preventative medical check-ups. German controls from KORA F4 were ascertained from population-based surveys. Italian controls from EHF were healthy health-care workers who were approached to partake in the research work.

In the Amsterdam cohort, cases were ascertained from mining of medical records at the Amsterdam UMC, and by manual review of charts; all patients were referred for genetic testing for DCM within the Amsterdam UMC (a medical center). Controls were ascertained from the Dutch Twin Register; since 1987, this register has been accumulating information on twins and triplets, either when the parents of newborn twins voluntarily register or when adult twins and their family participate.

For UK Biobank, prospective participants were invited to visit an assessment centre, at which they completed an automated questionnaire and were interviewed about lifestyle, medical history and nutritional habits; basic variables such weight, height, blood pressure etc. were measured; and blood and urine samples were taken. These samples were preserved so that it was possible to later extract DNA and measure other biologically important substances. During the whole duration of the study it was intended that all disease events, drug prescriptions and deaths of the participants are recorded in a database, taking advantage of the centralized UK National Health Service.

The FinnGen study (https://www.finngen.fi/en) is an ongoing research project that utilizes samples from a nationwide network of Finnish biobanks and digital healthcare data from national health registers. FinnGen aims to produce genomic data with linkage to health register data of 500,000 biobank participants. Samples in the FinnGen study include ~200,000 legacy samples from previous research cohorts (often disease-specific) that have been transferred to the Finnish biobanks, and ~300,000 prospective samples collected by biobanks across Finland. Prospective samples from six regional hospital biobanks represent a wide variety of patients enrolled in specialized health care, samples from a private healthcare biobank enable enrichment of the FinnGen cohort with patients underrepresented in specialized health care, whereas participants recruited through the Blood Service Biobank enrich the cohort with healthier individuals. Samples have not specifically been collected for FinnGen, but the study has incorporated all that have been available in the biobanks.

For MGB (formerly known as Partners Biobank) samples were prospectively recruited - in an ongoing observational design - from a multicenter health system in Eastern Massachusetts. In MGB, participants are enrolled with broad-based consent collected by local research coordinators, either as part of a collaborative research study or electronically through a patient portal. Demographic data, blood samples and surveys are collected at baseline and linked to electronic health record data.

For All of Us, samples were enrolled in a longitudinal cohort study (with aim of including 1 million racially, ancestrally and demographically diverse participants) from across the United States. Data is prospectively collected, combining phenotypic data from various sources including patient-derived information and electronic health record linkage. One of the goals set by All of Us was to recruit individuals that have been and continue to be underrepresented in biomedical research because of limited access to health care.

For details on the MVP and HERMES (sub-)cohorts used in replication, please see the Supplementary Note.

Ethics oversight

For the Meder et al. dataset, the study was conducted in accordance with the principles of the Declaration of Helsinki. All participants of the study have given written informed consent and the study was approved by the ethic committees of the participating study centers.

For the Garnier et al. dataset, the study protocol was approved by local ethics committees, complied with the Declaration of Helsinki, and all patients signed informed consent.

For the Amsterdam cohort, study of DCM patients from Amsterdam UMC was performed under a waiver - approved by the Medical Ethical Committee of Amsterdam UMC - allowing genotyping and genome-wide association study of individuals affected by cardiovascular disease. The study protocol for GWAS of inherited cardiovascular disease was approved by the local Medical Ethical Review Committee of Amsterdam UMC. For the controls sourced from the Dutch Twin register, Ethical clearance has been granted by the Central Ethics Committee on Research Involving Human Subjects at the VU University Medical Centre in Amsterdam, which is an Institutional Review Board certified by the U.S. Office of Human Research Protections. The approval carries the IRB number IRB-2991 under Federal-wide Assurance-3703 and includes specific institute codes (94/105, 96/205, 99/068, 2003/182, 2010/359). All control participants, or their parents, have given their informed consent to be part of the register.

The UK Biobank resource was approved by the UK Biobank Research Ethics Committee and all participants provided written

informed consent to participate. Use of UKB data was performed under application number 17488 and was approved by the local Massachusetts General Hospital Institutional Review Board.

Participants in FinnGen provided informed consent for biobank research on basis of the Finnish Biobank Act. Alternatively, separate research cohorts, collected before the Finnish Biobank Act came into effect (in September 2013) and the start of FinnGen (August 2017) were collected on the basis of study-specific consent and later transferred to the Finnish biobanks after approval by Fimea, the National Supervisory Authority for Welfare and Health. Recruitment protocols followed the biobank protocols approved by Fimea. The Coordinating Ethics Committee of the Hospital District of Helsinki and Uusimaa (HUS) approved the FinnGen study protocol (number HUS/990/2017). The FinnGen study is approved by the THL (approval number THL/2031/6.02.00/2017, amendments THL/1101/5.05.00/2017, THL/341/6.02.00/2018, THL/2222/6.02.00/2018, THL/283/6.02.00/2019 and THL/1721/5.05.00/2019), the Digital and Population Data Service Agency (VRK43431/2017-3, VRK/6909/2018-3 and VRK/4415/2019-3), the Social Insurance Institution (KELA) (KELA 58/522/2017, KELA 131/522/2018, KELA 70/522/2019 and KELA 98/522/2019) and Statistics Finland (TK-53-1041-17).

Use of All of Us data was approved under a data use agreement between the Massachusetts General Hospital and the All of Us Research Program program.

For MGB, all adult patients provided informed consent to participate. A small number of children were enrolled with IRB-approved assent forms; upon reaching 18 years of age all enrolled children had to provide consent or were removed from the study. The Human Research Committee of MGB approved the Biobank protocol (2009P002312).

All MVP and HERMES (sub-)cohorts collected appropriate participant consents, and protocol approvals where appropriate.

Note that full information on the approval of the study protocol must also be provided in the manuscript.
